# Cavity-modified exciton–exciton annihilation in disordered molecular systems

**DOI:** 10.1039/d6cp02032a

**Published:** 2026-08-21

**Authors:** Ilia Sokolovskii, Ben S. Humphries, Jochen Blumberger, Gerrit Groenhof

**Affiliations:** a Department of Physics and Astronomy and Thomas Young Centre, University College London Gower Street London WC1E 6BT UK i.sokolovskii@ucl.ac.uk; b Nanoscience Center and Department of Chemistry, University of Jyväskylä P.O. Box 35 40014 Jyväskylä Finland

## Abstract

Recent experiments have shown contradictory effects of strong light–matter coupling on exciton–exciton annihilation (EEA) in organic molecular systems. In this work, we perform numerical simulations of polariton dynamics and reveal the role of strong coupling in changing the EEA rate. The results of our simulations suggest that strong coupling allows to partially overcome disorder in systems with poor exciton mobility *via* delocalisation of excitons owing to the interaction with the common cavity mode. This leads to an enhanced connectivity between excitons and, consequently, to an increase in the EEA rate. Conversely, in systems with high exciton mobility, in which disorder has a much smaller effect on excitation energy transfer, excitons can interact strongly even without coupling to the cavity photons at the exciton densities at which EEA typically occurs. In this case, the EEA rate can be even lower than in bare molecules due to the existence of a competing decay channel associated with photon leakage through the cavity mirrors. We also find that in the weak coupling regime, the EEA rate appears to be suppressed due to this decay channel regardless of the exciton transport properties. Our simulations resolve the experimental controversy on the effect of strong coupling on EEA and provide guidance for minimising the EEA rate towards a more feasible realisation of Bose–Einstein condensation of polaritons.

## Abstract 2

EEA typically occurs between neighbouring molecules since its efficiency rapidly decays with the intermolecular distance. Strong light-matter coupling allows for more efficient annihilation through the formation of delocalised polariton states.

## Graphical abstract

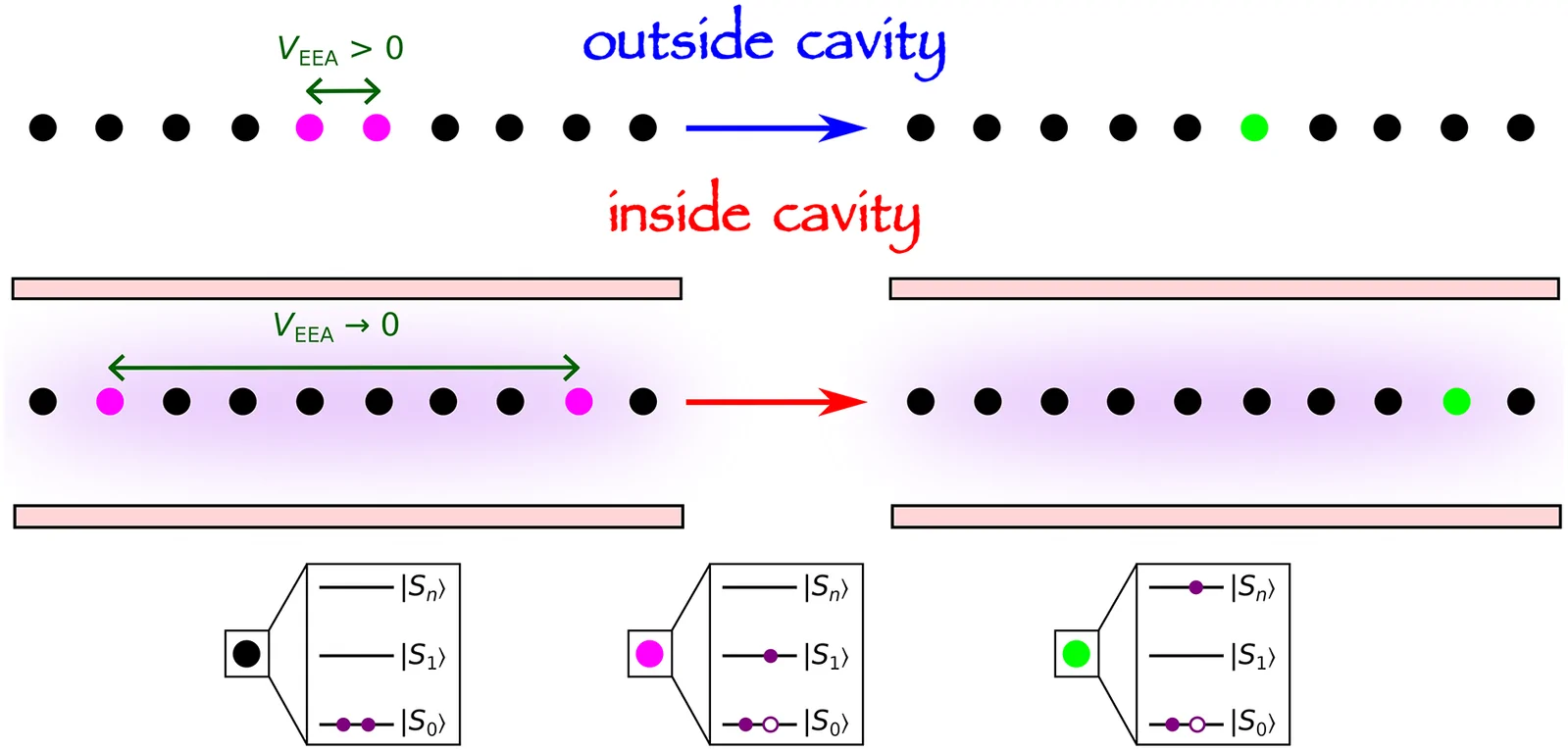

## 1 Introduction

Strong coupling of organic molecules with confined electromagnetic fields in optical resonators opens the potential for improving the efficiency of organic solar cells,^1–3^ building logics for classical and quantum computers,^4^ modifying photochemistry,^5^ and achieving coherent light emission.^6^ In particular, polariton lasers represent a low-threshold alternative to conventional lasers, which rely on population inversion and have a high threshold for the injected charge carrier density required to achieve lasing.^7^ In contrast, polariton lasers rely on Bose–Einstein condensation (BEC), at which a macroscopic number of polaritons occupies the same quantum state,^8^ usually the bottom of the lower polariton (LP) branch but not necessarily.^9^ This leads to bosonic stimulation of polaritons, when the transition probability into the common polaritonic state grows linearly with the occupation of this state. Bosonic stimulation is followed by nonlinear amplification, when the emission rate from the occupied state increases non-linearly as the density of polaritons crosses a certain threshold. Owing to a very small effective mass of polaritons, inherited from their partially photonic character, a room-temperature BEC of polaritons is possible, as was first demonstrated by Kéna-Cohen and Forrest in a single-crystal anthracene microcavity.^10^

Although organic polariton lasers, which do not require population inversion, allow for lower threshold charge densities compared to conventional photon lasers,^10,11^ an electrically injected laser, which is highly desirable for practical applications, is still beyond reach. This is due to the low mobility of charge carriers in organic materials, singlet/triplet statistics, as well as the effects competing with the population relaxation to the bottom of the LP branch.^12^ It is commonly assumed that the relaxation to this state from the reservoir of excitons occurs by the following two mechanisms: radiative pumping of polaritons by uncoupled molecules^13,14^ and vibrational-assisted scattering, in which part of the exciton energy is transferred to a specific molecular vibration.^15–18^ The rate of both mechanisms was previously found to scale inversely with the number of strongly coupled molecules.^19–25^ Recently, Pérez-Sánchez and Yuen-Zhou also proposed to classify polariton relaxation into radiative pumping, polariton-assisted photon recycling, and polariton-assisted Raman scattering.^26,27^

**Fig. 1:**
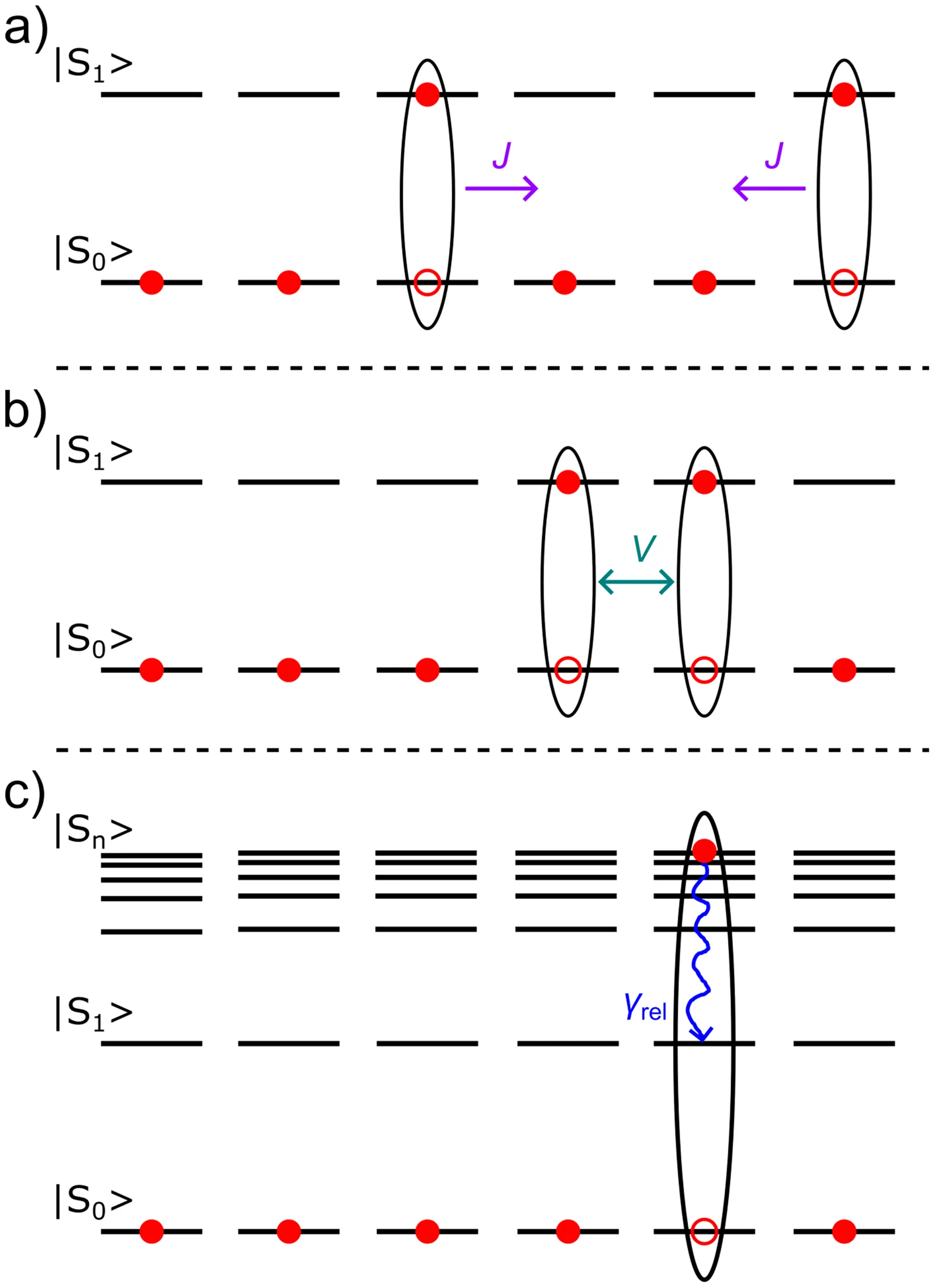
Schematic illustration of the mechanism of exciton–exciton annihilation (EEA). In a chain of molecules, represented as multiple energy levels (|S_0_〉,|S_1_〉,…,|S_*n*_〉,…), a pair of S_1_-excitons with filled and open circles representing electrons and holes, respectively, is initially created (panel a). These excitons can hop between adjacent molecules *via* the dipole–dipole coupling or wave functions overlap, characterised by coupling strength *J*. When the two excitons approach each other, they can interact *via* coupling term *V*, which describes the excitonic interaction (panel b). As a result of this interaction, an electron in one of the neighboring molecules can be promoted to the S_*n*_-state with energy *E*_S_*n*__ ≈ 2*E*_S_1__ (panel c). At the same time, the other molecule returns to the ground state. Subsequently, the first molecule rapidly relaxes into the S_1_-state, which is characterised by relaxation rate *γ*_rel_.

Relaxation from the exciton reservoir to the bottom of the LP branch always competes with deactivation processes that deplete the population of polariton states. These deactivation processes include decay of polaritons due to their partially photonic nature and exciton–exciton annihilation (EEA).^28^Fig. 1 schematically demonstrates the principle of EEA. For a pair of S_1_-excitons created at a certain distance from each other in a material, each of these excitons can hop independently between adjacent molecules *via* the Förster^29^ or Dexter^30^ mechanism, characterised by small values of coupling strength *J* (panel a) compared to reorganization energy, or by transient delocalization for larger values of *J*.^31–35^ Once the excitons approach each other in space (panel b), the excitonic interaction, *V*, may promote an electron in one of the molecules to the S_*n*_-state with energy *E*_S_*n*__ ≈ 2*E*_S_1__ while simultaneously de-exciting the other molecule. The molecule in the S_*n*_-state then rapidly relaxes into the S_1_-state according to Kasha's rule^36^ (panel c). Therefore, as a result of the interaction, a pair of S_1_-excitons is converted into a single S_1_-exciton, for which reason this type of interaction is usually called exciton–exciton annihilation.

If depopulation of the LP branch due to polariton decay and EEA occurs faster than relaxation to the bottom of the LP branch, condensation of polaritons and, consequently, lasing will not be achieved. While the cavity decay rate can be controlled to some extent by the cavity quality factor (*Q*-factor), the EEA rate is material-specific and is known to increase with charge carrier density.^37^ Therefore, EEA can be a serious parasitic effect for polariton lasers, in which high exciton densities are required to achieve condensation.^38^ Furthermore, since polaritons possess a partially excitonic character, the emergence of strong coupling itself can alter the EEA rate. Understanding the mechanism of this effect and whether the rate of EEA can be increased or reduced in the cavity is therefore important for improving organic polariton lasers towards the development of an electrically pumped laser.

The first experimental study of the effect of strong coupling on exciton annihilation was conducted by Akselrod *et al.* for J-aggregates of cyanine dye in a Fabry–Pérot microcavity.^39^ Photoluminescence measurements revealed an increase in the EEA rate under strong coupling, which was attributed to an enhanced effective exciton diffusion length. Recently, Wu *et al.* demonstrated a reduction in the threshold exciton density for the onset of EEA for photosynthetic light-harvesting 2 (LH2) complexes and Rhodamine 6G (R6G) dye in a planar metallic cavity using pump–probe spectroscopy.^40^ The authors explained this result by improved excitation energy transfer due to strong coupling. In another experiment with R6G, this time in a high *Q*-factor distributed Bragg reflector microcavity, Qureshi *et al.* employed angle-resolved photoluminescence measurements and obtained the opposite result: the threshold exciton density for the onset of EEA was found to increase in the cavity.^41^ The suppression of the EEA was attributed to (i) a more efficient depopulation of the exciton reservoir through cavity decay, (ii) an increase in the propagation velocity of excitons, which facilitates their escape from neighbouring excitons, and (iii) a more efficient coupling of excitons with the cavity mode than with each other. Furthermore, suppression of the EEA rate was recently demonstrated in a two-dimensional perovskite (PEA)_2_PbI_4_ in a Fabry–Pérot microcavity^42^ and in a monolayer of WS_2_, in which excitons were intermediately coupled to Mie resonances of a dielectric nanoantenna.^43^ These results were attributed to the hybrid nature of polaritons and an increase in the spontaneous emission rate of the material through the Purcell effect. Finally, no modification of EEA was reported in a recent study of polycrystalline zinc phthalocyanine strongly coupled to surface-plasmon polaritons.^44^

To reconcile these conflicting experimental results and study the effect of the cavity-modified EEA, we performed numerical simulations of polariton dynamics in the second excitation subspace, with the inclusion of interaction terms responsible for exciton–exciton annihilation. The results of the simulations indicate a competition between two mechanisms: cavity-enhanced exciton delocalisation, which leads to better connectivity between excitons in the presence of disorder and, therefore, enhances the EEA rate, and polariton decay, which depletes the excited-state population, reducing exciton collisions; therefore, this effect decreases the EEA rate. Thus, the overall positive or negative influence of a cavity on the EEA rate is determined by the relationship between the magnitudes of disorder strength, of exciton coupling, and of cavity decay rate in each specific molecule-cavity system. This implies that a careful selection of the strongly coupled material and the cavity structure is required to achieve a balance between the rates of EEA and of polariton decay in order to minimise the threshold for BEC of polaritons.

## 2 Theoretical model

### 2.1 System's description and model Hamiltonian

Following previous theoretical studies of exciton–exciton annihilation,^45,46^ we consider a closed chain of *N* three-level molecules (TLMs) with the following electronic states: the ground state with energy *E*_S_0__, the first excited state with energy *E*_S_1__, and the *n*th excited state with energy *E*_S_*n*__ = 2*E*_S_0__. In such a system, two types of excitons can be created in each molecule, namely S_1_-excitons and S_*n*_-excitons. Furthermore, S_1_-excitons can hop between adjacent molecules due to exciton coupling, characterised by strength *J*, and a pair of such excitons can interact through the coupling term *V*, resulting in EEA. The S_1_-excitons are also coupled to the fundamental cavity mode with energy *E*_c_ = *E*_S_1__, and the coupling is characterised by strength *g*. Since the oscillator strength of the S_*n*_-state is often significantly smaller compared to the S_1_-state, as is also the case for R6G molecule used as a model system in this work (Section 3 in the SI), the S_*n*_-state is assumed to interact with the second cavity mode of energy *E*_c2_ = *E*_S_*n*__ very weakly. Therefore, we do not include the corresponding coupling term in the model. Based on the same reasoning about oscillator strength, we also assume that the dipole–dipole interaction between S_*n*_-states is very weak and therefore neglect intermolecular transfer of the S_*n*_-excitons.

The molecule-cavity system is described by the following Hamiltonian:1
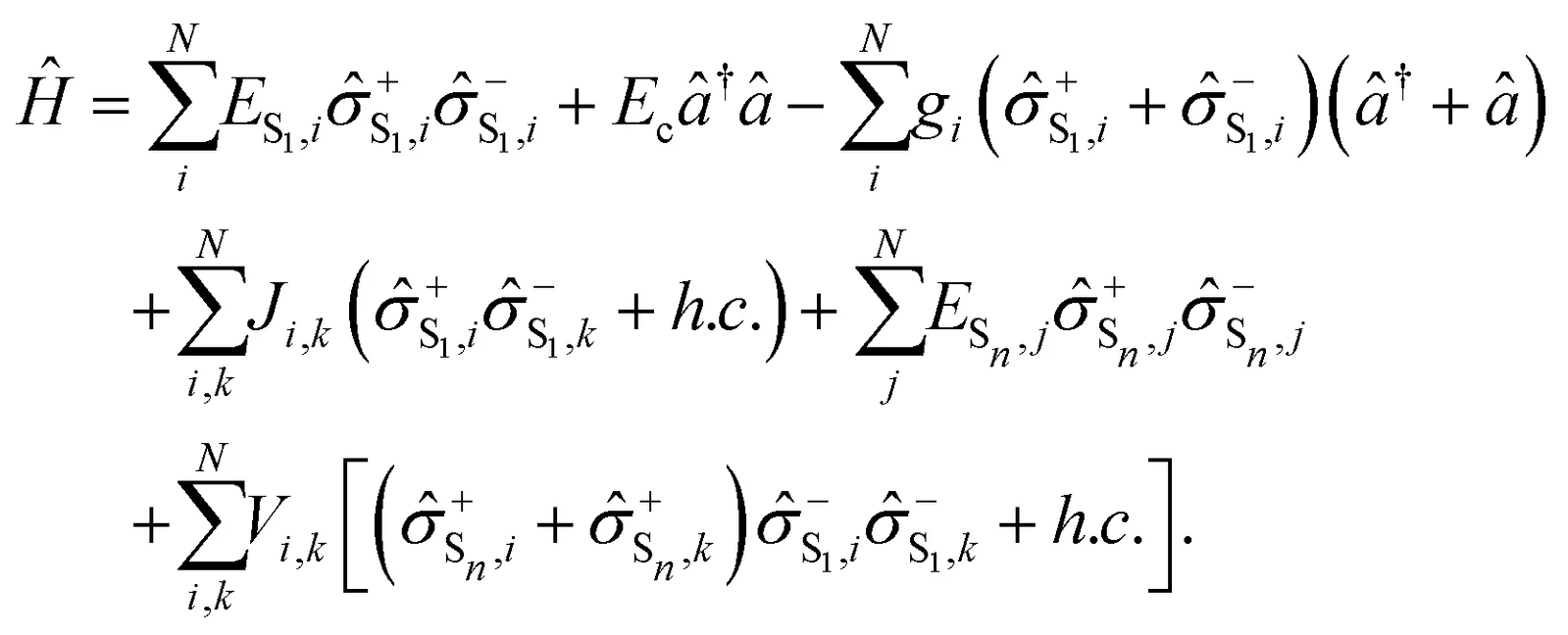


Here, the first line corresponds to the standard Dicke Hamiltonian that describes the interaction between several molecules and one cavity mode.^47^ Operators 
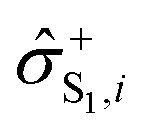
 and 
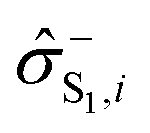
 create and destroy a S_1_-exciton in molecule *i*, whereas operators *â*^†^ and *â* create and annihilate a cavity photon. The second line in eqn (1) describes the interaction between TLMs *i* and *k*, which allows S_1_-excitons to jump between the corresponding molecules.^48^ In the last line of eqn (1), the first term contains the energies of S_*n*_-excitons in each TLM, and the second term describes EEA in TLMs *i* and *k*, which results in the destruction of S_1_-excitons at both TLMs (operators 
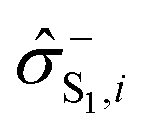
 and 
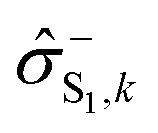
) and the simultaneous formation of a S_*n*_-exciton at either TLM *i* or TLM *k* (operators 
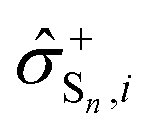
 and 
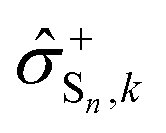
).^46,49,50^ Furthermore, the coupling elements *J*_*i*,*k*_ and *V*_*i*,*k*_ responsible for the transfer of S_1_-excitons and EEA, respectively, are assumed to decay with the distance as *J*_*i*,*k*_ = *J*/|*i* − *k*|^3^ and *V*_*i*,*k*_ = *V*/|*i* − *k*|^3^, according to the point dipole approximation.^51^

Because EEA involves interaction of two excitons, the Hamiltonian matrix is constructed up to the second excitation subspace (Section 1 in the SI). Within the Rotating-wave approximation (RWA), which is valid below ultrastrong coupling, *i.e.* when 
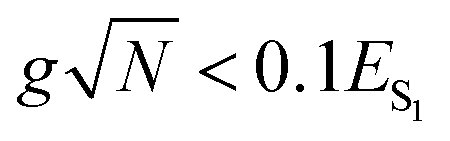
,^52^ and which we employ in this work, the counter-rotating terms, 
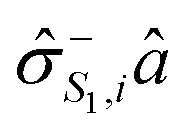
 and 
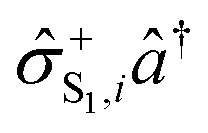
, are neglected, and hence the zeroth excitation subspace is no longer directly coupled to the second excitation subspace. Therefore, for the purpose of this study, we may only consider the part of the Hamiltonian that corresponds to the second excitation subspace. In this subspace, four types of product states are distinguished: (i) 
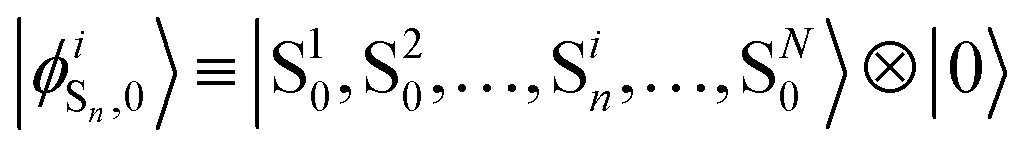
, which describes the situation in which TLM *i* is in the S_*n*_-state, while the other TLMs are in the ground state and no cavity photon is present; (ii) 
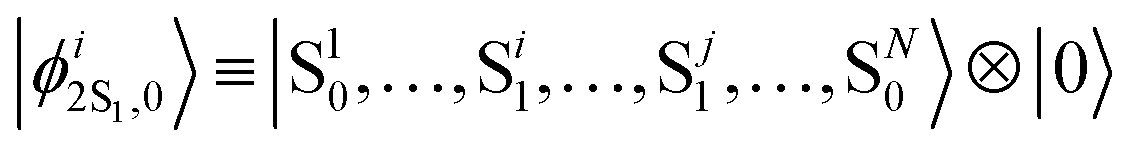
, which is the state with simultaneously excited TLMs *i* and *j* into the *S*_1_-state, while the other TLMs are in the ground state and no cavity photon is present; (iii) 
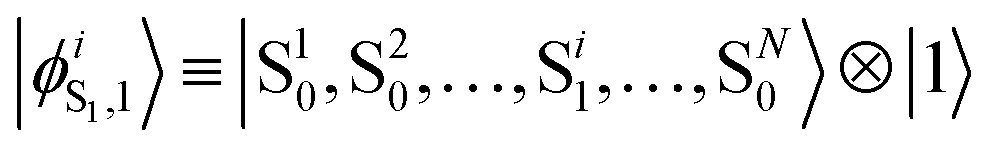
, which describes the situation in which TLM *i* is in the S_1_-state and a single photon is present in the cavity; and (iv) 
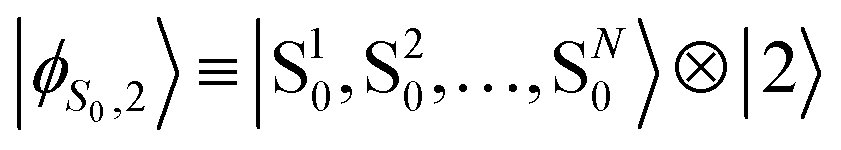
, which corresponds to two photons present in the cavity, while all TLMs are in the ground state. In total, there are 
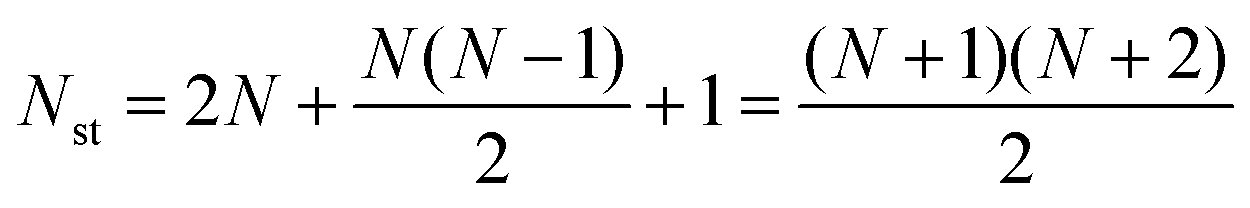
 states, *N* of which are 
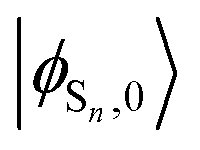
 states, *N*(*N* − 1)/2 states are 
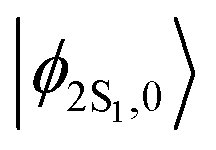
 states, another *N* states are 
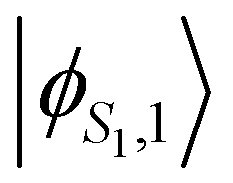
 states, and the remaining state is the 
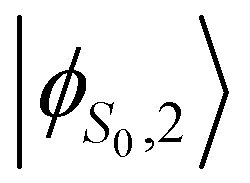
 state.

Following Ryzhov *et al.*, we assume that EEA leads to the promotion of an electron in one of the two interacting TLMs to an excited vibrational state of the *n*th electronic state, *i.e.* |S_*n*_,*v* > 0〉.^46^ From there, efficient phonon-assisted relaxation to the vibrational ground state, |S_*n*_,*v* = 0〉, occurs, followed by relaxation to the S_1_-state in accordance with Kasha's rule.^36^ Subsequently, the TLM can also decay to the ground state *via* radiative or nonradiative pathways. Modelling internal conversion to the S_1_-state requires the use of advanced methods of quantum or semiclassical dynamics, which is intractable for the system under consideration. Therefore, we instead approximate the three relaxation processes (phonon-assisted relaxation within the S_*n*_-state, internal conversion to the S_1_-state, and decay to the ground state) as a single process by introducing the deactivation terms, −*iħγ*_v_, from the Lindblad operator to the diagonal elements of the system's Hamiltonian (eqn (1)) that correspond to the 
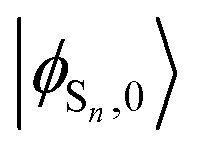
 states.^53,54^ In other words, we assume that after internal conversion to the S_1_-state and hence entering the first excitation subspace, a TLM becomes decoupled from the second excitation subspace and can only decay to the ground state. Future work will aim at removing this approximation and simulating exciton annihilation including not only the second but also the zeroth and first excitation subspaces explicitly.

With the deactivation terms and under the RWA, the matrix form of the Hamiltonian in the second excitation subspace reads as follows:2
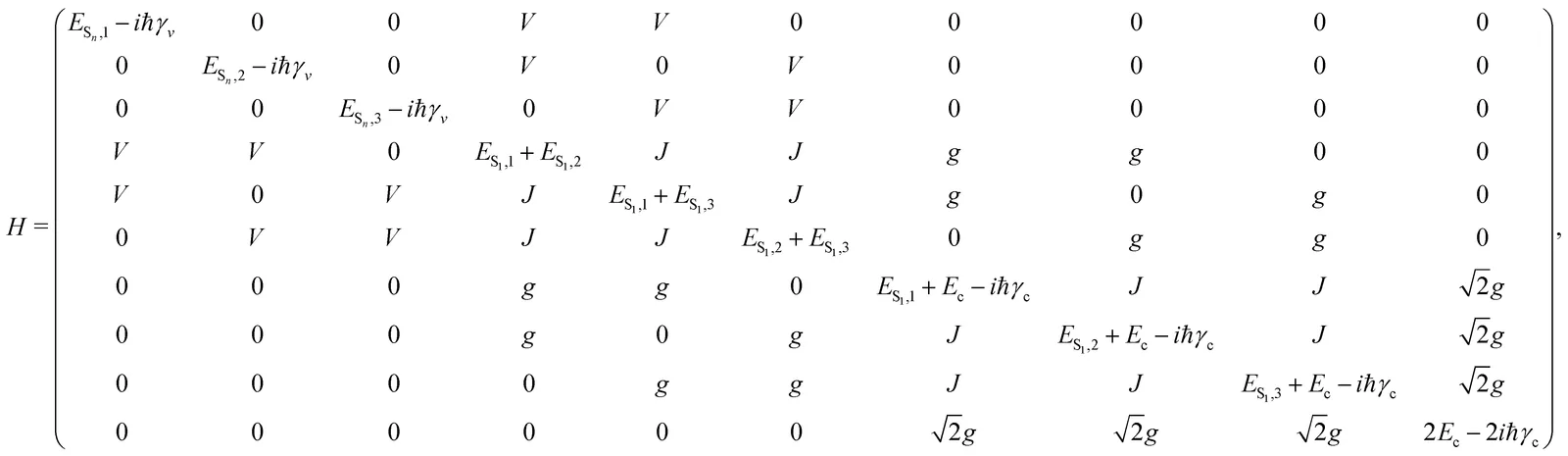
where, to save space, the number of TLMs is selected to be *N* = 3. Additionally, deactivation terms −*iħγ*_c_ and −2*iħγ*_c_ with *γ*_c_ being the cavity decay rate, are added to the diagonal elements corresponding to states 
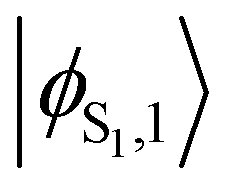
 and 
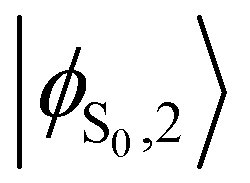
, respectively, to account for photon decay due to the imperfections of the cavity mirrors. We also note that the light-matter coupling terms corresponding to the exchange of a photon between states 
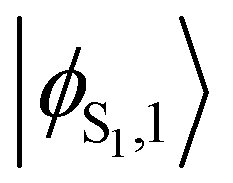
 and 
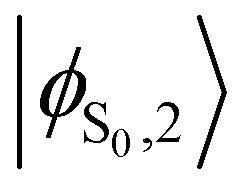
 (*i.e.* the terms in the last row and last column of the Hamiltonian matrix in eqn (2)) are multiplied by the square root of two since 
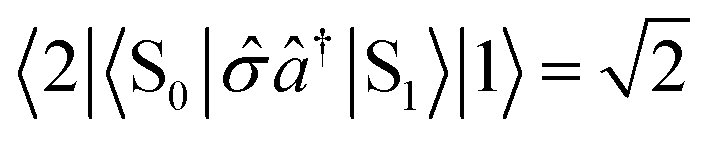
.

Diagonalisation of the Hamiltonian in eqn (2) yields adiabatic light–matter states, which can be divided into three types^55,56^: multi-polariton states with a significant photonic content, dark polariton states with a small but nonzero photonic content, and dark states without cavity contribution (Fig. S1 in the SI). In the absence of energetic disorder, decay channels (*γ*_v_ = 0 and *γ*_c_ = 0), exciton and EEA couplings (*J* = 0 and *V* = 0), the splitting between the multi-polariton states is given by 
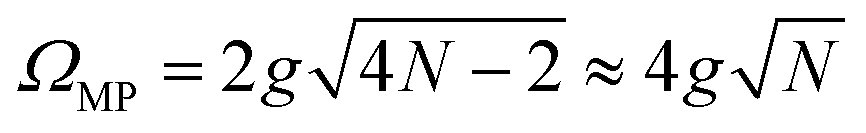
, *i.e.* approximately twice as high as the Rabi splitting in the first excitation subspace, 
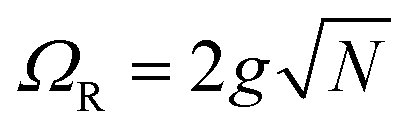
 (Section 2 in the SI). At the same time, the splitting between dark polariton states is slightly smaller than the Rabi splitting and is given by 
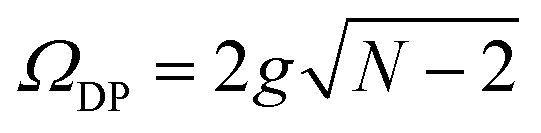
.

To simulate the dynamics of polaritons, the total polariton wave function is constructed as a linear combination of the product states:3
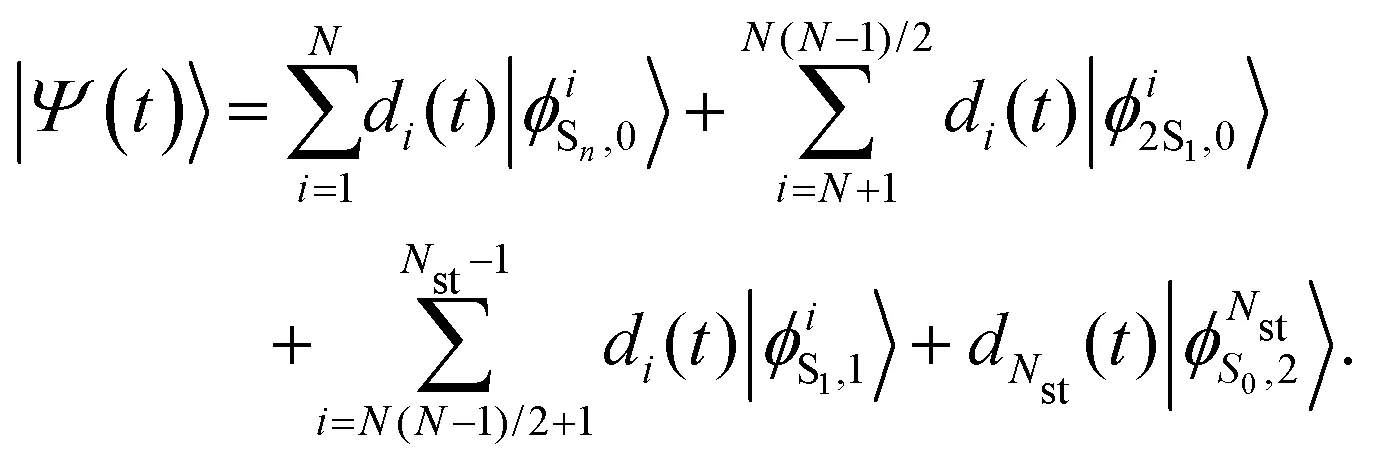


The expansion coefficients, *d*_*i*_(*t*), are propagated by numerical integration of the Schrödinger equation over discrete time intervals, Δ*t*,

4 ***d***(*t* + Δ*t*) = exp(−*i**H***Δ*t*/*ħ*)***d***(*t*).

Because of the deactivation terms, −*iħγ*_v_, −*iħγ*_c_ and −*i*2*ħγ*_c_, the norm of the total wave function, 
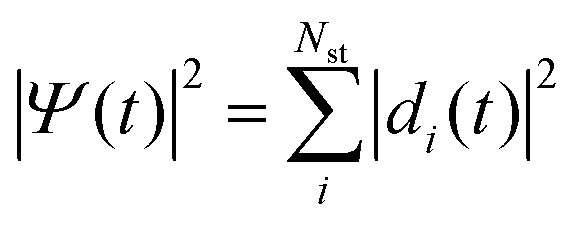
 (eqn (3)), is not conserved and decreases with time. Therefore, population of the ground state (GS), 
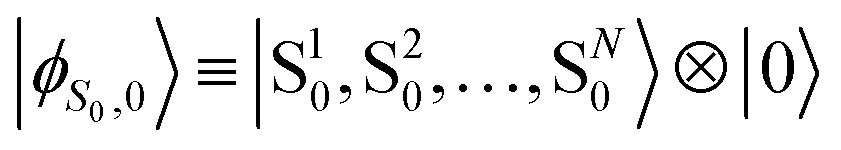
, is determined as *P*_GS_(*t*) = 1 − |*Ψ*(*t*)|^2^ at each time step. Furthermore, the total populations of 
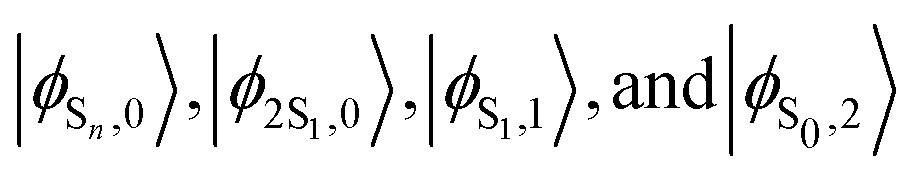
 states are defined as 
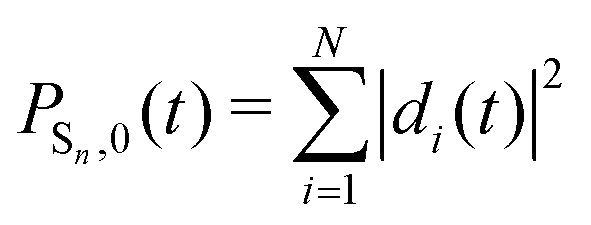
, 
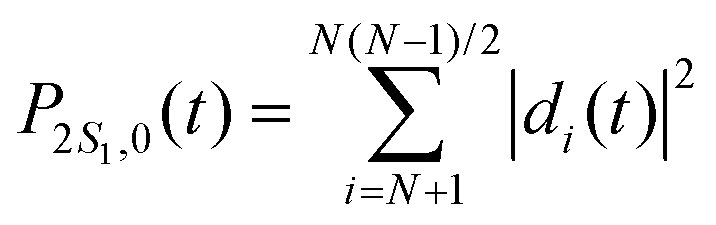
, 
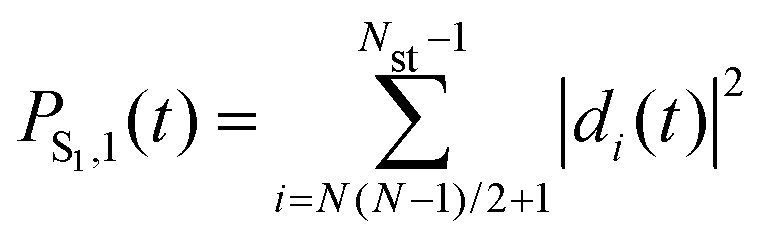
, and 
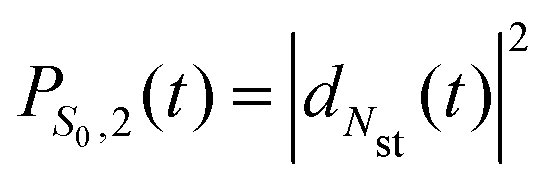
, respectively.

Eqn (4) describes the evolution of a closed quantum system, in which the decay to the ground state is modeled implicitly with imaginary deactivation terms. To model this process explicitly and take a weak Markovian interaction with the environment into account, the Lindblad master equation can be employed^53,57^:5
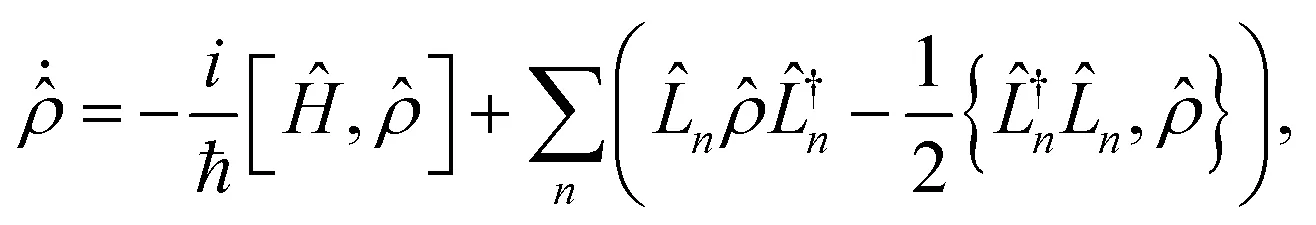
where 
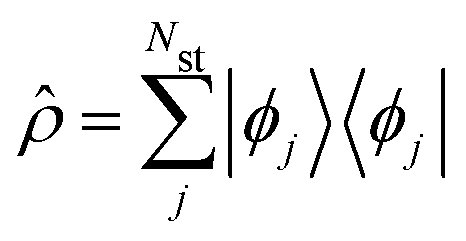
 is the system's density matrix with 
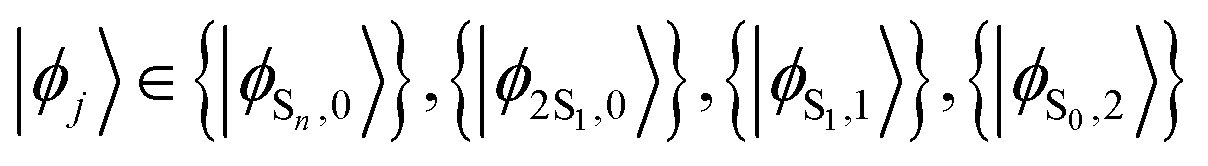
. Operators *L̂*_*n*_ and 
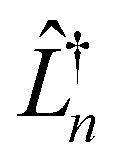
 are the so-called Lindblad jump operators, which describe the dissipation of energy to the environment and the loss of coherence. Here, we consider the first effect by including the cavity decay operators 
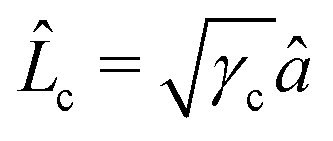
 and 
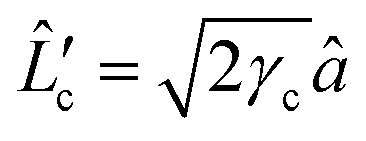
 acting on the states 
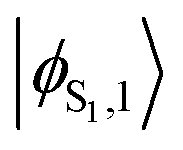
 and 
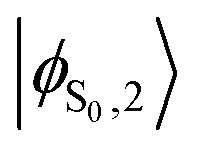
, respectively, as well as the operator 
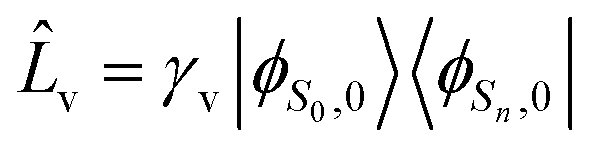
, which deactivates the S_*n*_-excitons. Additionally, pure dephasing processes due to local environmental fluctuations are described through the operators 
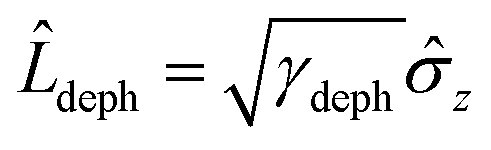
 acting on the states with one TLM excited, *i.e.*
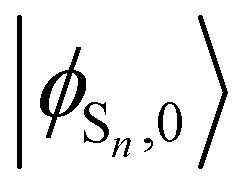
 and 
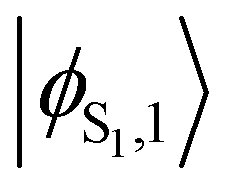
, and 
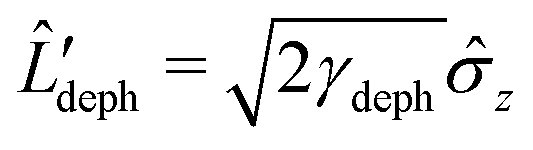
 acting on the states with two TLMs excited, *i.e.*
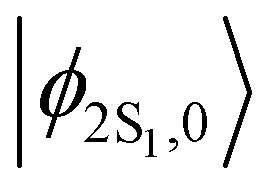
. The local pure-dephasing model employed here may be viewed as a Markovian description of dynamical disorder, corresponding to spatially and temporally uncorrelated fluctuations of the molecular excitation energies. It does not, however, account for finite bath correlation times or impose finite-temperature detailed balance. These effects could be addressed using microscopic open-system approaches such as the hierarchical equations of motion (HEOM),^58^ which treat coupling to a harmonic environment nonperturbatively, albeit at substantially higher computational cost. Alternatively, mixed quantum-classical molecular-dynamics approaches can explicitly account for time-dependent fluctuations of molecular excitation energies and intermolecular couplings arising from nuclear motion.^59–63^

### 2.2 Simulation details

To study how strong coupling modifies the EEA rate in systems with different exciton mobilities and concentrations, we simulated polariton dynamics over a broad range of numbers of molecules, from *N* = 10 to *N* = 100, and exciton coupling strengths, from 〈*J*〉 = 5 meV to 〈*J*〉 = 150 meV. The maximum value of the exciton coupling corresponded to the time-averaged value obtained from molecular dynamics (MD) simulations of the H-dimer of R6G,^64^ the chromophore molecule used in the experiments of Wu *et al.*^40^ and Qureshi *et al.*^41^ on cavity-modified EEA. This value can be thought of as an extreme case of high molecular concentration leading to the formation of aggregates. The details of the MD simulations are discussed in Section 3 of the SI.

In realistic systems, molecules never have exactly the same energies. Therefore, static disorder was introduced by randomly sampling excitation energies of TLMs from the Gaussian distribution:6
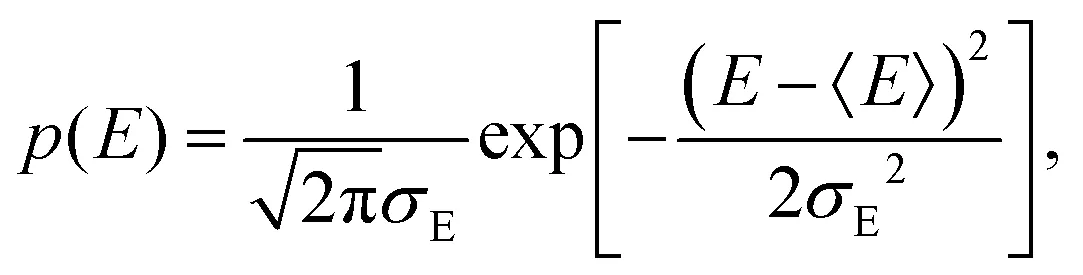
where, for the S_1_-state, 〈*E*〉 = 2.3 eV was the mean value corresponding to the absorption maximum of R6G in aqueous solutions,^65^ and *σ*_E_ was the disorder strength, previously estimated to be around 100 meV from quantum mechanics/molecular mechanics (QM/MM) simulations of Rhodamine molecules in water,^66^ and we used this value in all simulations unless otherwise stated. The energy of the S_*n*_-state of each TLM was also randomly taken from eqn (6) and then multiplied by two.

The coupling terms responsible for exciton hops and EEA were also randomly drawn from the Gaussian distribution with the corresponding means 〈*J*〉 and 〈*V*〉 and standard deviations *σ*_*J*_ and *σ*_*V*_, respectively. All results presented in the main text were obtained with the following values: 〈*V*〉 = 20 meV, *σ*_*J*_ = 10 meV, *σ*_*V*_ = 10 meV. We note that because there are several high-energy states nearly in resonance with the double energy of the S_1_-state, 2*E*_S_1__, all of these states can contribute to EEA, effectively increasing the EEA rate compared to a single S_0_ → S_*n*_ transition. Furthermore, for some of these states, the transition dipole moments (TDMs) can undergo significant fluctuations during dynamics, leading to large fluctuations in the corresponding coupling terms (Fig. S2). We emphasize that the linear chain considered here is intended as a minimal model rather than a literal structural representation of the experimental R6G system. In realistic molecular assemblies, which are typically disordered or amorphous, intermolecular distances and orientations are broadly distributed, which in turn leads to a distribution of excitation energies and coupling elements. Therefore, we conducted additional simulations of polariton dynamics with elevated EEA coupling strength and its mean, namely 〈*V*〉 = 50 meV and *σ*_*V*_ = 50 meV, and confirmed that the conclusions drawn in this study are independent of the choice of 〈*V*〉 and *σ*_*V*_ (Fig. S3).

Although the present model employs a one-dimensional linear chain, structural and energetic heterogeneity typical of realistic amorphous molecular assemblies is incorporated phenomenologically through disorder in the site energies and intermolecular couplings. By varying the ratio 〈*J*〉/*σ*_E_, the model samples transport regimes ranging from strongly localised to relatively delocalised exciton motion.

The time propagation of the total wave function according to eqn (4) was run for *t* = 2 ps with a time step of Δ*t* = 1 fs. The latter is smaller than the shortest time scale in the dynamics of the light–matter system, namely the period of Rabi oscillations, and therefore is small enough to capture all the relevant physical effects. Indeed, performing simulations with the chosen time step gives the same populations of product states as in simulations with a shorter time step of Δ*t* = 0.1 fs (Fig. S4). In all simulations, the dynamics were initialised in a two-exciton product state 
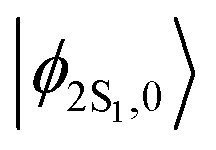
 with the expansion coefficient *d*_*i*_(0) = 1, where *N* < *i* ≤ *N*(*N* − 1)/2, and the starting positions of the two excitons were selected either randomly or to correspond to the greatest distance between TLMs in the chain. The energy of the cavity photon was chosen to be in resonance with the average value of the S_1_-state, *i.e. E*_c_ = 〈*E*〉 = 2.3 eV. As we show in Fig. S5 in the SI, cavity detuning has only a minor effect on the population dynamics of exciton-polaritons in our model. In simulations without cavity decay (*γ*_c_ = 0), the collective light-matter coupling strength was set to 
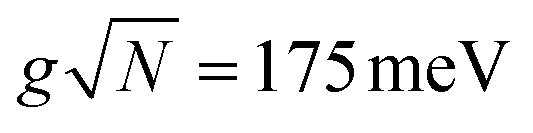
, which corresponded to the Rabi splittings achieved for R6G in a metallic and DBR microcavity in the experiments on cavity-modified EEA.^40,41^ In simulations with cavity decay (*γ*_c_ ≠ 0), the collective coupling strength was reduced to 
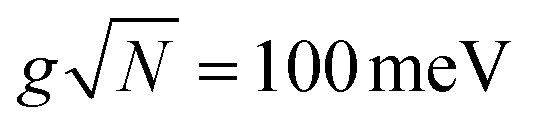
 in order to investigate whether the suppression/enhancement of the EEA rate can be achieved not only in the strong coupling regime but also in the weak coupling regime, *i.e.* when 
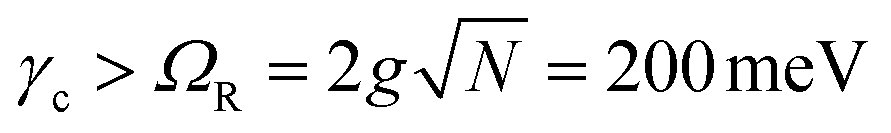
 – or, equivalently, *τ*_c_ < 1/*Ω*_R_ = 21 fs, with *τ*_c_ = 1/*γ*_c_ being the cavity lifetime. Accordingly, the excitation energy disorder strength was reduced to *σ*_E_ = 57.14 meV to retain the same ratio between the collective light-matter coupling strength and the disorder strength, 
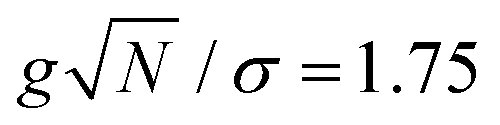
, as in simulations without cavity decay. In all simulations, the individual couplings, *g*, were kept the same for all TLMs. We also tested orientatial disorder by sampling the sigle-molecule light–matter couplings from a Gaussian distribution. For the parameters considered here, this had only a minor effect on the GS population dynamics and was therefore neglected in the main simulations. For the deactivation rate of the S_*n*_-state, we chose a value of *γ*_v_ = 41.36 meV, corresponding to a lifetime of *τ*_v_ = 100 fs, which is within the sub-picosecond timescale typical for internal conversion of organic dyes to the S_1_-state.^67^ All simulations were repeated 1000 times with different realisations of the disordered Hamiltonian (*σ*_E_, *σ*_*J*_ and *σ*_*V*_) to ensure convergence of the populations of the product states and of the ground state.

Simulations of molecular systems with dephasing in the Lindblad formalism were performed with *N* = 10 and 20 TLMs for *t* = 2 ps with a time step of Δ*t* = 50 as and averaged over 100 disorder realisations. The dephasing rate was chosen to be *γ*_deph_ = 41.36 eV, which corresponded to a coherence lifetime of *τ*_deph_ = 100 fs. Because the size of the density matrix in eqn (5) scales as *N*_st_^2^ with the number of product states, modelling molecular ensembles with *N* > 20 becomes computationally prohibitive (see Section 5.2 in the SI for discussion). To avoid this problem and perform simulations of larger molecular ensembles, instead of solving the Lindblad master equation, the total wave function can be propagated by numerical integration of the Schrödinger equation (eqn (4)) with stochastic Markovian jumps associated with the Lindblad operators.^23,68–71^ This so-called Monte Carlo wave function (MCWF) method allowed us to run the dynamics of *N* = 50 TLMs with the inclusion of dephasing. Because the method requires averaging not only over disorder realisations but also over stochastic quantum trajectories, the number of runs was increased to 10 000. In Section 5 of the SI, we provide a detailed description of simulations with both the Lindblad master equation and MCWF, as well as validate the MCWF approach against a direct solution of the Lindblad master equation for systems with *N* = 10 and *N* = 20.

Simulations of polariton dynamics were conducted with our home-built Python script. The geometry optimisation of the H-dimer of R6G was performed in ORCA 6.0,^72^ and the MD simulations were performed in NAMD 3.0.^73^ The coupling terms *J* and *V* were calculated using the transition electrostatic potential charges, which were in turn computed with Multiwf,^74,75^ and the atomic coordinates were extracted from MD simulations with MDAnalysis.^76,77^

## 3 Results and discussion

**Fig. 2:**
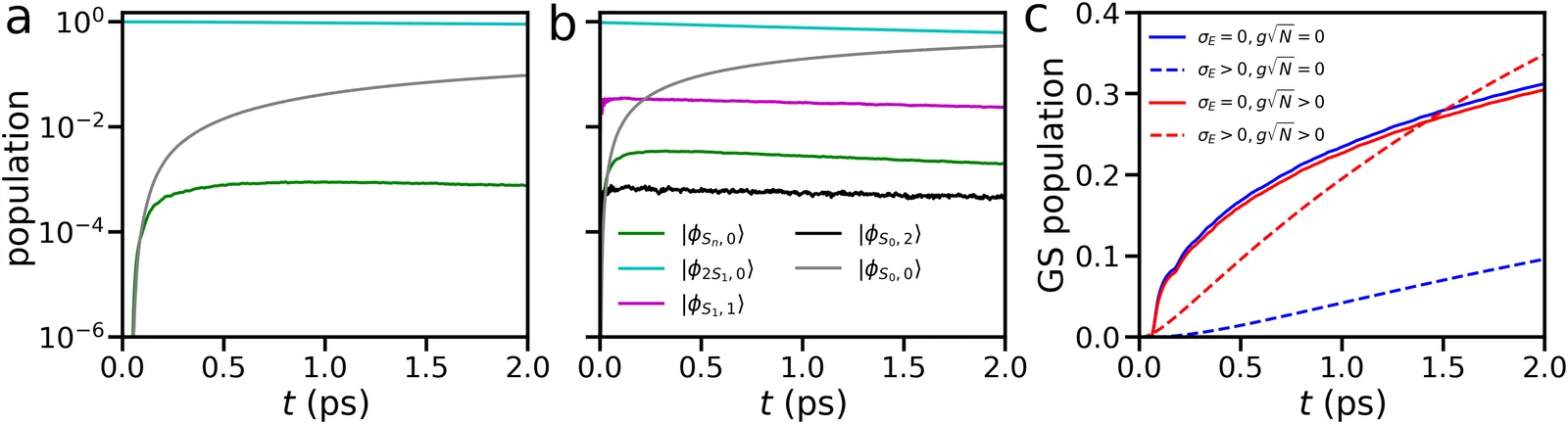
Panels a and b: total populations of all 
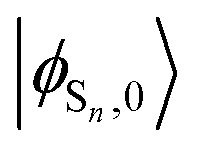
 states (green), 
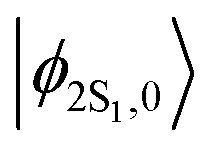
 states (cyan), 
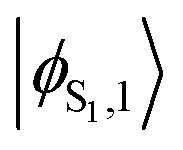
 states (purple), as well as of the 
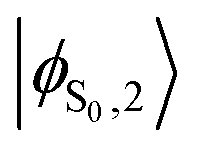
 state (black) and the ground state, 
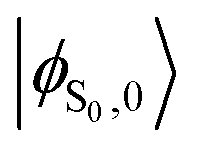
 (grey), in simulations of exciton dynamics in a system of *N* = 50 molecules with an exciton coupling strength of 〈*J*〉 = 70 meV outside (
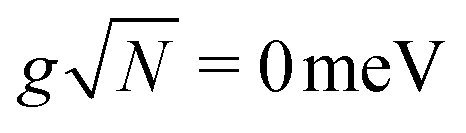
, a) and inside the cavity (
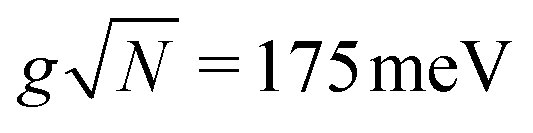
, b). Panel c: Comparison between the ground state populations in simulations of ordered (*σ*_E_ = 0, solid lines) and disordered (*σ*_E_ > 0, dashed lines) molecular ensembles inside the cavity (
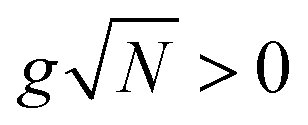
, red) and outside the cavity (
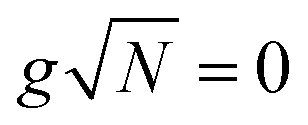
, blue). The data corresponding to the molecules with disorder (dashed lines) were replotted from panels a and b (grey lines). All lines are averages over 1000 realisations of the disordered Hamiltonian (eqn (2)). Note that the populations in panels a and b are plotted in logarithmic scale.

We first explore the influence of strong coupling on the EEA rate by performing simulations of polariton dynamics in a system of *N* = 50 TLMs with the exciton coupling strength 〈*J*〉 = 70 meV in a cavity without decay (*γ*_c_ = 0). In this case, the only deactivation channel is decay of S_*n*_-excitons, and the GS population thus serves as a measure of the efficiency of EEA. Fig. 2a shows the total populations of the two-S_1_-exciton states (
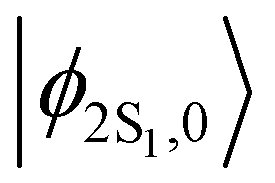
, cyan), the one-S_*n*_-exciton states (
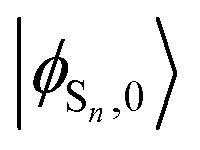
, green), and the ground state (
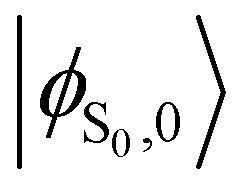
, grey) as a function of time in simulations outside the cavity (*i.e.*
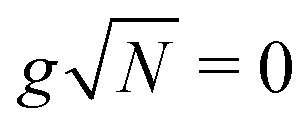
). After initial excitation into the 
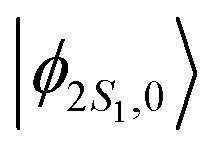
 state with one exciton created in molecule *i* = 13 and the other exciton created in molecule *j* = 37, *i.e.* at the maximum possible distance from each other, the total population of the states with two S_1_-excitons slowly decreases with the concomitant build-up of the states with one S_*n*_-exciton. The latter allows the population to decay to the ground state *via* deactivation terms −*iħγ*_v_, resulting in a growth of the population of this state. Noteworthy, the build-up of the 
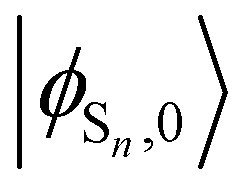
 and 
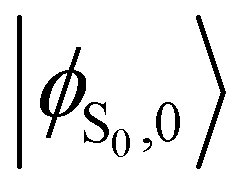
 populations does not follow the excitation immediately but starts after a delay of around 50 fs. This delay reflects the time the excitons need to travel before getting sufficiently near for the EEA to become efficient (Figure S6†).

Placing the TLMs inside the cavity (*i.e.*
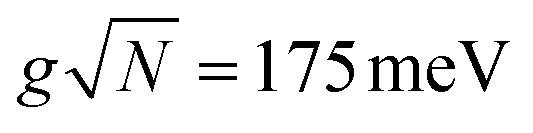
) opens up relaxation channels involving the hybrid light-matter states, resulting in a non-zero population of the 
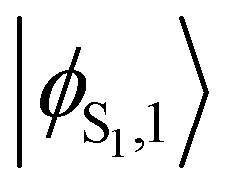
 states (Fig. 2b, purple) and the 
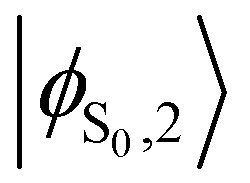
 state (black). Importantly, the GS population increases faster than outside the cavity (dashed lines in Fig. 2c), indicating an enhancement of the EEA rate due to strong coupling. In contrast to the EEA process outside the cavity, where disorder disrupts the excitation energy transfer and hence protects excitons against EEA, there is no such protection in the cavity. Through polariton formation, the cavity provides an additional transfer channel that is robust against disorder. Because a faster increase in the GS population inside the cavity is not observed in a system without excitation energy disorder (*i.e.* when *σ*_E_ = 0, solid lines in Fig. 2c), we attribute cavity-enhanced EEA in a disordered system to the ability of the cavity to provide additional connectivity between excitons *via* interaction with the common electromagnetic mode, which allows the excitons to partially overcome disorder.

We would like to emphasize that the enhancement of the EEA rate cannot be explained simply by Rabi oscillations arising from exciton-cavity coupling, which can occasionally populate the states with two neighbouring excitons. Indeed, as shown in Fig. S7a for a simulation with suppressed exciton coupling, *J* = 0, in which EEA can only arise from the interaction of excitons with the common cavity mode, Rabi oscillations make only a minor contribution to the GS population. Moreover, this contribution quickly decreases with the number of molecules (Fig. S7b and c). This is because the number of pairs of neighbouring excitons grows linearly with *N*, whereas the total number of available two-S_1_-exciton states grows as *N*(*N* − 1)/2 ∝ *N*^2^. Thus, the probability of targeting a state with two neighbouring excitons within one Rabi cycle is ∝ 1/*N*, which falls off rapidly with increasing *N*.

It is important to note that in the ideal case with no disorder, when *σ*_E_ = *σ*_*J*_ = *σ*_*V*_ = 0 meV, the GS population in the cavity is actually slightly smaller than in the case of bare molecules (solid lines in Fig. 2c), which is in line with the theoretical model of Siltanen *et al.*, in which an idealised molecule-cavity system was considered.^78^ This slight suppression of EEA is because part of the 
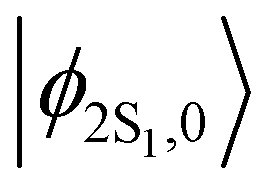
 population that is transferred to the 
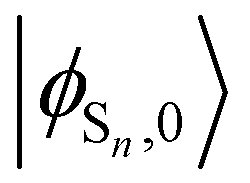
 states in the absence of strong coupling is transferred to the 
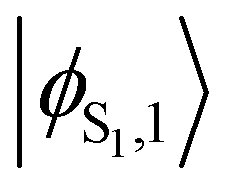
 and 
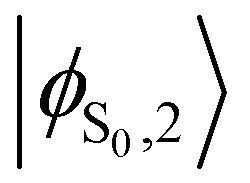
 states in the presence of strong coupling instead. Only in a disordered system does the enhancement of the EEA rate become possible.

Another curious observation that can be made from Fig. 2c is the difference in the character of the GS population build-up. In the absence of disorder (solid lines), the build-up is uneven and slows down after a rapid initial increase, whilst in the presence of disorder (dashed lines), nearly linear growth of the GS population is observed. The latter is because in addition to the disorder-induced localisation of the exciton population at the position of the two initially excited TLMs, an extended population uniformly distributed over the whole molecular chain is formed when TLMs are strongly coupled to the cavity (Fig. S8a and b). This results in a nonzero exciton coupling between any pair of neighbouring TLMs, which allows for EEA to continuously occur. Due to the linear increase in the disordered TLM-cavity system in contrast to the sub-linear increase in the system without disorder, the GS population in the former case eventually overtakes the GS population in the case of no disorder (≈1.4 ps for 〈*J*〉 = 70 meV, Fig. 2c). Interestingly, this occurs earlier in the strong light-matter coupling regime as the exciton coupling strength *J* increases (Fig. S8c). This can be explained by the fact that, while the GS population slightly increases with 〈*J*〉 in a disordered system, as we show below, the GS population decreases with 〈*J*〉 in the system without disorder. The latter is because an increase in exciton mobility reduces the time that excitons spend near each other, which leads to a decrease in the probability of exciton interaction. As a consequence, the GS population grows slower with increasing 〈*J*〉, in contrast to the disordered system (Fig. S8c).

**Fig. 3:**
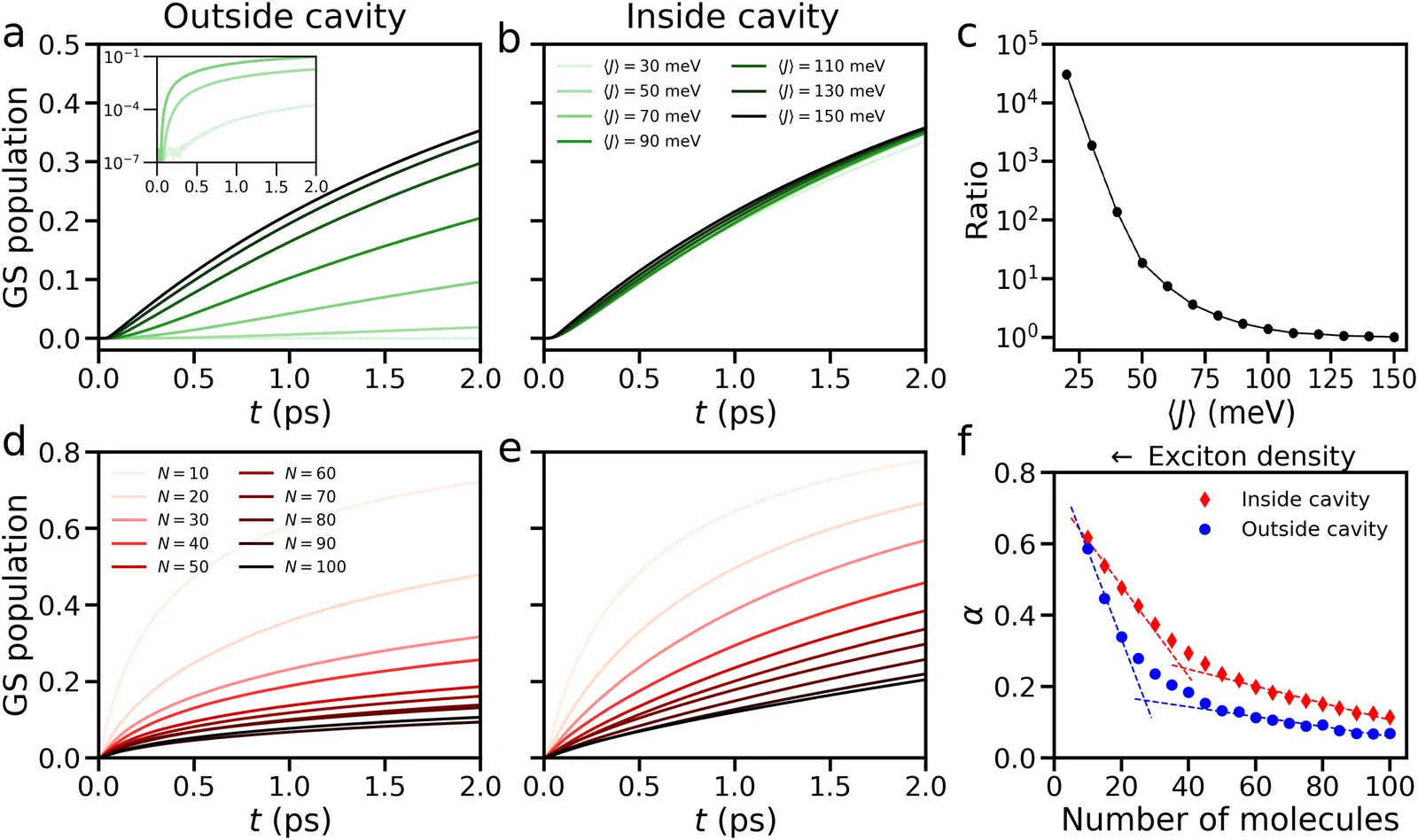
Panels a and b: Population of the ground state (GS), 
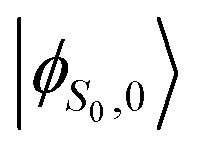
, in simulations with variable coupling strength, 〈*J*〉, and fixed number of molecules, *N* = 50, outside (a) and inside the cavity (b). The inset in panel a depicts the logarithmic scale plots of the GS population in simulations with 〈*J*〉 = 30 meV, 50 meV, and 70 meV. Panel c: Ratio between the GS populations inside and outside the cavity at the end of the simulation, *i.e., R* = *P*^inside^_GS_(*t*′)/*P*^outside^_GS_(*t*′) with *t*′ = 2 ps. Panels d and e: the GS population in simulations with variable number of molecules and fixed exciton coupling strength, 〈*J*〉 = 50 meV, outside (d) and inside the cavity (e). Panel f: Dependence of *α* on the number of molecules, *N*, inside (red diamonds) *vs.* outside the cavity (blue circles), with *α* extracted from fitting the GS population at different values of *N* to the function *P*_GS_(*t*) = *αt*^*β*^. The dashed lines are fits to a linear function at small and large numbers of molecules. The intersection of the lines indicates the onset of EEA, which occurs at a larger *N* (smaller exciton density) when molecules are strongly coupled to the cavity. All lines in panels a-b and d-e are averages over 1000 realisations of the disordered Hamiltonian (eqn (2)).

To further elaborate on the interplay between disorder, exciton mobility and polariton formation, we performed simulations at different exciton coupling strengths between 〈*J*〉 = 20 meV and 〈*J*〉 = 150 meV in a system of *N* = 50 TLMs, to gradually transition between the regimes of poor exciton mobility (〈*J*〉 ≪ *σ*_E_ with *σ*_E_ = 100 meV), moderate exciton mobility (〈*J*〉 ≤ *σ*_E_), and high exciton mobility (〈*J*〉 > *σ*_E_). The standard deviation of the exciton coupling distribution was kept *σ*_*J*_ = 10 meV for all values of 〈*J*〉. Fig. 3a demonstrates the time-dependence of the GS population for different values of 〈*J*〉 in the absence of strong coupling. When exciton coupling is much smaller than the disorder strength, 〈*J*〉 ≪ *σ*_E_, exciton propagation is suppressed due to Anderson localisation (Fig. S9a), resulting in negligible annihilation and therefore the GS population remains low (light line in the inset of Fig. 3a). Increasing 〈*J*〉 improves exciton transport (Fig. S9b,c) and increases the probability of exciton interaction. As a result, the GS population also increases with 〈*J*〉, reaching the value of ≈0.35 after two picoseconds in the case of the highest coupling strength of 〈*J*〉 = 150 meV.

In contrast, the GS population experiences very little dependence on the exciton coupling strength under strong light-matter coupling and in all cases is similar to the highest GS population outside the cavity at 〈*J*〉 = 150 meV (Fig. 3b). There, some of the probability amplitude of the total wave function (eqn (3)) associated with the exciton population, delocalises over the molecular chain (Fig. S8a and b), in which case the highest EEA rate can be expected due to continuous interaction *via* the coupling term *V* (eqn (2)). Therefore, the role of the cavity in enhancing the EEA rate is in expanding the exciton population over many molecules through their interaction with the common electromagnetic mode, which provides better connectivity between excitons and hence more efficient interaction. We note that with two excitons in a chain of *N* = 50 molecules considered in these simulations, the exciton density was 4%, which was close to an exciton density of 1% achieved in a study of EEA in J-aggregates of a cyanine dye.^39^ Therefore, we expect the picture described above to hold in realistic molecule-cavity systems.

By expanding the exciton population over many molecules, the cavity allows for efficient EEA even in the case of the smallest exciton coupling, in contrast to uncoupled TLMs, in which the exciton population is localised on the initially excited molecules due to disorder (Fig. S8b). To quantify the extent of exciton expansion, we calculated the escape probability of the excitonic wave packet as^79^7
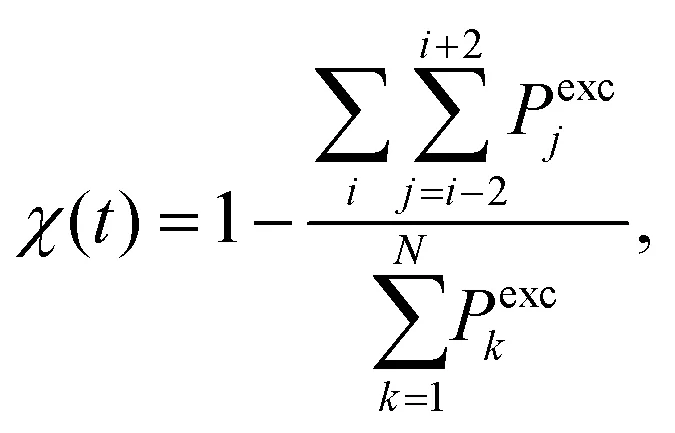
where *P*^exc^_*j*_ is the exciton probability density in TLM *j* (see Section 4.3 in the SI for details), and the terms in the numerator on the right-hand side of eqn (7) are summed over a narrow range around the initially excited TLMs, which we chose to be the TLMs with indices *i* = 13 and *i* = 37. An escape probability of zero corresponds to the situation where the entire population is in the initially excited TLMs, whereas *χ* = 1 indicates that no population remains in these TLMs. That is, the escape probability estimates the likelihood that the exciton wave packet will propagate beyond the initially excited spot. We found that the time-averaged escape probability increased gradually from *χ̄* = 0.09 to *χ̄* = 0.69 in the exciton coupling range from 〈*J*〉 = 20 meV to 〈*J*〉 = 150 meV in the absence of light-matter coupling (Fig. S10). In contrast, the escape probability increases only slightly between *χ̄* = 0.63 and *χ̄* = 0.71 within the same range of 〈*J*〉 in the strong coupling regime. The different character of the escape probability between 
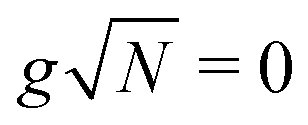
 and 
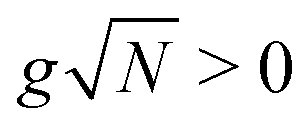
 is consistent with the difference in the GS population observed in Fig. 3a, b and further supports our claim that strong coupling provides delocalisation of excitons, which allows for efficient EEA even in systems with weak exciton interaction compared to the disorder strength.

In Fig. 3c we summarise the results presented in Fig. 3a, b by plotting the ratio between the GS populations in the presence and absence of strong light-matter coupling, *R* = *P*^inside^_GS_(*t*′)/*P*^outside^_GS_(*t*′), after *t*′ = 2 ps of simulation. At high exciton mobilities, 〈*J*〉 > *σ*_E_, excitons are already well connected, and the cavity has no effect on the EEA rate, resulting in *R* ≈ 1. As 〈*J*〉 decreases, exciton propagation outside the cavity becomes increasingly suppressed due to excitation energy disorder, and Anderson localisation of excitons takes place when 〈*J*〉 ≪ *σ*_E_. On the contrary, the suppression of exciton spreading is mitigated inside the cavity due to strong coupling, resulting in enhanced EEA rates and substantial increase in *R* (*e.g.*, *R* > 10^4^ at 〈*J*〉 = 20 meV and *σ*_E_ = 100 meV), indicating that strong coupling can significantly boost EEA in systems with low exciton mobility.

It is known that exciton annihilation manifests itself when the density of excitons reaches a certain threshold.^37^ Because the EEA rate is enhanced in a perfect cavity without decay, we can anticipate the onset of EEA to occur at a lower exciton density than in bare molecules. To test this, we performed simulations with a variable number of TLMs, ranging from *N* = 10 to *N* = 100, with a fixed exciton coupling strength of 〈*J*〉 = 50 meV. This range of the number of TLMs corresponds to an exciton density of 2–20%. This is higher than a critical density of 0.1%–1%, typical for organic films with a critical exciton concentration around 10^18^–10^19^ cm^−3^ and a molecular concentration of 10^21^ cm^−3^.^80–82^ Nevertheless, as we demonstrate below, it is still possible to qualitatively compare the threshold exciton densities in molecules inside and outside the cavity, even if the exact numbers may be overestimated. Because the length of the molecular chain was different in the simulations with variable *N*, the initial state was chosen to correspond to two S_1_-excitons located at random positions in each realisation of the disordered Hamiltonian (eqn (2)). Fig. 3d and e show the time-dependence of the GS population at different values of *N* outside and inside the cavity. In both cases, the GS population builds up faster when the number of TLMs is smaller – or, equivalently, exciton density is higher – since excitons become more likely to meet and interact.

The onset of annihilation is experimentally identified as a breakdown of the linear relationship between excitation fluence, which is proportional to the exciton density, and a measured observable, which is proportional to the excited-state population.^41^ Here, we imitate this approach by fitting the GS populations in Fig. 3d and e to the power-law function, *P*_GS_(*t*) = *αt*^*β*^, and plotting the extracted coefficient *α*, which quantifies the speed with which the GS population grows, as a function of the number of molecules in Fig. 3f. In the plot, we can distinguish two regimes, characterised by a linear increase in *α* with different slopes, shown as dashed lines. The threshold exciton density was then estimated from the intersection of these lines and found to be reduced under strong coupling with a lossless cavity (*γ*_c_ = 0). Specifically, the threshold exciton density *ρ* (number of molecules) was found to be *ρ* = 5.6% (*N*_th_ = 36) in the cavity and *ρ* = 7.7% (*N*_th_ = 26) in the absence of strong coupling. This result provides an additional argument to the statement that EEA can be enhanced in an ideal cavity. A qualitatively similar result was also found by plotting the *β* coefficient with respect to the number of molecules and extracting the threshold from the intersection of linear fits at small and large *N* (Fig. S11).

**Fig. 4:**
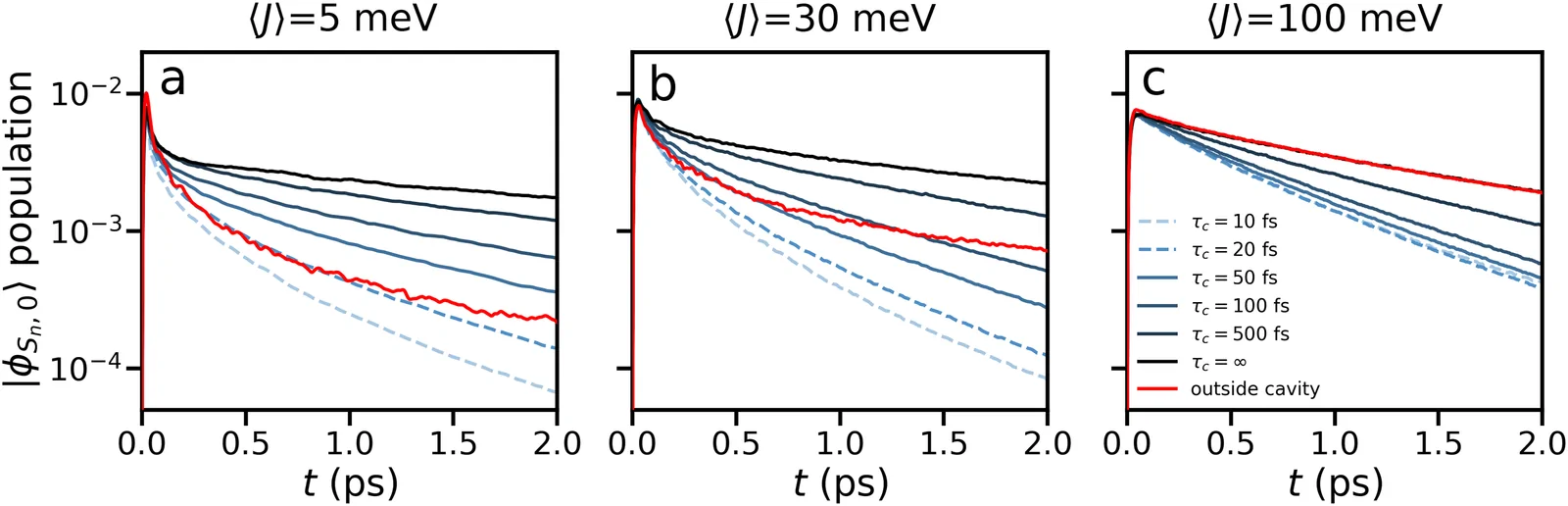
Total population of the 
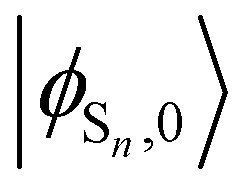
 states in simulations of exciton-polariton dynamics in a system of *N* = 50 three-level molecules with an exciton coupling strength of 〈*J*〉 = 5 meV (a), 〈*J*〉 = 30 meV (b), and 〈*J*〉 = 100 meV (c), and the cavity lifetime ranging between *τ*_c_ = 10 fs and *τ*_c_ = 500 fs, as well as in simulations in the ideal cavity with no decay (*γ*_c_ = 1/*τ*_c_ = 0) and outside the cavity. All lines are averages over 1000 realisations of the disordered Hamiltonian (eqn (2)).

So far, we have considered the TLMs in a perfect cavity without decay, *i.e. γ*_c_ = 0. However, real cavities have a limited lifetime, *τ*_c_, for the electromagnetic modes they support. To explore how the EEA rate changes with the quality of the cavity, we performed simulations in a variety of cavities with different lifetimes, corresponding to both the weak coupling regime (*τ*_c_ < 1/*Ω*_R_ = 21 fs) and the strong coupling regime (*τ*_c_ > 21 fs).^83^ Because in addition to the deactivation of the 
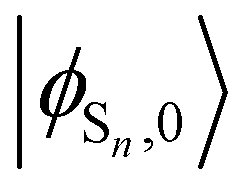
 states a decay channel described by the terms −*iħγ*_c_ and −2*iħγ*_c_ exists (eqn (2)), we cannot associate the GS population with EEA only. Instead, because the efficiency of EEA depends on the population of the 
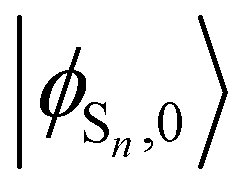
 states, we plot this population in Fig. 4 for systems with three different values of the exciton coupling strength, namely 〈*J*〉 = 5 meV, 30 meV, and 100 meV.

For the lowest intermolecular coupling strength (〈*J*〉 = 5 meV), the population of the S_*n*_-excitonic states under strong light-matter coupling exceeds the population outside the cavity (blue and red solid lines in Fig. 4a), which points to an enhancement of EEA as for the ideal cavity (Fig. 2 and 3). In contrast, under weak light–matter coupling conditions, the population of the S_*n*_-excitonic states is reduced compared to bare molecules (blue dashed lines in Fig. 4a), implying suppression of EEA. As the intermolecular exciton coupling increases to 〈*J*〉 = 30 meV, the effect of light-matter hybridisation on EEA diminishes, and the population of the 
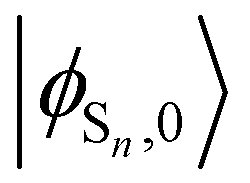
 states is lower inside the cavity than outside the cavity for cavities with *τ*_c_ ≤ 100 fs (Fig. 4b). Only in high Q-factor cavities with *τ*_c_ > 100 fs does the enhancement of the EEA rate remain possible. Upon increasing 〈*J*〉 even further, the outside-cavity population of states with one S_*n*_-exciton reaches the population in the ideal cavity with *τ*_c_ → ∞ (red and black lines in Fig. 4c). Because a finite cavity lifetime reduces the EEA efficiency, we conclude that placing materials with high intrinsic exciton mobility inside the cavity will suppress rather than enhance EEA.

Thus, the results of our simulations suggest that the overall effect of strong light–matter coupling on the rate of exciton annihilation depends on the relationship between material properties, in particular the exciton coupling strength *J* and the disorder strength *σ*_E_, and the cavity lifetime, *τ*_c_. In contrast, under weak coupling, the EEA rate appears to always be reduced due to cavity decay, at least in the case of a small number of molecules of *N* ≤ 100 as considered in this work.

Recently, Philipp *et al.* utilised Fermi's Golden rule to estimate the EEA rate in the strong coupling regime.^84^ The authors proposed that the cavity provides an additional source of exciton annihilation due to a resonant interaction between the S_1_ → S_*n*_ molecular transition and the cavity photons. To explore the relevance of this so-called exciton-photon annihilation in our system with molecular parameters characteristic of R6G, we incorporated coupling terms *g̃* between the 
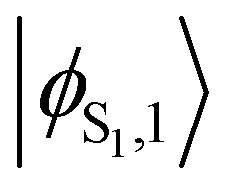
 and 
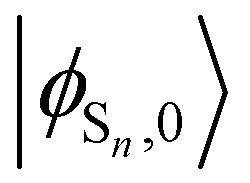
 states in the system's Hamiltonian (eqn (1)) and repeated the simulation of *N* = 50 TLMs with exciton coupling strength 〈*J*〉 = 50 meV under strong coupling without cavity decay (*γ*_c_ = 0). Because the TDM |***μ***_1→*n*_| corresponding to the S_1_ → S_*n*_ transition is 28 times smaller than the TDM |***μ***_0→1_| corresponding to the S_0_ → S_1_ transition in the optimised geometry of the R6G monomer, and six times smaller in the optimised geometry of the R6G H-dimer at the Time-Dependent Density Functional level of theory^85,86^ (Section 4.9 in the SI), the coupling strength responsible for exciton-photon annihilation was set to *g̃* = *g*/28 and *g̃* = *g*/6. In both situations, the resulting ground state population was found to be the same as in the absence of exciton-photon annihilation (Fig. S12), pointing to a negligible contribution of this effect to the total EEA rate in our system. We note, however, that in molecules with comparable oscillator strengths for the S_0_ → S_1_ and S_1_ → S_*n*_ transitions, *i.e.* when *g̃* ≈ *g*, exciton-photon annihilation is no longer negligible and should be taken into account when estimating the total rate of EEA (Fig. S12).

**Fig. 5:**
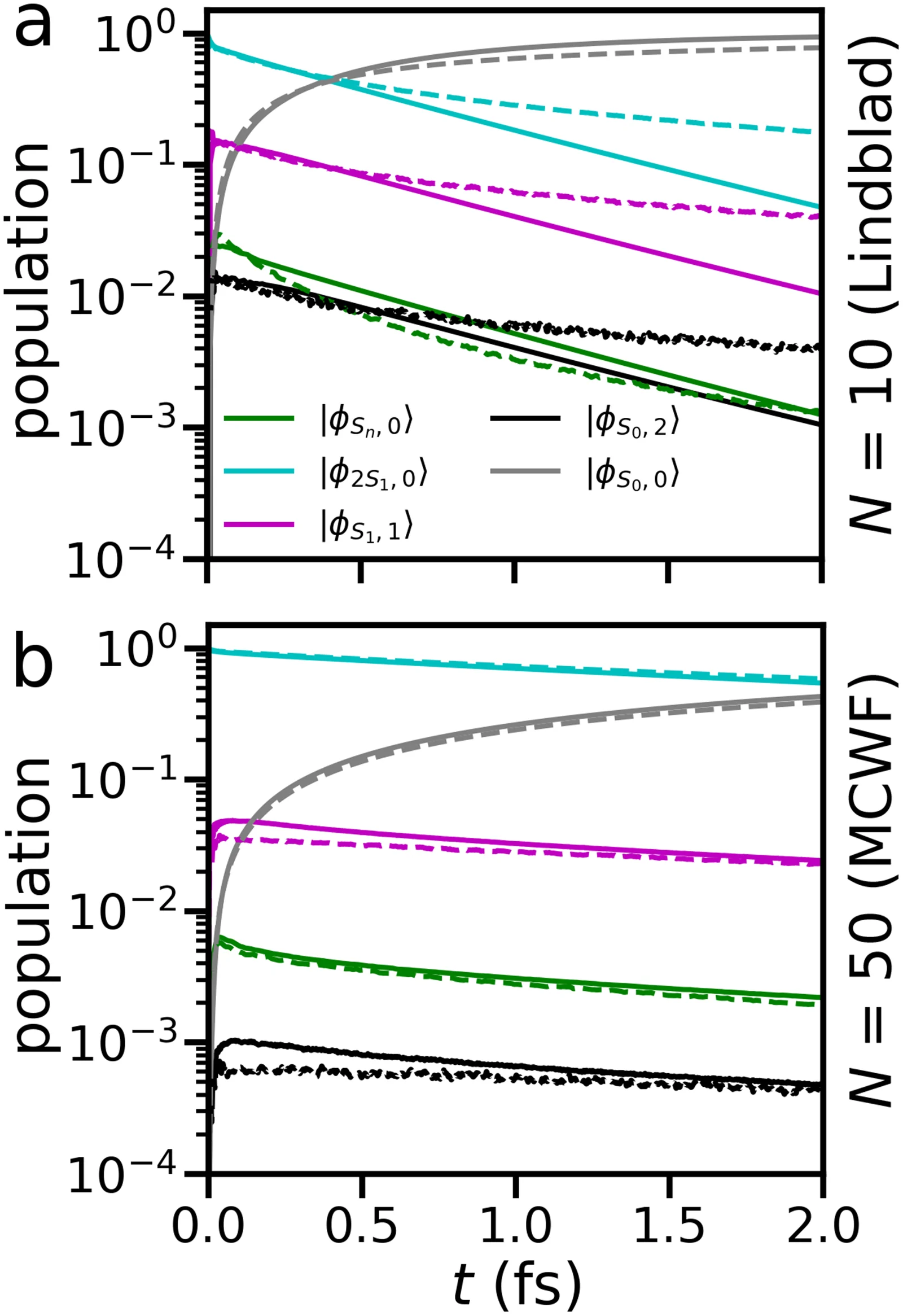
Total populations of all 
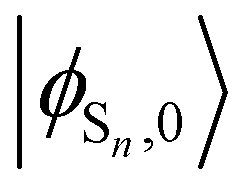
 states (green), 
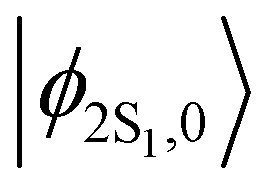
 states (cyan), 
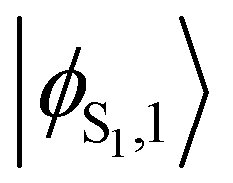
 states (purple), as well as of the 
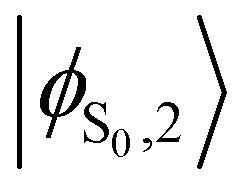
 state (black) and the ground state, 
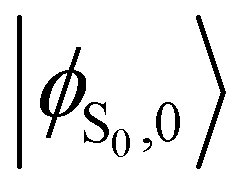
 (grey), in simulations of exciton-polariton dynamics in a system of *N* = 10 (panel a) and *N* = 50 (panel b) molecules with an exciton coupling strength of 〈*J*〉 = 50 meV and a collective light-matter coupling strength of 
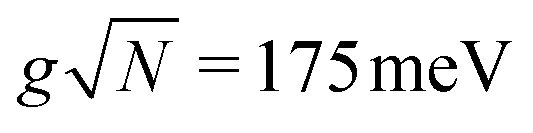
. The dashed lines correspond to the numerical integration of the Schrödinger equation without dephasing (eqn (4)), whereas the solid lines correspond to simulations with dephasing (*τ*_deph_ = 100 fs), performed by solving the Lindblad master equation (eqn (5)) in the case of *N* = 10 and by the Monte-Carlo wave function propagation in the case of *N* = 50. The lines corresponding to numerical integration of the Schrödinger equation are averages over 1000 realisations of the disordered Hamiltonian (eqn (2)), whereas the lines corresponding to the Lindblad and MCWF method are averages of 100 and 10 000 realisations, respectively.

Finally, we estimate the influence of pure molecular dephasing on the EEA rate arising from the weak interaction of molecules with the environment. Fig. 5 shows the populations of product states in the strong light-matter coupling regime in simulation of *N* = 10 TLMs obtained by directly solving the Lindblad master equation (eqn (5), panel a), and *N* = 50 TLMs obtained with the MCWF method (panel b), which provides reasonable agreement with the former method (Fig. S13 and S14). Compared to the isolated system, the initially excited states with two S_1_-excitons, *i.e.*, 
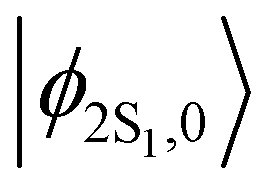
, exhibit a stronger depopulation towards the ground state in the system with dephasing. However, this effect decreases with increasing number of molecules, resulting in a smaller discrepancy in the total population of all kinds of product states. In particular, the deviation of the GS population after 2 ps of simulation with dephasing from simulation without dephasing reduces from ≈18% for *N* = 10 to ≈9% for *N* = 50. Importantly, this deviation does not change the conclusion that the EEA rate can either be increased or reduced depending on the magnitudes of the disorder strength and exciton coupling strength, as well as the cavity *Q*-factor. This is illustrated in Fig. S15 in the SI, which shows a gradual transition from suppression to enhancement of EEA with increasing cavity lifetime for a system with 〈*J*〉 = 30 meV, in line with Fig. 4b.

## Conclusions and outlook

In this work, we performed numerical simulations of exciton–exciton annihilation in the regime of strong light–matter coupling. Although the presence of the cavity does not directly influence exciton–exciton interactions,^87^*i.e.* coupling terms *J* and *V*, the EEA rate is modified by two effects related to the matter and photonic components of the resulting exciton-polaritons. These effects are, respectively, (i) cavity-enhanced exciton delocalisation, which allows for better connectivity between excitons with poor and moderate mobilities *via* interaction with the common cavity light modes, and (ii) decay into the ground state through the photonic component of the polaritons. Whereas the first effect increases the EEA rate, the second effect competes with exciton annihilation and hence reduces its efficiency. Thus, whether the strong light–matter coupling enhances or suppresses EEA depends on both the matter and cavity components. Matter properties that influence this trade-off are exciton coupling strength and the strength of excitation energy disorder, while for the cavity the *Q*-factor is the most important.

As was discussed in the Introduction, experiments reported both suppression and enhancement of EEA in organic and inorganic materials under strong coupling. Based on the results of our simulations, we suggest that these discrepancies might stem from different parameter regimes characterising the materials and cavities in the respective experiments, *i.e.* 〈*J*〉, *σ*_E_ and *τ*_c_. For example, in the experiment of Qureshi *et al.* in R6G,^41^ which showed suppression of EEA under strong coupling in contrast to the experiment conducted by Wu *et al.* with the same material,^40^ the formation of J- and H-aggregates was reported. This could have led to better exciton mobility, resulting in a diminished effect of strong coupling on exciton annihilation (Fig. 3) and, consequently, on the overall suppression of EEA due to cavity decay despite a higher *Q*-factor cavity compared to the experiments of Wu *et al*.

In addition, it was suggested that a high effective group velocity could bring some exciton–polaritons away from the excitation spot and locally depopulate the exciton density. While, in principle, such polariton-mediated transport can be described with our model,^66^ doing this for multiple excitons in the second excitation subspace becomes computationally prohibitive: the total number of states increases from *N*_st_ = 5151 in the case of a single mode and 100 TLMs to *N*_st_ = 20 100 in the case of 100 cavity modes and 100 TLMs. Alternatively, larger systems could potentially be addressed using the recently developed mean-field many-body Ehrenfest approach of Ghosh *et al.*,^62^ in which a single-permanent, Gross-Pitaevskii ansatz enables dynamics beyond the single-excitation subspace without explicitly constructing the full many-body Hilbert-space. Although the formulation of Ghosh *et al.*^62^ conserves excitation number and therefore does not itself describe EEA, an extension incorporating an explicit annihiliation channel could provide a computationally tractable route towards the lower exciton densities (0.1%–1%) typical of organic molecular films. Such an approach could furthermore include multiple cavity modes and thus address the influence of in-plane polariton transport and cavity dispersion on EEA.

For experimental validation of our finding that EEA can be both suppressed and enhanced depending on the relationship between the 〈*J*〉/*σ*_E_ ratio and the cavity lifetime, *τ*_*c*_, a good platform could be a material with moderate exciton mobility. By placing this material in cavities with increasing *Q*-factors, a gradual transition from suppression to enhancement of the EEA rate would confirm the results of our simulations with 〈*J*〉 = 30 meV (Fig. 4b).

In contrast to strong coupling, in the weak coupling regime we observed a suppression of the EEA rate regardless of the exciton mobility, which is due to the competing cavity decay channel. This result appears to contradict the experiment of Wu *et al.*, who observed that the lifetime of transient transmission deviated from a constant value at lower pump intensities for both strong and weak coupling compared to bare films of LH2 complexes and R6G dye.^40^ Although the authors explained this result by enhanced EEA, an alternative explanation based on an increase in the intensity of absorbed light at weak coupling is also possible. This necessitates further experimental research to elucidate whether the enhancement of the EEA rate is possible in the weak coupling regime.

Regarding achieving BEC and lasing of polaritons, the best option would be to choose a material that does not undergo EEA at all yet can strongly couple to cavity light modes.^39^ However, given that polariton lasers require relatively high external pumping, at which EEA occurs in most molecules, it could be very difficult to find such a material. Nevertheless, by carefully designing organic semiconductors with favourable molecular packing^88,89^ or quantum phase relationship between exciton wave functions,^90,91^ or by choosing materials with a mismatch between the energies of the S_1_ and S_*n*_ states, the effect of EEA can be mitigated. Moreover, such materials with low exciton annihilation propensity can be combined with cavity structures that allow for polariton lifetimes to be longer than the rates of polariton–phonon relaxation.^92,93^ Eventually, this may lead to efficient polariton relaxation to the bottom of the LP branch, as is required for the operation of polariton lasers.

## Author contributions

I. S.: conceptualization, data curation, formal analysis, funding acquisition, investigation, methodology, validation, visualization, writing – original draft. B. H.: data curation, investigation, writing – review & editing. J. B.: funding acquisition, methodology, supervision, writing – review & editing. G. G.: conceptualization, methodology, supervision, writing – review & editing.

## Conflicts of interest

The authors declare no conflict of interest.

## Supplementary Material

CP-028-D6CP02032A-s001

## Data Availability

The simulation data, including all Python scripts required to perform and analyse the calculations, are available from the authors upon reasonable request. Supplementary information (SI) contains the details of molecular dynamics simulations, the description and benchmark of the Lindblad master equation and Monte-Carlo wave function methods, as well as additional figures, which support the information presented in the main text. See DOI: https://doi.org/10.1039/d6cp02032a.
