# Supplementary material for: Cavity-modified exciton–exciton annihilation in disordered molecular systems
Source: Phys Chem Chem Phys. 2026 Aug 21;28(35):21431–43. doi: 10.1039/d6cp02032a (PMC13580608; doi:10.1039/d6cp02032a)
Supplement: Supplementary file 1 [file CP-028-D6CP02032A-s001.pdf]

Supplementary Information (SI) for Physical Chemistry Chemical Physics.
This journal is © the Owner Societies 2026

Supporting information for Cavity-modified exciton-exciton annihilation in disordered molecular systems

Ilia Sokolovskii,[*,†] Ben S. Humphries,[†] Jochen Blumberger,[†] and Gerrit Groenhof[‡]

†*Department of Physics and Astronomy and Thomas Young Centre, University College London, Gower Street, London WC1E 6BT, United Kingdom*

‡*Nanoscience Center and Department of Chemistry, University of Jyväskylä, P.O. Box 35, 40014 Jyväskylä, Finland*

E-mail: i.sokolovskii@ucl.ac.uk

Contents

1 Modified Dicke Hamiltonian in matrix form **3**

2 Rabi splitting in the second excitation subspace **5**

3 Molecular Dynamics simulations of Rhodamine 6G H-dimer **8**

4 Additional results **14**

4.1 Convergence of dynamics with respect to time step 14

4.2 Effect of cavity detuning on population dynamics 15

4.3 Short-time exciton dynamics 16

4.4 Contribution of Rabi oscillations to the ground state population 17

4.5 Comparison between molecule-cavity systems with and without disorder 18

4.6 Exciton propagation in systems with different exciton mobility 19

4.7 Exciton escape probability 20

4.8 Estimation of the threshold density for the onset of EEA 21

4.9 Contribution of exciton-photon annihilation to the total EEA rate 22

5 Effect of molecular dephasing on the EEA rate **24**

5.1 Jaynes-Cummings model 25

5.2 Simulation of EEA with the Lindblad master equation 26

5.2.1 Computational scaling of direct Lindblad simulations 26

5.2.2 Implications for EEA simulations 27

5.2.3 Projected Runtimes for Larger Systems 27

5.2.4 Full 2.0 ps Runtime Estimates 28

5.2.5 Memory requirement of direct Lindblad solution 29

5.3 Simulation of EEA with the Monte-Carlo wave function propagation 31

References **36**

1 Modified Dicke Hamiltonian in matrix form

Equation S1 represents the matrix form of the modified Dicke Hamiltonian, introduced in Equation 1 in the main text, for the case of $N = 3$ three-level molecules (TLMs). Here, the diagonal elements highlighted in blue, represent the molecular and/or photonic excitations in the zeroth (ground state with energy $E_g = 0$), first (excitation of either a S_1-exciton with energy $E_{S_1,i}$ or a cavity photon with energy E_c), and second excitation subspace (excitation of either a S_n-exciton with energy $E_{S_n,i}$, or two S_1-excitons with energies $E_{S_1,i}$ and $E_{S_1,j}$ simultaneously, or one S_1-exciton and one cavity photon simultaneously, or two cavity photons with total energy $2E_c$). The red elements, J, describe transfers of S_1-excitons between different TLMs, whereas the green elements, V, are responsible for exciton-exciton annihilation (EEA). Finally, the elements highlighted in cyan, describe light-matter couplings within the first excitation subspace ($\hbar g$), within the second excitation subspace ($\hbar g$ and $\sqrt{2}\hbar g$), as well as between the zeroth and the second excitation subspaces ($\hbar g$). Within the Rotating wave approximation, the counter-rotating terms (*i.e.* $\hbar g$ terms in the first row and the first column) that simultaneously create or destroy an exciton and a photon, are neglected, and the block of the Hamiltonian corresponding to the second excitation subspace becomes decoupled from the block corresponding to the zeroth excitation subspace. Therefore, in the current work, we reduce the Hamiltonian's size and consider only the block corresponding to the second excitation subspace.

$$
\hat{\mathrm{H}} = \begin{pmatrix}
E_g & 0 & 0 & 0 & 0 & 0 & 0 & 0 & 0 & 0 & 0 & \hbar g & \hbar g & \hbar g & 0 \\
0 & E_{S_1,1} & J & J & \hbar g & 0 & 0 & 0 & 0 & 0 & 0 & 0 & 0 & 0 & 0 \\
0 & J & E_{S_1,2} & J & \hbar g & 0 & 0 & 0 & 0 & 0 & 0 & 0 & 0 & 0 & 0 \\
0 & J & J & E_{S_1,3} & \hbar g & 0 & 0 & 0 & 0 & 0 & 0 & 0 & 0 & 0 & 0 \\
0 & \hbar g & \hbar g & \hbar g & E_c & 0 & 0 & 0 & 0 & 0 & 0 & 0 & 0 & 0 & 0 \\
0 & 0 & 0 & 0 & 0 & E_{S_n,1} & 0 & 0 & V & V & 0 & 0 & 0 & 0 & 0 \\
0 & 0 & 0 & 0 & 0 & 0 & E_{S_n,2} & 0 & V & 0 & V & 0 & 0 & 0 & 0 \\
0 & 0 & 0 & 0 & 0 & 0 & 0 & E_{S_n,3} & 0 & V & V & 0 & 0 & 0 & 0 \\
0 & 0 & 0 & 0 & 0 & V & V & 0 & E_{S_1,1}+E_{S_1,2} & J & J & \hbar g & \hbar g & 0 & 0 \\
0 & 0 & 0 & 0 & 0 & V & 0 & V & J & E_{S_1,1}+E_{S_1,3} & J & \hbar g & 0 & \hbar g & 0 \\
0 & 0 & 0 & 0 & 0 & 0 & V & V & J & J & E_{S_1,2}+E_{S_1,3} & 0 & \hbar g & \hbar g & 0 \\
\hbar g & 0 & 0 & 0 & 0 & 0 & 0 & 0 & \hbar g & \hbar g & 0 & E_{S_1,1}+E_c & J & J & \sqrt{2}\hbar g \\
\hbar g & 0 & 0 & 0 & 0 & 0 & 0 & 0 & \hbar g & 0 & \hbar g & J & E_{S_1,2}+E_c & J & \sqrt{2}\hbar g \\
\hbar g & 0 & 0 & 0 & 0 & 0 & 0 & 0 & 0 & \hbar g & \hbar g & J & J & E_{S_1,3}+E_c & \sqrt{2}\hbar g \\
0 & 0 & 0 & 0 & 0 & 0 & 0 & 0 & 0 & 0 & 0 & \sqrt{2}\hbar g & \sqrt{2}\hbar g & \sqrt{2}\hbar g & 2E_c
\end{pmatrix} \tag{S1}
$$

2 Rabi splitting in the second excitation subspace

In the absence of the excitonic and EEA coupling, the Hamiltonian in the second-excitation subspace can be written as

$$\hat{\mathrm{H}} = \begin{pmatrix} E_{S_n,1} & 0 & 0 & 0 & 0 & 0 & 0 & 0 & 0 & 0 \\ 0 & E_{S_n,2} & 0 & 0 & 0 & 0 & 0 & 0 & 0 & 0 \\ 0 & 0 & E_{S_n,3} & 0 & 0 & 0 & 0 & 0 & 0 & 0 \\ 0 & 0 & 0 & E_{S_1,1}+E_{S_1,2} & 0 & 0 & \hbar g & \hbar g & 0 & 0 \\ 0 & 0 & 0 & 0 & E_{S_1,1}+E_{S_1,3} & 0 & \hbar g & 0 & \hbar g & 0 \\ 0 & 0 & 0 & 0 & 0 & E_{S_1,2}+E_{S_1,3} & 0 & \hbar g & \hbar g & 0 \\ 0 & 0 & 0 & \hbar g & \hbar g & 0 & E_{S_1,1}+E_c & 0 & 0 & \sqrt{2}\hbar g \\ 0 & 0 & 0 & \hbar g & 0 & \hbar g & 0 & E_{S_1,2}+E_c & 0 & \sqrt{2}\hbar g \\ 0 & 0 & 0 & 0 & \hbar g & \hbar g & 0 & 0 & E_{S_1,3}+E_c & \sqrt{2}\hbar g \\ 0 & 0 & 0 & 0 & 0 & 0 & \sqrt{2}\hbar g & \sqrt{2}\hbar g & \sqrt{2}\hbar g & 2E_c \end{pmatrix} \tag{S2}$$

where, for the sake of space, we assumed that the number of TLMs was equal to three ($N = 3$).

The red block of the Hamiltonian in Equation S2 is an arrowhead matrix, representing the coupling between the states with the excitation of a molecule into its first excited state and of a cavity Fock state ($|\phi_{S_1,1}\rangle$), and the state with two excitations of the cavity photon ($|\phi_{S_0,2}\rangle$), hence the off-diagonal elements are multiplited by $\langle 2|\langle S_0|\hat{\sigma}^-\hat{a}^\dagger|S_1\rangle|1\rangle = \sqrt{2}$.[1] In the absence of disorder and under the resonant condition (*i.e.* $E_{S_1,j} = E_0\ \forall j$ and $E_c = E_0$), the diagonalisation of this matrix yields two polaritonic states with energies $2E_0 \pm \hbar g\sqrt{2N}$ and $N-1$ dark states with energy $2E_0$. Because the dark states lack a contribution from the cavity photons, the Hamiltonian in Equation S2 can be represented in terms of the coupling between the states with two molecules excited into their first excited state ($|\phi_{2S_1,0}\rangle$; the blue block of the Hamiltonian) and the two polaritonic states:

$$\hat{\mathrm{H}} = \begin{pmatrix} E_{S_n,1} & 0 & 0 & 0 & 0 & 0 & 0 & 0 \\ 0 & E_{S_n,2} & 0 & 0 & 0 & 0 & 0 & 0 \\ 0 & 0 & E_{S_n,3} & 0 & 0 & 0 & 0 & 0 \\ 0 & 0 & 0 & E_{S_1,1}+E_{S_1,2} & 0 & 0 & \sqrt{2/N}\hbar g & \sqrt{2/N}\hbar g \\ 0 & 0 & 0 & 0 & E_{S_1,1}+E_{S_1,3} & 0 & \sqrt{2/N}\hbar g & \sqrt{2/N}\hbar g \\ 0 & 0 & 0 & 0 & 0 & E_{S_1,2}+E_{S_1,3} & \sqrt{2/N}\hbar g & \sqrt{2/N}\hbar g \\ 0 & 0 & 0 & \sqrt{2/N}\hbar g & \sqrt{2/N}\hbar g & \sqrt{2/N}\hbar g & 2E_c - g\sqrt{2N} & 0 \\ 0 & 0 & 0 & \sqrt{2/N}\hbar g & \sqrt{2/N}\hbar g & \sqrt{2/N}\hbar g & 0 & 2E_c + g\sqrt{2N} \end{pmatrix} \tag{S3}$$

where the coupling elements are multiplied by $\sqrt{2/N}$ to account for the fact that each $|\phi_{2S_1,0}\rangle$ state interacts with only two $|\phi_{S_1,1}\rangle$ states out of N such states.

The splitting between the highest and lowest energy states of the Hamiltonian in Equation S3 can be defined in a similar way to the general expression for the Rabi splitting in the first-excitation subspace,[2] $\Omega_{\mathrm{R}}^{(1)} = 2\sqrt{g^2 N + (\omega_0 - \omega_c)^2/4}$ with $\omega_0 = E_0/\hbar$ and $\omega_c = E_c/\hbar$, resulting in the following expression:

$$\begin{aligned} \Omega_{\mathrm{R}}^{(2)} &= 2\sqrt{2\left(\sqrt{\tfrac{2}{N}}g\right)^2 N_{\text{comb}} + (2\Delta E/\hbar)^2/4} = \\ & 2\sqrt{4\tfrac{g^2}{N}\tfrac{(N-1)N}{2} + (2g\sqrt{2N})^2/4} = \\ & 2g\sqrt{2\,(N-1) + 2N} = 2g\sqrt{4N-2} \end{aligned} \tag{S4}$$

with $2\Delta E$ being the sum of the energy shifts between the $|\phi_{2S_1,0}\rangle$ states of energy $2E_0$ and the polaritonic states of energy $2E_0 \pm \hbar g\sqrt{2N}$, *i.e.* $2\Delta E = 2\hbar g\sqrt{2N}$. In addition, $N_{\text{comb}} = C_N^2$ is the number of combinations of two different molecules excited into their first excited state, *i.e.* the number of diagonal elements in the blue-coloured block of the Hamiltonian in Equation S3. Finally, the first term under the square root is multiplied by two to account for the interaction with two polaritonic states.

As an example, Figure S1 depicts the eigenvalues of the Hamiltonian in Equation S2 for a system of $N = 7$ isoenergetic molecules (E_0) resonantly coupled to a cavity mode with the collective coupling strength $g\sqrt{N} = 100$ meV in the absence of the coupling terms responsible for exciton hopping and EEA, *i.e.* $J = V = 0$ meV. There, one can distinguish three types of polaritonic states with different contributions from the cavity, P_{phot}: *i)* multi-polariton states (yellow) with the photonic content $P_{\text{phot}} \approx 0.5$ and the energy splitting $\hbar\Omega_{\text{MP}} = 2\hbar g\sqrt{4N-2} = 385.5$ meV; *ii)* dark polariton states (magenta) with the photonic content $P_{\text{phot}} \approx 0.25$, energies $E_{\text{DP}} = E_0 \pm \hbar g\sqrt{N-2}$ and E_0 and the highest splitting $\hbar\Omega_{\text{DP}} = 2\hbar g\sqrt{N-2} = 169.0$ meV; and *iii)* dark states with the photonic content $P_{\text{phot}} \approx 0$ and energy $E_{\text{DS}} = E_0$.

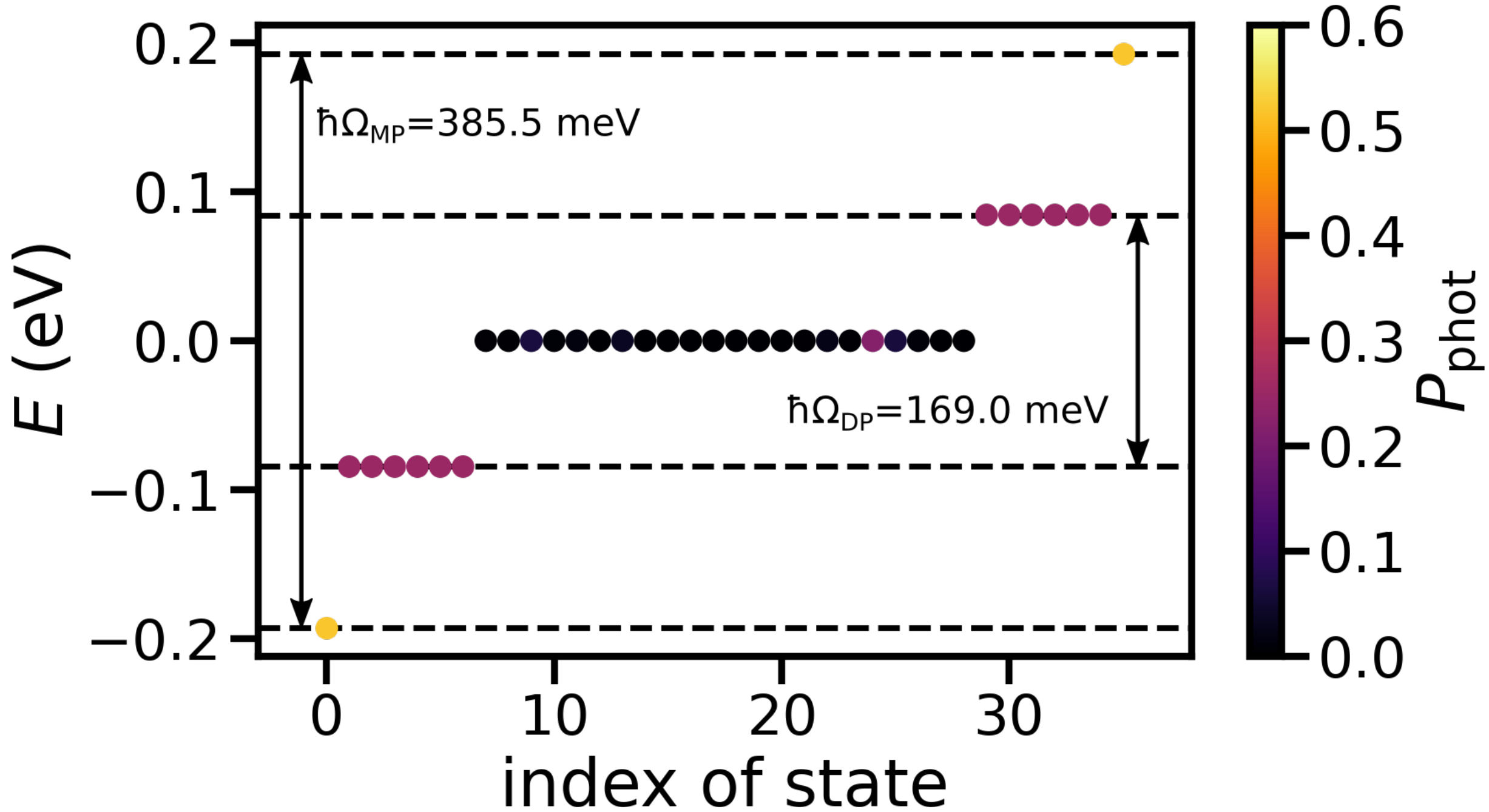

Figure S1: Eigenvalues of the light-matter Hamiltonian in Equation S2 for $N = 7$ molecules and $g\sqrt{N} = 100$ meV.

3 Molecular Dynamics simulations of Rhodamine 6G H-dimer

In two experiments on cavity-modified exciton-exciton annihilation (EEA), Rhodamine 6G (R6G, Figure S2**a**) fluorophore was strongly coupled to the cavity.[3,4] It is known that this molecule tends to aggregation at high concentrations with the formation of both J- and H-dimers, as well as trimers.[5,6] Therefore, to estimate the maximum possible coupling strength between excitons in R6G, we considered the R6G H-dimer, since its formation is energetically favourable over the J-dimer formation.[7]

First, we performed geometry optimisation of the H-dimer in vacuum at the Density Functional level of Theory[8] (DFT) using the range-separated hybrid ωB97X functional[9] with the D4 dispersion correction[10] and the def2-TZVPP basis set.[11] Figure S2**b** shows the optimised structure of the dimer, in which the magenta arrows denote the transition dipole moment (TDM) vectors of the monomers for excitation from the ground state (GS), S_0, to the first excited state, S_1 ($\boldsymbol{\mu}_{0\to 1}$), calculated with the Time-Dependent DFT.[12] The vectors are collinear, which is characteristic of H-dimers. The first excited state of the dimer has a negligible oscillator strength of $f = 0.0007$, while the second excited state is optically bright with oscillator strength $f = 1.7666$, which also indicates that the dimer is of the H-type. The dipole-dipole interaction between the S_1-excitons allows for exciton hops between adjacent molecules. The corresponding coupling term can be calculated using the transition charge approach:[13]

$$J_{i,k}^{\text{TrESP}} = \sum_{A\in i}\sum_{B\in k} \frac{q_{T,A}^{0\to 1} q_{T,B}^{0\to 1}}{|\mathbf{r}_A - \mathbf{r}_B|}, \tag{S5}$$

where A and B are atoms belonging to molecules i and k, respectively; $q_{T,A}^{0\to 1}$ and $q_{T,B}^{0\to 1}$ are transition electrostatic potential (TrESP) charges for the $S_0 \to S_1$ excitation, and $\mathbf{r}_{A/B}$ are atomic positions. For the optimised H-dimer, the transition charge approach gives a coupling strength of $J_{\text{opt}} =$ 210 meV.

The orange arrows in Figure S2**b** denote the TDM vectors of the monomers for excitation from the S_1-state to the S_{20}-state ($\boldsymbol{\mu}_{1\to 20}$). The energy of state S_{20} is nearly twice as high as the energy of the S_1-state, and the $S_1 \to S_{20}$ transition has a much greater oscillator strength compared to the

transitions to other states with energies close to $2E_{S_1}$ (Table S1). Therefore, the S_{20}-state is expected to make the largest contribution to EEA. The coupling term responsible for EEA is calculated using the transition charge approach as well:

$$V_{i,k}^{\text{TrESP}} = \frac{1}{2}\left[\sum_{A\in i}\sum_{B\in k}\frac{q_{T,A}^{0\to 1}q_{T,B}^{1\to n}}{|\mathbf{r}_A - \mathbf{r}_B|} + \sum_{A\in i}\sum_{B\in k}\frac{q_{T,A}^{1\to n}q_{T,B}^{0\to 1}}{|\mathbf{r}_A - \mathbf{r}_B|}\right], \quad \text{(S6)}$$

where $q_{T,A/B}^{1\to n}$ is the TrESP charge for excitation from the first excited state to the n-th excited state with energy $E_{S_n} \approx 2E_{S_1}$. The calculated values of the coupling term V for $n = 19, 20, 21, 22$ in the optimised geometry of the R6G H-dimer are presented in Table S2.

Additionally, we optimised the geometry of the R6G monomer at the same level of theory (DFT/ωB97X//def2-TZVPP) and analysed the excited states. The oscillator strength of the transition to the first excited state with energy $E_{S_1} = 3.394$ eV ($f = 1.108$) was estimated to be ≈ 4 and ≈ 96 times higher than the oscillator strength of the transition to the 18th and 19th excited states with energies $E_{S_{18}} = 6.580$ meV ($f = 0.311$) and $E_{S_{19}} = 6.735$ meV ($f = 0.012$), respectively. Therefore, we assumed that the excitation of the latter states was unable to enter strong coupling with the second cavity mode and hence did not include the corresponding coupling term in the system's Hamiltonian (Equation 1 in the main text), nor did we consider the exciton coupling between the S_n-excitons.

To explore how the coupling terms J and V are affected by thermal molecular motions, we performed molecular dynamics (MD) simulations of the R6G H-dimer in water. The parameters of the model and the simulation protocol were taken from the article by Doveiko *et al.*[14] Briefly,

Table S1: Energies and oscillator strengths of transitions between different states of the monomers in the geometry optimised H-dimer of Rhodamine 6G.

Transition	$S_0 \to S_1$	$S_1 \to S_{19}$	$S_1 \to S_{20}$	$S_1 \to S_{21}$	$S_1 \to S_{22}$
Monomer 1					
Energy, eV	3.428	3.391	3.494	3.583	3.626
Osc. strength	1.123	0.003	0.046	0.001	0.022
Monomer 2					
Energy, eV	3.427	3.399	3.504	3.579	3.619
Osc. strength	1.128	0.006	0.200	0.018	0.0004

the simulation box with 27442 atoms was initially minimised with fixed atoms of R6G and then equilibrated for 10 ps with a time step 1 fs. Temperature and pressure were maintained at 300 K and 1 bar with a Langevin barostat and thermostat in the NPT ensemble. The system was then minimised again, but without imposing constraints on the R6G atoms, with the subsequent heating to 300 K for 30 ps and equilibration for 270 ps. Finally, the MD production was conducted for 2.5 ns in the NVT ensemble using a Langevin thermostat with a damping constant of 5 ps^{-1}. In all simulations, the CHARMM36 force field was used to model the interactions between R6G molecules and water molecules.[15]

Figure S2**c** shows the time-evolution of the coupling terms J and V during the MD production, which were calculated using Equations S5 and S6 with frozen TrESP charges corresponding to the geometry optimised H-dimer. It was previously shown that using frozen transition charges is a decent approximation, which provides a satisfactory estimation of excitonic couplings.[16] Figure S2**d** depicts histograms of the coupling terms for 2500 frames extracted from the 2.5 ns trajectory. These histograms were fitted with Gaussian functions $A \exp\left[-\frac{\left(J(t)-\langle J\rangle\right)^2}{2\sigma_J^2}\right]$ and $B \exp\left[-\frac{\left(V(t)-\langle V\rangle\right)^2}{2\sigma_V^2}\right]$, from which the average couplings, $\langle J\rangle$ and $\langle V\rangle$, and their fluctuations, σ_J and σ_V, were extracted and presented in Table S2.

Simulations of polariton dynamics were performed over a wide range of exciton couplings $\langle J\rangle$, and the value $\langle J\rangle_{\text{MD}} \approx 150$ meV obtained from the MD simulation, was considered as a high-concentration limit, at which R6G molecules undergo dimerisation. The average EEA coupling terms, $\langle V\rangle_{\text{MD}}$, extracted from MD simulations are close to zero for all transitions except for the $S_1 \to S_{20}$ transition, for which $\langle V\rangle_{\text{MD}}^{S_1\to S_{20}} \approx 20$ meV (Table S2). This value was used in our

Table S2: Exciton coupling J and couplings V for the dipole interaction between the $S_0 \to S_1$ transition and the $S_1 \to S_n$ transition with $n = 19, 20, 21,$ and 22. The reported values are the coupling terms in the optimised geometry of the Rhodamine 6G H-dimer (second row), coupling terms averaged over a 2.5 ns long molecular dynamics trajectory (third row), as well as their standard deviations (fourth row). All values are in meV.

Coupling term	J	$V_{S_1\to S_{19}}$	$V_{S_1\to S_{20}}$	$V_{S_1\to S_{21}}$	$V_{S_1\to S_{22}}$
In optimised geometry	210	1.5	9.3	0.3	1.3
Average from MD	147	1.5	22	4.0	0.6
Standard deviation	26	2.4	146	17	4.3

simulations of polariton dynamics. We also chose the standard deviations of the coupling terms σ_J and σ_V both to be 10 meV.

Because coupling terms V can fluctuate significantly due to thermal motions of the molecules, especially in the case of the transition with the highest oscillator strength (green histogram in Figure S2**d**), we performed additional simulations of polariton dynamics with enhanced EEA coupling and its fluctuation, namely $\langle V \rangle = 50$ meV and $\sigma_V = 50$ meV. The results of this simulation show a gradual change from suppression to enhancement of the EEA rate with an increase in the cavity lifetime (Figure S3) as in the case when $\langle V \rangle = 20$ meV and $\sigma_V = 10$ meV, which indicates that the conclusions made in the work do not depend on the value of the EEA coupling strength.

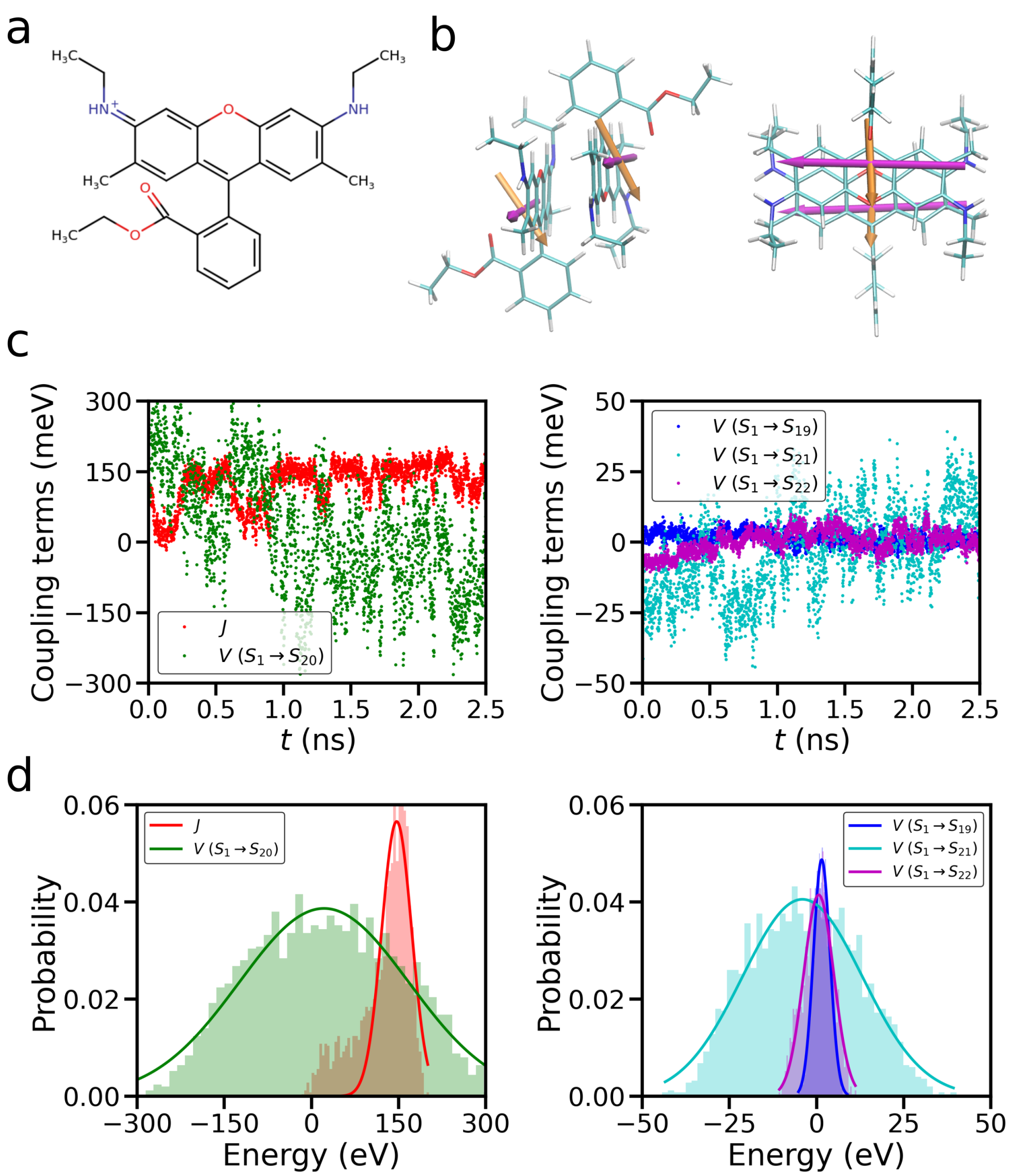

Figure S2: Panel **a**: Chemical structure of Rhodamine 6G (R6G). Panel **b**: Structure of the H-dimer of R6G after geometry optimisation at the TD-DFT/ωB97X-D4//def-TZVPP level of theory. Colors indicate the following atoms: green - carbon, white - hydrogen, red - oxygen, blue - nitrogen. The purple arrows are transition dipole moment (TDM) vectors corresponding to the $S_0 \rightarrow S_1$ excitation of the monomers ($\boldsymbol{\mu}_{0\rightarrow 1}$), and the orange arrows are TDM vectors corresponding to the $S_1 \rightarrow S_n$ excitation of the monomers ($\boldsymbol{\mu}_{1\rightarrow n}$) with energy of the S_n-state given by $E_{S_n} \approx 2E_{S_1}$. Panel **c**: Fluctuation of the exciton coupling J and the dipole-dipole coupling V between $\boldsymbol{\mu}_{0\rightarrow 1}$ and $\boldsymbol{\mu}_{1\rightarrow n}$ with $n = 19, 20, 21, 22$, extracted from molecular dynamics simulations. Panel **d**: Corresponding histograms fitted with a Gaussian function.

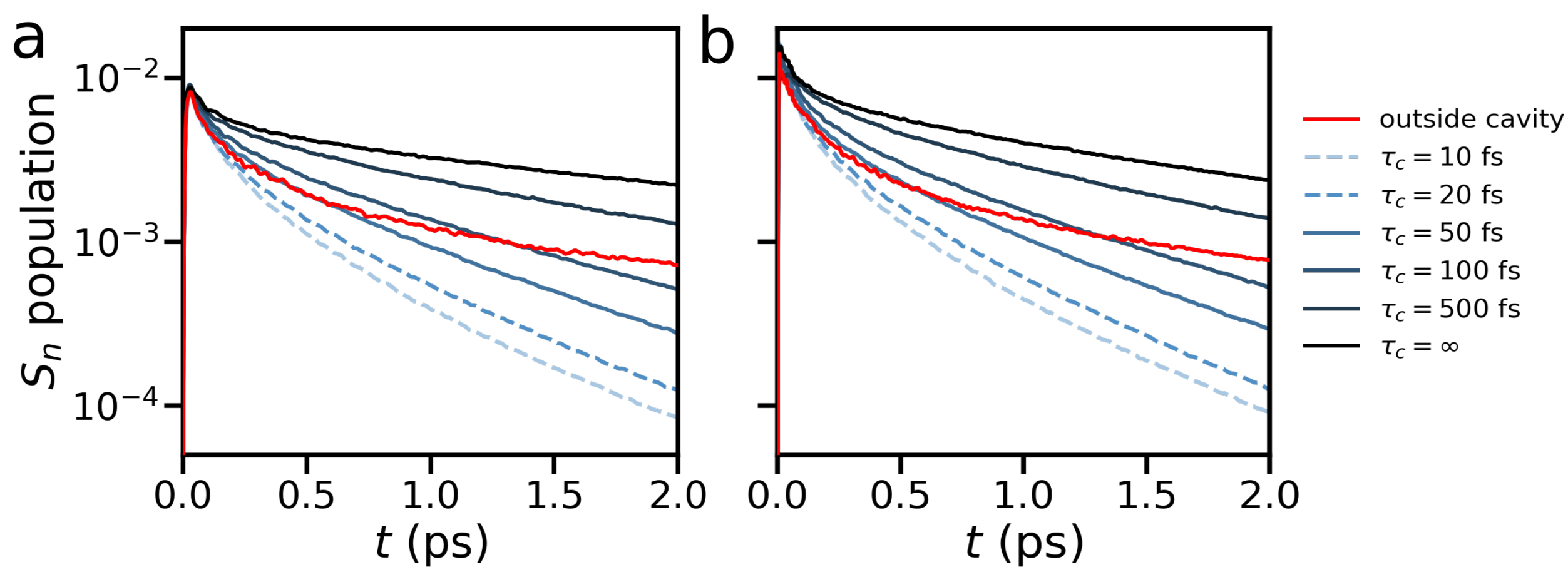

Figure S3: Total population of the $|\phi_{S_n,0}\rangle$ states in simulations of polariton dynamics in a system of $N = 50$ three-level molecules with an exciton coupling strength of $\langle J \rangle = 30$ meV and two different values of the EEA coupling strength and its standard deviation, namely $\langle V \rangle = 50$ meV and $\sigma_V = 50$ meV (panel **a**), and $\langle V \rangle = 20$ meV and $\sigma_V = 10$ meV (panel **b**). The simulations were performed with different cavity lifetimes ranging between $\tau_c = 10$ fs and $\tau_c = 500$ fs, as well as for the ideal cavity with no decay ($\gamma_c = 1/\tau_c = 0$) and outside the cavity. For both average values of the EEA coupling strength and its standard deviation, the dynamics of the total population of the $|\phi_{S_n,0}\rangle$ states are very similar and suggest a gradual transition from enahcement to suppression of the EEA rate with decreasing τ_c.

4 Additional results

4.1 Convergence of dynamics with respect to time step

When time-propagating an electronic wave function, the time step is usually chosen in the range from 0.01 fs to 0.1 fs. In particular, it is essential that the time step is smaller than the shortest time scale of the electronic dynamics. In the TLM-cavity system considered in this work, such a time scale is the period of Rabi oscillations, which is equal to $T_{\mathrm{R}} = 11$ fs for a Rabi splitting of $\hbar\Omega_{\mathrm{R}} = 375$ meV. We therefore selected a time step of $\Delta t = 1$ fs in numerical simulations, which is small enough to capture all the relevant effects during the dynamics and large enough to save computational resources. We also ensured that the resulting dynamics were consistent with those at a smaller time step of $\Delta t = 0.1$ fs, as shown in Figure S4 for the case of $N = 50$ TLMs without disorder, interacting with the coupling strength $\langle J \rangle = 50$ meV with each other.

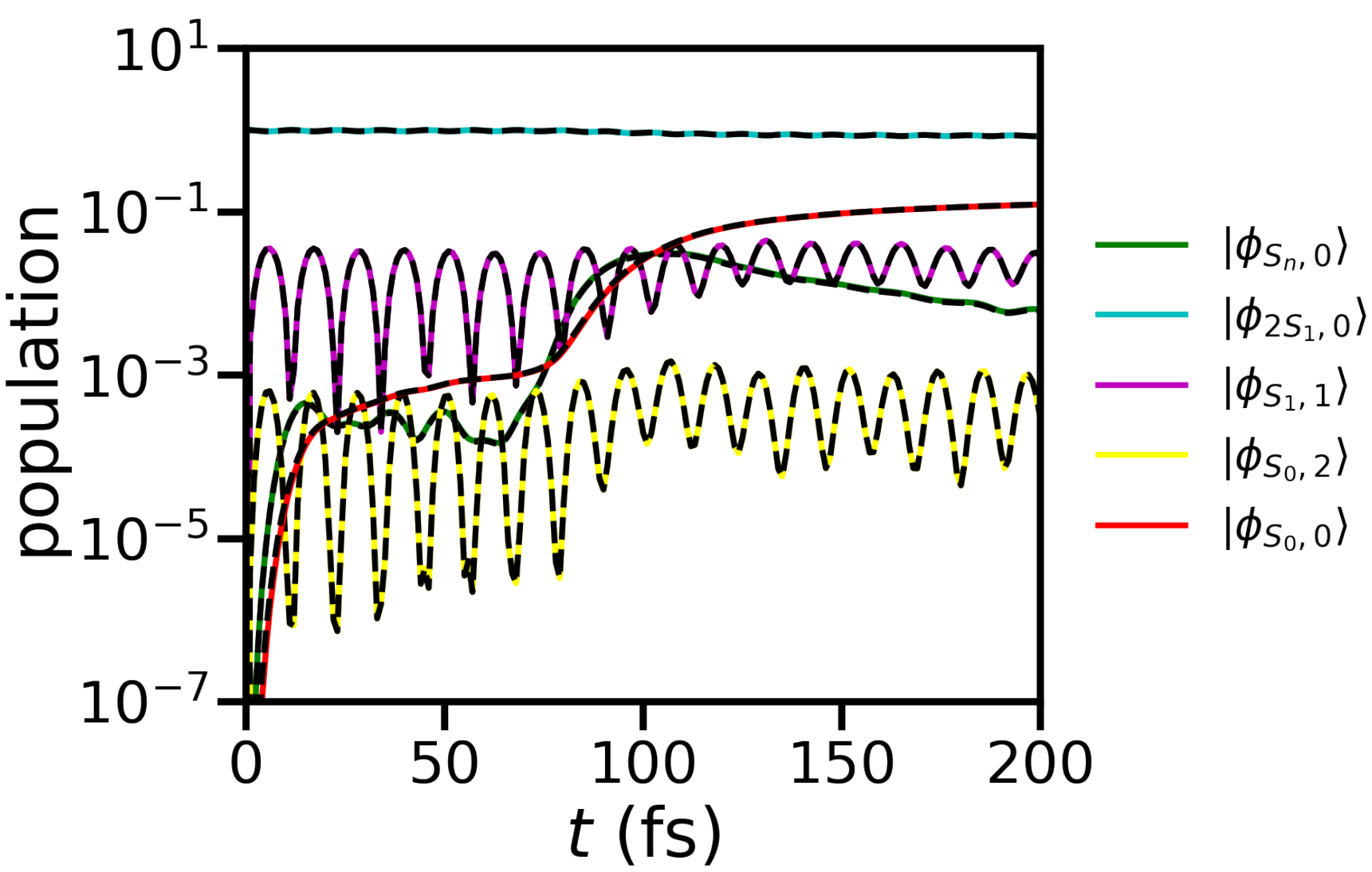

Figure S4: Total population of all $|\phi_{S_n,0}\rangle$ states (green), $|\phi_{2S_1,0}\rangle$ states (cyan), $|\phi_{S_1,1}\rangle$ states (purple), $|\phi_{S_0,2}\rangle$ state (yellow), and the ground state $|\phi_{S_0,0}\rangle$ (red), in simulation of $N = 50$ isoenergetic three-level molecules with an exciton coupling strength of $\langle J \rangle = 50$ meV, a collective light-matter coupling strength of $g\sqrt{N} = 175$ meV, and a time step of $\Delta t = 1$ fs. The black dashed lines show the same populations but in simulation with a time step of $\Delta t = 0.1$ fs.

4.2 Effect of cavity detuning on population dynamics

To explore the effect of cavity detuning on the dynamics of exciton-polaritons in the presence of exciton annihilation, we performed simulations of $N = 50$ TLMs interacting with strength $\langle J \rangle = \pm 50$ meV, in which the energy of the cavity mode was varied between the energy of the TLMs in the absence of disorder, $E_0 = 2.3$ eV, and the energy of the bright excitonic state, *i.e.* $E_{\text{br}} = E_0 - 2|\langle J \rangle| = 2.2$ eV for negative exciton coupling and $E_{\text{br}} = E_0 + 2|\langle J \rangle| = 2.4$ eV for positive exciton coupling. The resulting populations presented in Figure S5, exhibit a negligible dependence on cavity detuning. Importantly, the GS population for the chosen values of $\langle J \rangle$ is enhanced compared to that for TLMs uncoupled from the cavity (Figure 3 in the main text). This further confirms our claim that exciton delocalisation provided by the cavity is the main prerequisite for enhancement of EEA. Given that such delocalisation is achieved regardless of the magnitude of detuning as long as the system is able to reach strong coupling, it is understandable why the populations in Figure S5 are nearly independent on the cavity frequency, at least in the considered range of detunings.

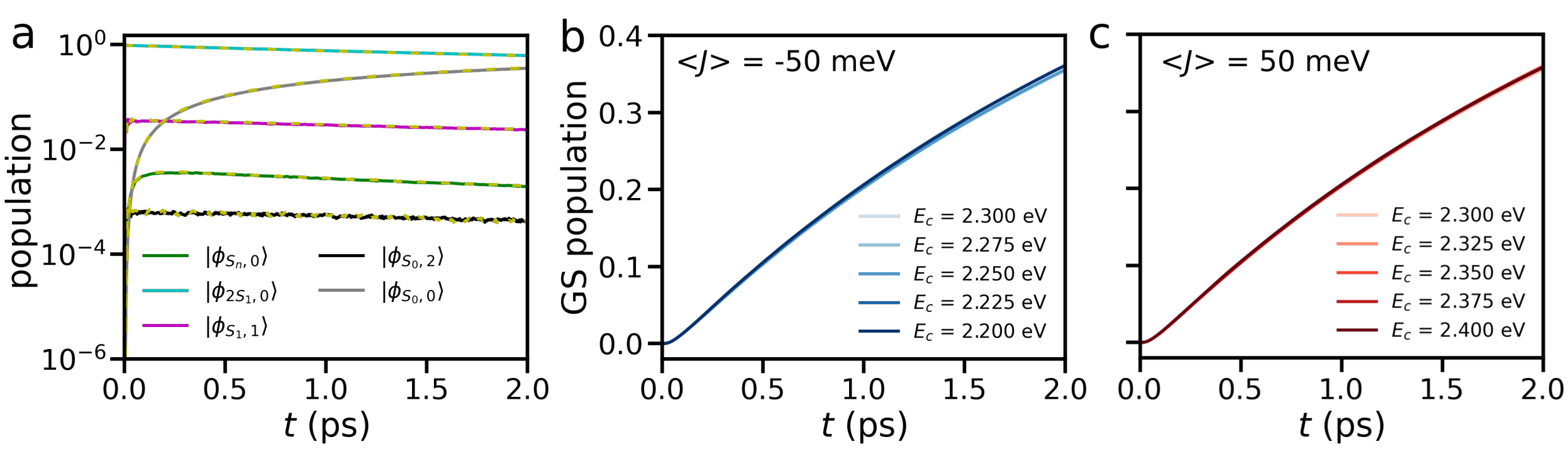

Figure S5: Panel **a**: Total population of all $|\phi_{S_n,0}\rangle$ states (green), $|\phi_{2S_1,0}\rangle$ states (cyan), $|\phi_{S_1,1}\rangle$ states (purple), $|\phi_{S_0,2}\rangle$ state (black), and the ground state $|\phi_{S_0,0}\rangle$ (grey), in simulation of $N = 50$ three-level molecules (TLMs) with an exciton coupling strength of $\langle J \rangle = 50$ meV, a collective light-matter coupling strength of $g\sqrt{N} = 175$ meV, a disorder strength of $\sigma_E = 100$ meV, and the cavity energy resonant with the TLMs' energy in the absence of disorder, i.e. $E_{\text{cav}} = E_0 = 2.3$ eV. These populations are compared with those corresponding to the cavity energy resonant with the bright excitonic states, *i.e.* $E_{\text{cav}} = E_0 + 2|\langle J \rangle| = 2.4$ eV (dashed yellow lines). Panels **b** and **c**: Ground state (GS) population in simulations with different energies of the cavity mode for a system with $\langle J \rangle = -50$ meV (panel b) and $\langle J \rangle = 50$ meV (panel c).

4.3 Short-time exciton dynamics

Figure S6**a** demonstrates the exciton probability density, P_{exc}, as a function of the TLMs' positions and time. The probability density at position j was calculated as $P_{\text{exc}}(t) = \frac{1}{2}\sum_{i=N+1}^{N(N-1)/2} |d_{i\in j}(t)|^2 + \frac{1}{2}\sum_{i=N(N-1)/2+1}^{N_{\text{st}}-1} |d_{i\in j}(t)|^2$, where the first term sums up the expansion coefficients of the total polariton wave function (Equation 3 in the main text) that correspond to $|\phi_{2S_1,0}\rangle$ states, while the second term sums up the expansion coefficients that correspond to $|\phi_{S_1,1}\rangle$ states. In the absence of light-matter coupling, only the first term remains. The subscript $i \in j$ implies that only those states are taken into account, which describe the excitation of molecule j into the S_1-state. Both terms are multiplied by $1/2$ because in both the $|\phi_{2S_1,0}\rangle$ states and the $|\phi_{S_1,1}\rangle$ states, only half the excitation falls on this molecule. The exciton probability density is normalised at each time step to give the total probability of finding an exciton in the chain of TLMs equal to one.

Figure S6**b** shows the population of the $|\phi_{S_n,0}\rangle$ states (green) and the GS population (grey). The build up of both populations starts after approximately 50 fs, which is the time required for the probability densities of two excitons to approach each other, allowing for population transfer from the states with two S_1-excitons to the states with one S_n-exciton.

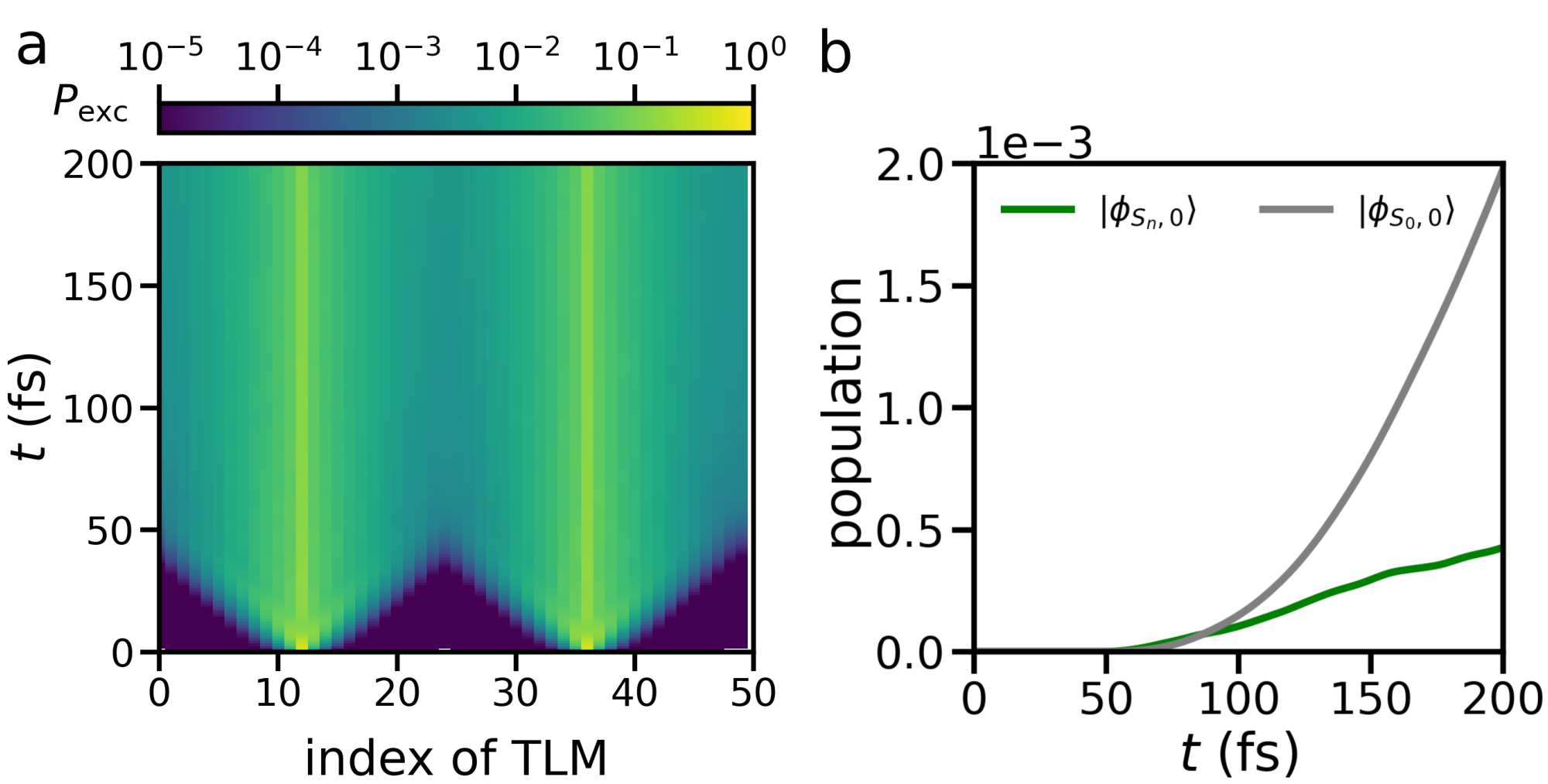

Figure S6: Panel **a**: Excitonic probability density, P_{exc}, as a function of time and the positions of three-level molecules, in simulations of exciton-polariton dynamics in a system of $N = 50$ molecules with an exciton coupling strength of $\langle J \rangle = 70$ meV outside the cavity, *i.e.* $g\sqrt{N} = 0$ meV. Panel **b**: Total populations of all $|\phi_{S_n,0}\rangle$ states (green) and of the ground state (grey).

4.4 Contribution of Rabi oscillations to the ground state population

To estimate the contribution of Rabi oscillations due to light-matter coupling to the total GS population, we performed simulation of polariton dynamics in the absence of exciton coupling, *i.e.* $J = 0$ meV. In this case, excitons are localised at the initial positions and hence unable to interact other than via Rabi oscillations. Figure S7**a** demonstrates that the GS population due to Rabi oscillations is very small compared to the GS population in the case when exciton interactions are included, *i.e.* $J > 0$. Moreover, the contribution of Rabi oscillations rapidly decreases with the number of molecules as $1/N$ (Figure S7**b**,**c**). Thus, enhancement of the EEA rate observed in a molecular system with low exciton mobility cannot be explained based on a simple Rabi oscillations argument. Instead, our simulations suggest that the cavity helps excitons to partially overcome disorder, leading to an increase in the likelihood of exciton interaction.

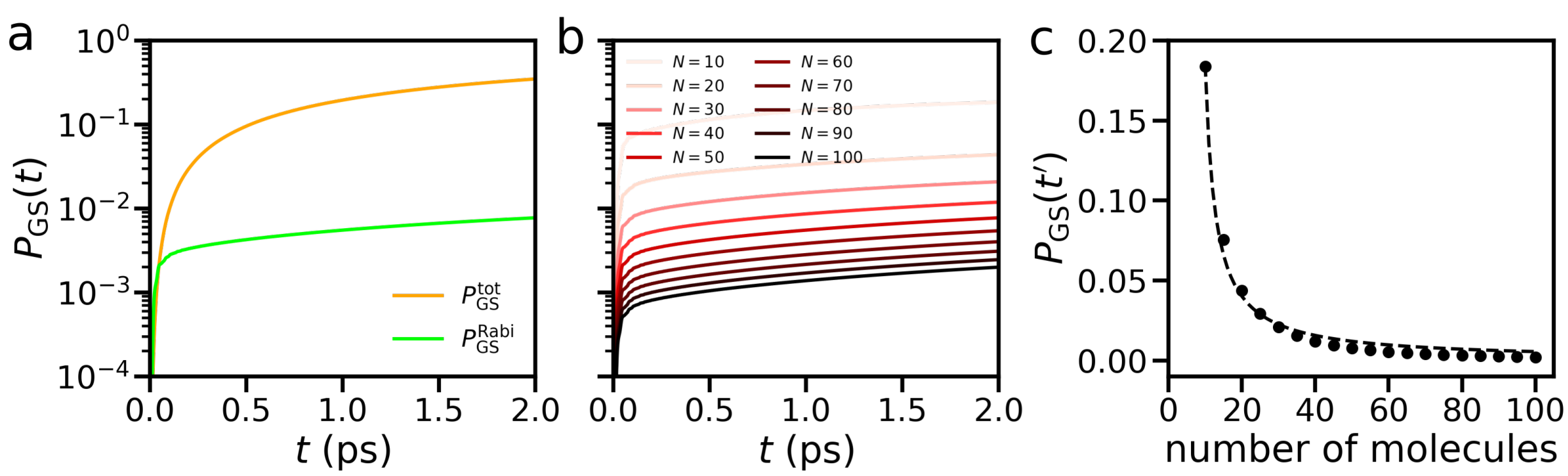

Figure S7: Panel **a**: Ground state (GS) population as a function of time in simulations of polariton dynamics in a system of $N = 50$ three-level molecules (TLMs) inside the cavity ($g\sqrt{N} = 175$ meV) with an exciton coupling strength of $\langle J \rangle = 70$ meV (orange) and $J = 0$ meV (green). Panel **b**: GS population in a system with $\langle J \rangle = 0$ meV with different numbers of TLMs. Panel **c**: GS population at time $t' = 2$ ps as a function of the number of molecules. The dashed line is a fit to function $\frac{a}{b+N}$ with $a = 0.513$ and $b = -7.229$.

4.5 Comparison between molecule-cavity systems with and without disorder

Figure S8**a**,**b** depicts the distribution of the exciton probability along the TLMs chain with $\langle J \rangle$ = 70 meV (panel **a**) and $\langle J \rangle = 30$ meV (panel **b**). Whereas outside the cavity (dashed lines), the exciton propagation is suppressed, and the probability density is mostly localised at the initial spot, especially in the case of low exciton coupling, strong light-matter coupling ensures exciton delocalisation inside the cavity, resulting in a non-zero uniform distribution of the probability density outside the initial spot (solid lines). Due to exciton delocalisation, EEA is almost as efficient at low exciton coupling as at high exciton coupling when molecules are placed inside the cavity in the presence of disorder ($\sigma_E = 100$ meV, solid lines in Figure S8**c**). Interestingly, in an ideal system without disorder ($\sigma_E = 0$ meV), the GS population, and hence the EEA rate, decreases with increasing exciton coupling strength. We attribute this effect to a decrease in the time that excitons spend near each other when $\langle J \rangle$ becomes higher, leading to less intense exciton annihilation.

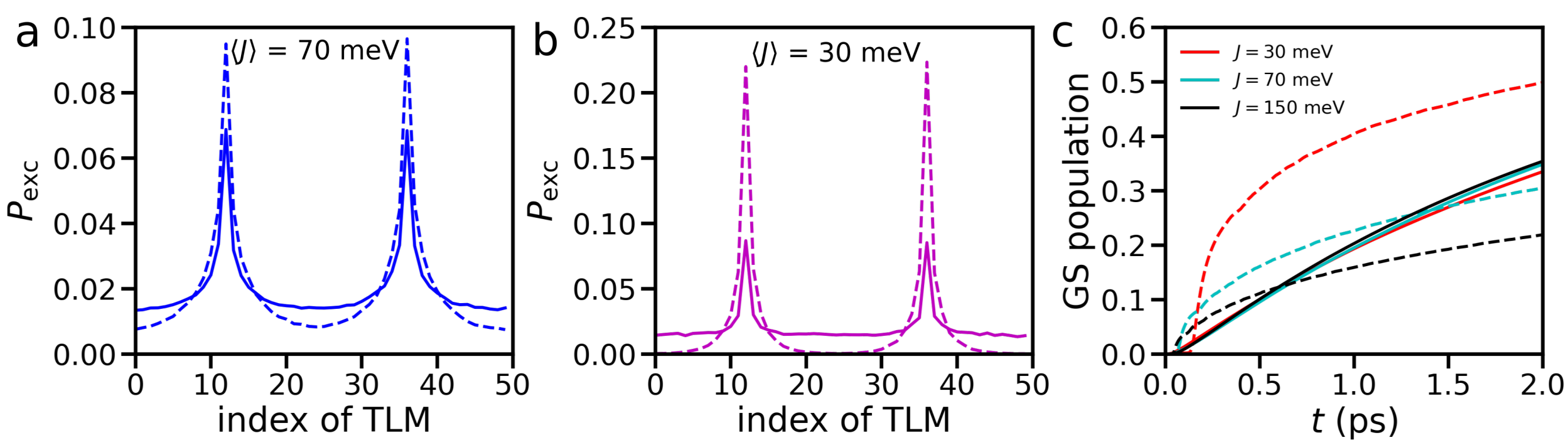

Figure S8: Panels **a**,**b**: Excitonic probability density, P_{exc}, as a function of the positions of three-level molecules (TLMs), in simulations of exciton-polariton dynamics in a system of $N = 50$ molecules with an exciton coupling strength of $\langle J \rangle = 70$ meV (panel **a**) and $\langle J \rangle = 30$ meV (panel **a**), outside the cavity ($g\sqrt{N} = 0$ meV, dashed lines) and inside the cavity ($g\sqrt{N} = 175$ meV, solid lines). The exciton population was averaged between $t = 1$ ps and $t = 2$ ps. Panel **c**: The ground state population as a function of time in simulations of exciton-polariton dynamics in a system of $N = 50$ TLMs with disorder ($\sigma_E = 100$ meV, solid lines) and without disorder ($\sigma_E = 0$ meV, dashed lines) under strong light-matter coupling. The exciton coupling strength was set to $\langle J \rangle = 30$ meV (red), $\langle J \rangle = 70$ meV (cyan), and $\langle J \rangle = 150$ meV (black).

4.6 Exciton propagation in systems with different exciton mobility

Figure S9 shows the excitonic probability amplitude, P_{exc}, as a function of the TLMs' positions and time in simulation of $N = 50$ TLMs under strong coupling with three different exciton coupling strengths, namely $\langle J \rangle = 20$ meV, 80 meV, and 140 meV. In the former case, exciton propagation is suppressed by disorder ($\sigma_E = 100$ meV), which is manifested as Anderson localisation of the exciton density. As $\langle J \rangle$ increases, exciton propagation becomes possible and the degree of the excitonic wave packet's propagation increases with the value of the exciton coupling strength. As a result, the EEA rate also increases (Figure 3**a** in the main text).

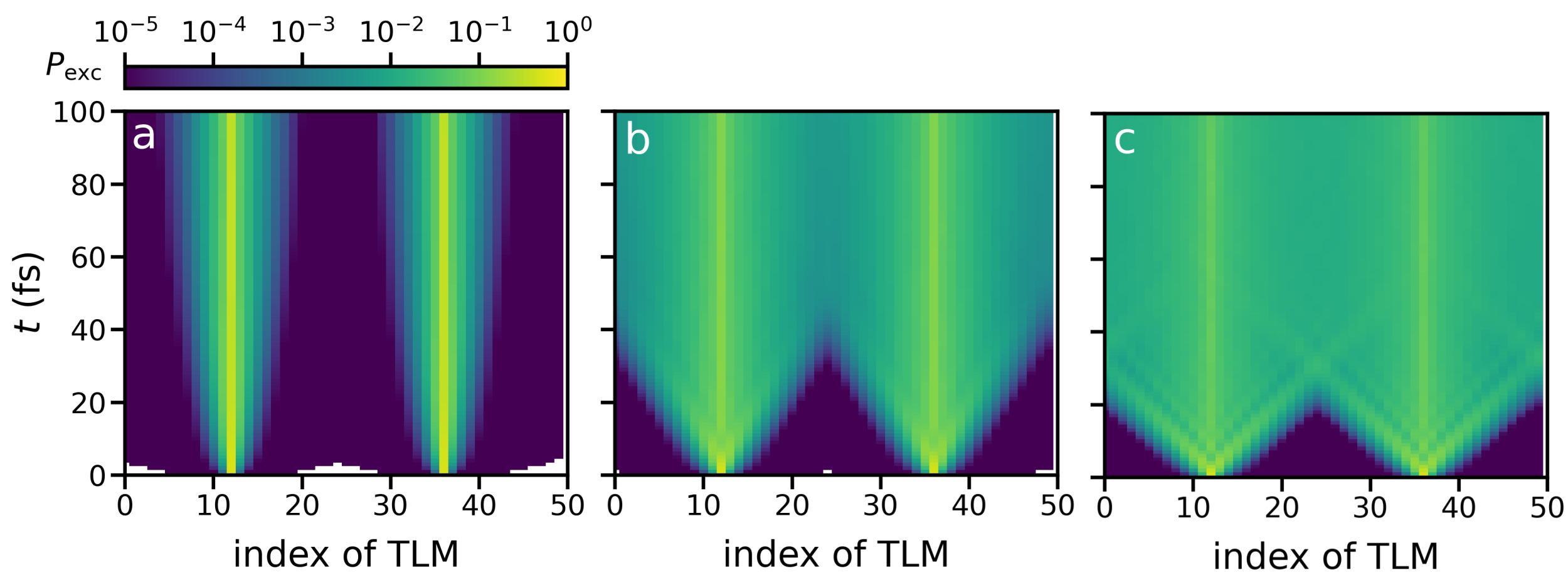

Figure S9: Excitonic probability density, P_{exc}, as a function of time and the positions of three-level molecules in simulations with $\langle J \rangle = 20$ meV (panel **a**), $\langle J \rangle = 80$ meV (panel **b**), and $\langle J \rangle = 140$ meV (panel **c**), under strong light-matter coupling. The excitation energy disorder strength is $\sigma_E = 100$ meV.

4.7 Exciton escape probability

To quantify how much of the exciton population extends beyond the initial excitation point, we computed the exciton escape probability using Equation 7 in the main text in a system of $N = 50$ TLMs. As shown in Figure S10, the average escape probability, $\overline{\chi}$, is rather small at low exciton coupling strength ($\overline{\chi} = 0.09$ at $\langle J \rangle = 20$ meV) outside the cavity due to Anderson localisation of excitons caused by disorder (Figure S9**a**). As $\langle J \rangle$ increases, exciton transport is less impeded by disorder, resulting in a gradual increase in $\overline{\chi}$, which reaches a value of $\overline{\chi} = 0.69$ at the highest exciton coupling strength of $\langle J \rangle = 150$ meV. In contrast, the escape probability is high ($\overline{\chi} > 0.60$) in the whole range of exciton coupling strengths between $\langle J \rangle = 20$ meV and $\langle J \rangle = 150$ meV inside the cavity and only slightly increases with $\langle J \rangle$. This is because a strong light-matter coupling extends the exciton probability density over the whole molecular chain (Figure S8**a**,**b**) regardless of the values of $\langle J \rangle$. This little dependence of the escape probability on exciton mobility correlates well with the little dependence of the EEA rate on $\langle J \rangle$ as shown in Figure 3**b** in the main text.

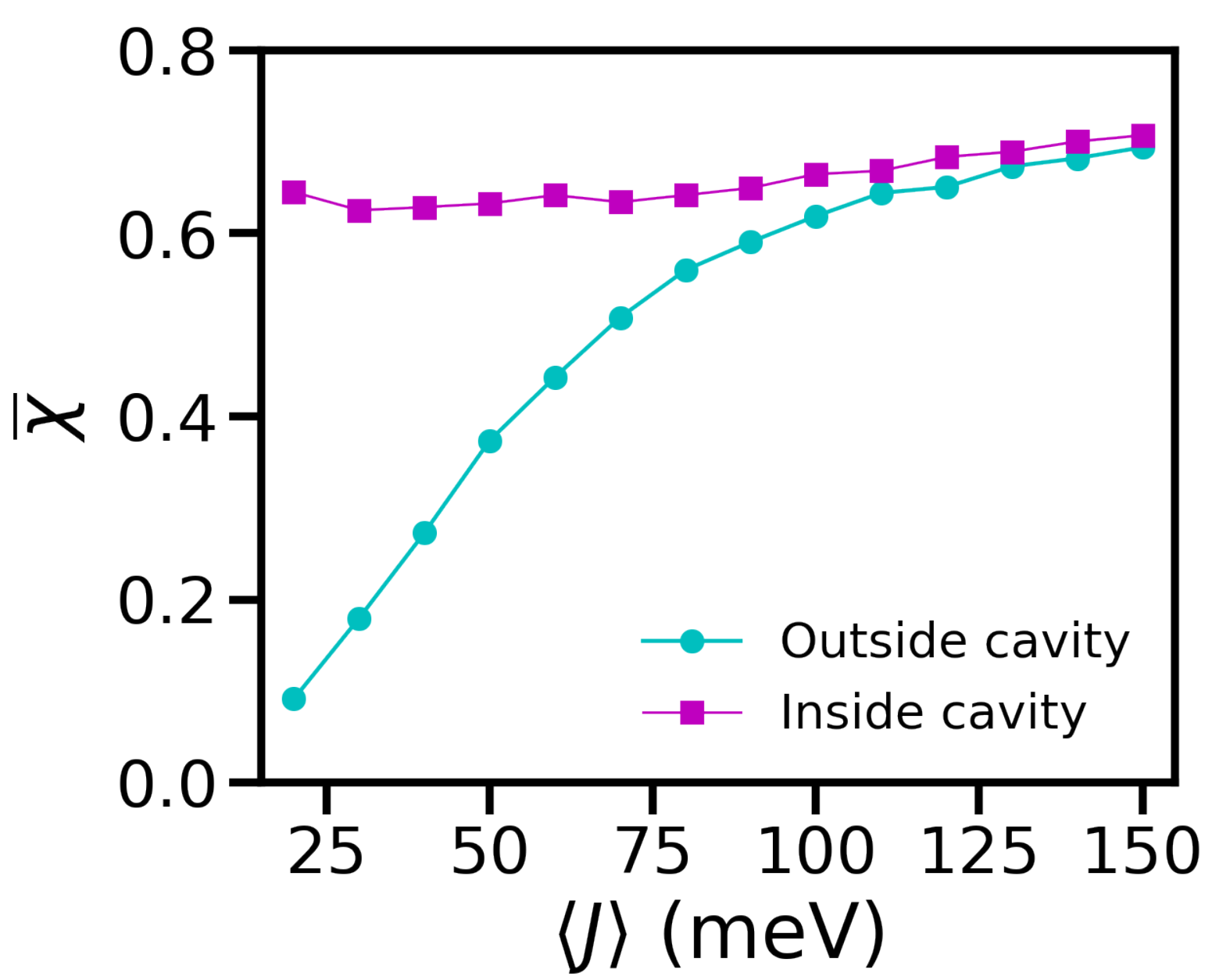

Figure S10: Exciton escape probability, $\overline{\chi}$, averaged between $t = 1$ ps and $t = 2$ ps, in simulation of $N = 50$ three-level molecules inside (purple squares) and outside the cavity (cyan circles) with different values of exciton coupling strength $\langle J \rangle$.

4.8 Estimation of the threshold density for the onset of EEA

In the main text, the threshold number of molecules, and hence the threshold exciton density, was estimated from the change in the slope of the α coefficient as a function of N, where α was extracted from fitting the GS populations in systems with different numbers of molecules to the power-law function, $P_{\text{GS}}(t) = \alpha t^{\beta}$. Similarly, we can estimate the threshold density from the change in the slope of the β coefficient, as shown in Figure S11. There, we can distinguish two regimes: a constant β at large N (low exciton density) and a linearly changing β at small N (large exciton density). The latter implies a faster initial growth of the GS population with increasing exciton density, as expected for the power-law function at^b with $0 < b < 1$, indicating an increase in the EEA rate after the exciton density exceeds a certain threshold. This threshold was estimated from the intersection of the lines corresponding to the two regimes of β (dashed lines in Figure S11). The resulting threshold density of $\rho = 5\%$ (or a threshold number of molecules of $N_{\text{th}} = 40$) for the system under strong light-matter coupling was smaller than the threshold density outside the cavity ($\rho = 10\%$, or $N_{\text{th}} = 20$), further supporting the conclusion that strong coupling can enhance exciton annihilation.

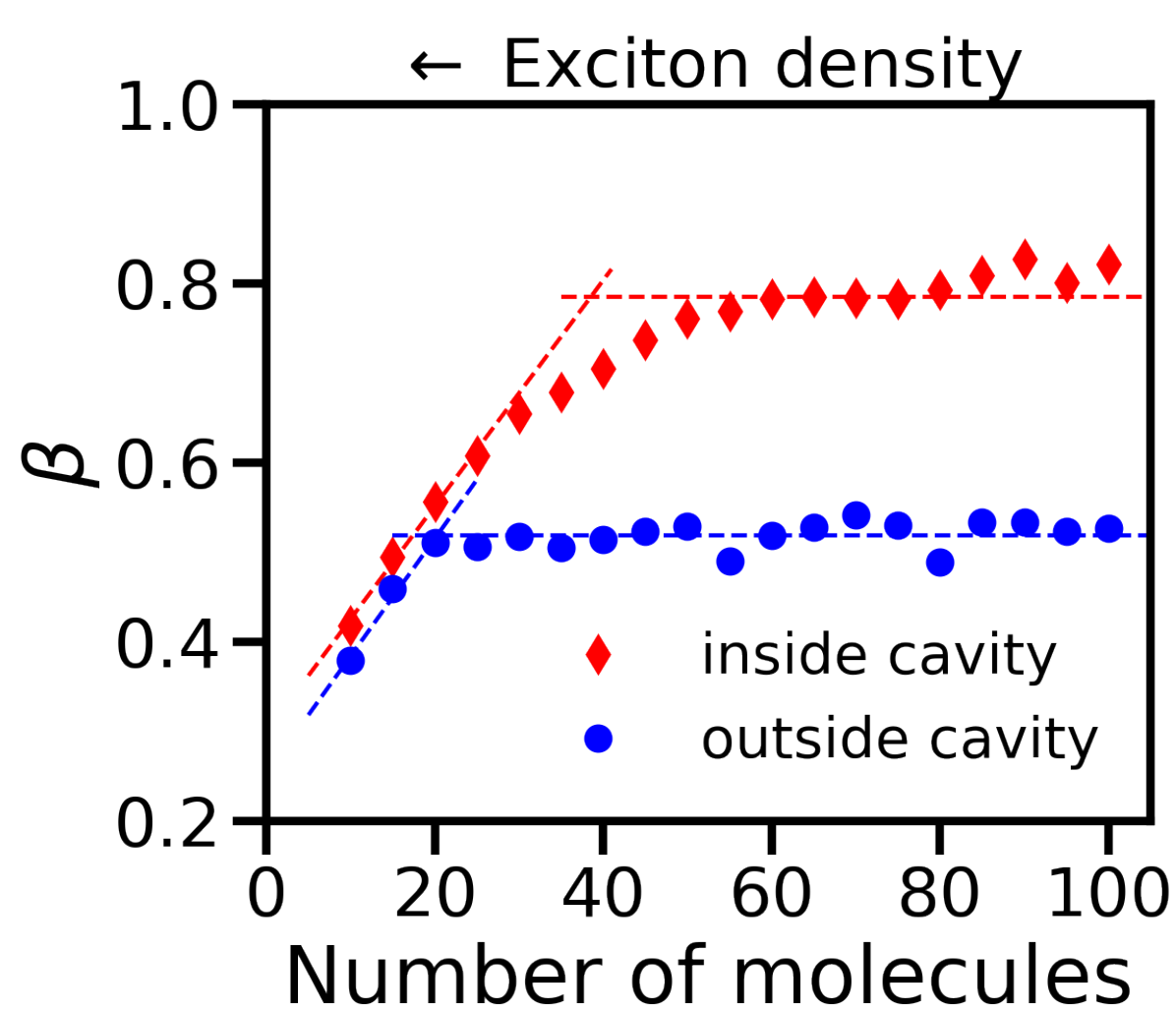

Figure S11: Dependence of β on the number of molecules, N, inside (red diamonds) vs. outside the cavity (blue circles), with β extracted from the fit of the GS population at different values of N in an ideal cavity ($\gamma_c = 0$) to the function $P_{\text{GS}}(t) = \alpha t^{\beta}$. The dashed lines are fits to a linear function. The intersection of the lines indicates the onset of EEA, which occurs at a larger N (smaller exciton density) when molecules are strongly coupled to the cavity.

4.9 Contribution of exciton-photon annihilation to the total EEA rate

Recently, it was proposed that in addition to exciton annihilation caused by the dipole-dipole interaction between a pair of molecules (*i.e.*, EEA), the cavity provides another source of exciton annihilation.[17] This so-called exciton-photon annihilation results from a resonant interaction between the $S_1 \to S_n$ molecular transition and a cavity photon, characterised by coupling strength $\tilde{g}$. To investigate the relevance of this effect in our system with the parameters of TLMs characteristic of R6G, we incorporated coupling terms $\tilde{g}$ in the system's Hamiltonian (Equation 2 in the main text) within the Rotating-wave approximation as follows:

$$\begin{aligned}\hat{H} \;=\; & \textstyle\sum_i^N E_{S_1,i}\hat{\sigma}^+_{S_1,i}\hat{\sigma}^-_{S_1,i} + E_{\mathrm{c}}\hat{a}^\dagger\hat{a} - \sum_i^N g\left(\hat{\sigma}^+_{S_1,i}\hat{a} + \hat{\sigma}^-_{S_1,i}\hat{a}^\dagger\right) - \\ & \textstyle\sum_i^N \tilde{g}\left(\hat{\sigma}^-_{S_1,i}\hat{\sigma}^+_{S_n,i}\hat{a} + \hat{\sigma}^+_{S_1,i}\hat{\sigma}^-_{S_n,i}\hat{a}^\dagger\right) + \\ & \textstyle\sum_{i,k}^N J_{i,k}\left(\hat{\sigma}^+_{S_1,i}\hat{\sigma}^-_{S_1,k} + \mathrm{h.c.}\right) + \\ & \textstyle\sum_j^N E_{S_n,j}\hat{\sigma}^+_{S_n,j}\hat{\sigma}^-_{S_n,j} + \sum_{i,k}^N V_{i,k}\left[\left(\hat{\sigma}^+_{S_n,i} + \hat{\sigma}^+_{S_n,k}\right)\hat{\sigma}^-_{S_1,i}\hat{\sigma}^-_{S_1,k} + \mathrm{h.c.}\right],\end{aligned} \tag{S7}$$

where the coupling terms responsible for exciton-photon annihilation are highlighted in pink.

With the obtained Hamiltonian, we repeated simulations of $N = 50$ TLMs with exciton coupling strength $\langle J\rangle = 50$ meV under strong light-matter coupling without cavity decay ($\gamma_c = 0$). Because the strongest oscillator strength for the $S_1 \to S_n$ transition ($f = 0.040$) with the energy difference close to resonance with the $S_0 \to S_1$ transition ($E_{S_n} - E_{S_1} = 3.341$ eV with $n = 19$) in the optimised geometry of the R6G monomer at the TD-DFT/ωB97X//def2-TZVPP level of theory is ≈ 28 times smaller than the oscillator strength for the $S_0 \to S_1$ transition ($f = 1.108$, $E_{S_1} - E_{S_0} = 3.394$ eV), we set $\tilde{g} = g/28$ in our simulations. With such a small coupling strength, exciton-photon annihilation has no contribution to the total EEA rate, as can be seen in the time-dependence of the GS population shown in Figure S12 (cyan squares and black dashed line).

In contrast to the R6G monomer, the ratio between the oscillator strengths corresponding to the $S_0 \to S_1$ and $S_1 \to S_n$ transitions is lower in the optimised geometry of the R6G H-dimer. Indeed, with oscillator strength $f = 1.128$ for the $S_0 \to S_1$ transition and oscillator strength $f = 0.200$ for the $S_1 \to S_{20}$ transition in one of the two monomers of the H-dimer (Table S1), the ratio is ≈ 6. Repeating exciton-polariton dynamics simulations with $\tilde{g} = g/6$ showed no difference in the GS population compared to the simulation with $\tilde{g} = 0$ (solid gray and black dashed lines in Figure S12), which indicated a negligible role of exciton-photon annihilation in the total EEA rate.

We note, however, that in molecular systems with comparable oscillator strengths for the $S_0 \to S_1$ and $S_1 \to S_n$ transitions, exciton-photon annihilation can become a significant source of exciton annihilation, as was confirmed by simulating TLMs under strong coupling with $\tilde{g} = g$ (brown solid line in Figure S12).

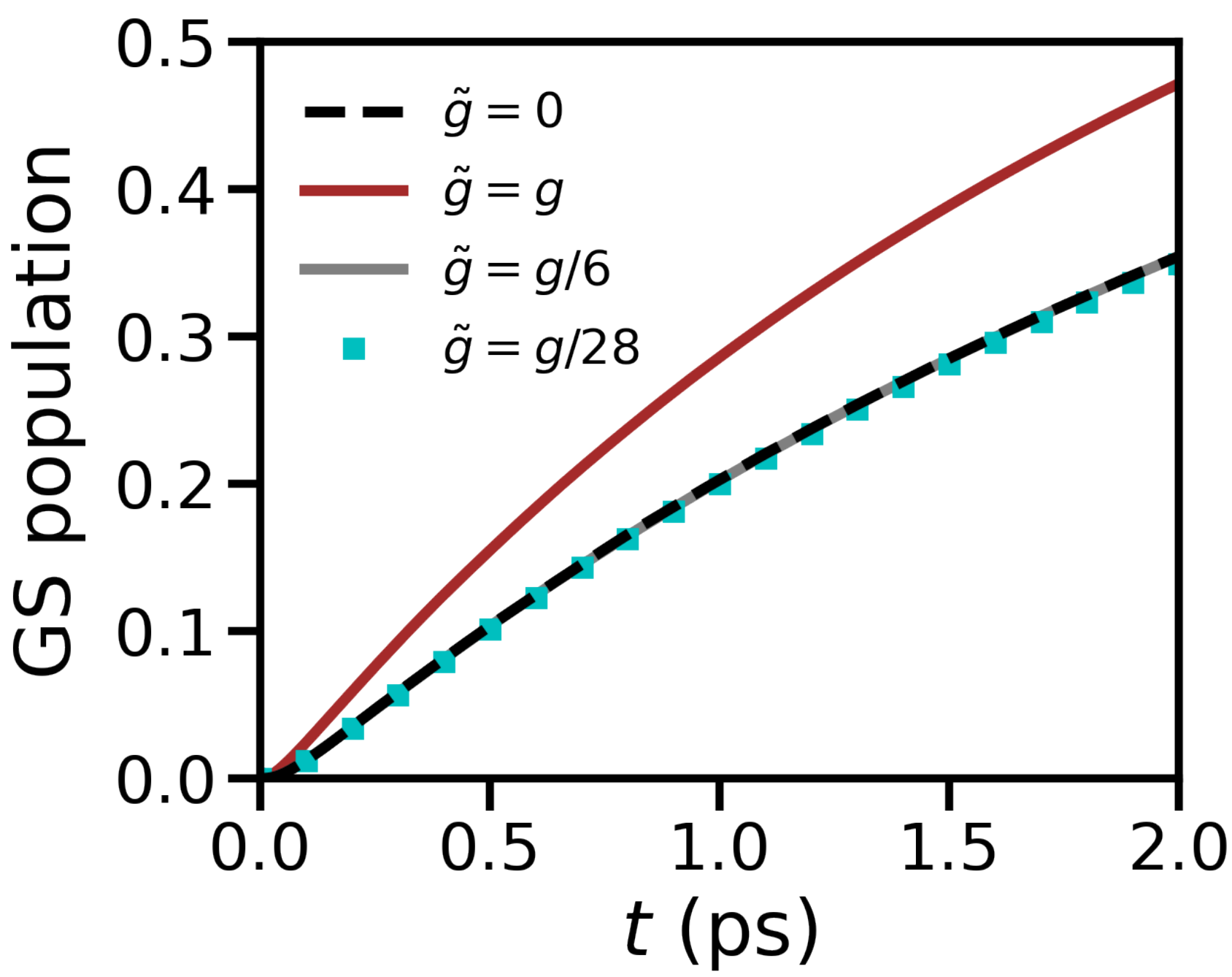

Figure S12: Ground state (GS) population as a function of time in simulations of polariton dynamics in a system of $N = 50$ three-level molecules (TLMs) inside the cavity ($g\sqrt{N} = 175$ meV) with an exciton coupling strength of $\langle J \rangle = 50$ meV. Additionally, coupling terms $\tilde{g}$ between the $|\phi_{S_n,0}\rangle$ and $|\phi_{S_1,1}\rangle$ states were included in the system's Hamiltonian (Equation S7) to account for exciton-photon annihilation. The following values of $\tilde{g}$ were chosen: $\tilde{g} = 0$ (dashed black line), $\tilde{g} = g$ (solid brown line), $\tilde{g} = g/6$ (solid gray line), and $\tilde{g} = g/28$ (cyan squares).

5 Effect of molecular dephasing on the EEA rate

The time evolution of a closed quantum system can be described using the time-dependent Schrödinger equation. In a system with electronic and photonic degrees of freedom, as is the case in the current study, the loss of population associated with the leakage of the electromagnetic field through the cavity mirrors can be treated implicitly by introducing an imaginary component to the corresponding diagonal elements of the system's Hamiltonian (Equation 2 in the main text). While such a method allows us to appreciate the effect of cavity decay on the system's dynamics, it does not treat this process explicitly, nor does it account for other processes resulting from interaction with the environment, such as molecular relaxation or decoherence. One of the methods to explicitly describe the evolution of an open quantum system in the limit of weak interaction with the environment is the Lindblad master equation. In standard form, this equation is defined as follows:[18]

$$\dot{\hat{\rho}} = -\frac{i}{\hbar}\left[\hat{H}, \hat{\rho}\right] + \sum_n \left(\hat{L}_n \hat{\rho} \hat{L}_n^\dagger - \frac{1}{2}\{\hat{L}_n^\dagger \hat{L}_n, \hat{\rho}\}\right) \tag{S8}$$

with $\hat{\rho}$ being the density matrix, $\hat{L}_n$ being a Lindblad jump operator associated with the nth interaction channel with the environment, and the sum spanning all interaction channels.

Without population relaxation ($\gamma_c = \gamma_{\text{mol}} = 0$) and in the presence of pure dephasing, $\hat{L}_{\text{deph}} = \sqrt{\gamma_{\text{deph}}}\hat{\sigma}_z$, the Lindblad master equation takes the following form:

$$\dot{\hat{\rho}} = -\frac{i}{\hbar}\left[\hat{H}, \hat{\rho}\right] + \gamma_{\text{deph}} \hat{\sigma}_z \hat{\rho} \hat{\sigma}_z^\dagger - \frac{\gamma_{\text{deph}}}{2}\{\hat{\sigma}_z^\dagger \hat{\sigma}_z, \hat{\rho}\}. \tag{S9}$$

Because $\hat{\sigma}_z^\dagger \hat{\sigma}_z = \hat{\mathbb{I}}$ and $\hat{\sigma}_z = \hat{\sigma}_z^\dagger$, the master equation is reduced to

$$\dot{\hat{\rho}} = -\frac{i}{\hbar}\left[\hat{H}, \hat{\rho}\right] + \gamma_{\text{deph}} \left(\hat{\sigma}_z \hat{\rho} \hat{\sigma}_z - \hat{\rho}\right). \tag{S10}$$

With inclusion of the Lindblad operator responsible for cavity decay, $\hat{L}_c = \sqrt{\gamma_c}\hat{a}$, the master equation is transformed to

$$\dot{\hat{\rho}} = -\frac{i}{\hbar}\left[\hat{H}, \hat{\rho}\right] + \gamma_{\text{deph}}\left(\hat{\sigma}_z\hat{\rho}\hat{\sigma}_z - \hat{\rho}\right) + \gamma_c\hat{a}\hat{\rho}\hat{a}^\dagger - \frac{\gamma_c}{2}\{\hat{a}^\dagger\hat{a}, \hat{\rho}\}. \tag{S11}$$

5.1 Jaynes-Cummings model

Consider a system of one molecule strongly interacting with one cavity photon. Under the rotating-wave approximation, this system can be described by the Jaynes-Cummings Hamiltonian:[19]

$$\hat{H}_{\text{JC}} = \hbar\omega_m\hat{\sigma}^+\hat{\sigma}^- + \hbar\omega_c\hat{a}^\dagger\hat{a} + \hbar g\left(\hat{\sigma}^+\hat{a} + \hat{\sigma}^-\hat{a}^\dagger\right). \tag{S12}$$

An analytical solution can be obtained for the master equation with the Jaynes-Cummings Hamiltonian in the presence of dephasing and cavity decay. In the case $\gamma_{\text{deph}}, \gamma_c \ll \Omega_{\text{R}}$ and initial excitation into the molecule, the solution looks as follows:[20,21]

$$P_e(t) \approx \frac{1}{2}\left[1 + e^{-\gamma_{\text{deph}}t/2}\cos\left(\Omega_{\text{R}}t\right)\right]e^{-\gamma_c t/2}. \tag{S13}$$

Figure S13 shows the time-evolution of the molecular and photonic populations in the Jaynes-Cummings model with molecular dephasing (panel **a**) and with both dephasing and cavity decay (panel **b**). The population of the molecular component obtained by solving the Lindblad master equation (Equation S11; dashed black lines) is identical to the analytical solution (blue diamonds), demonstrating the ability of the Lindblad master equation to correctly capture the effect of cavity decay and molecular dephasing.

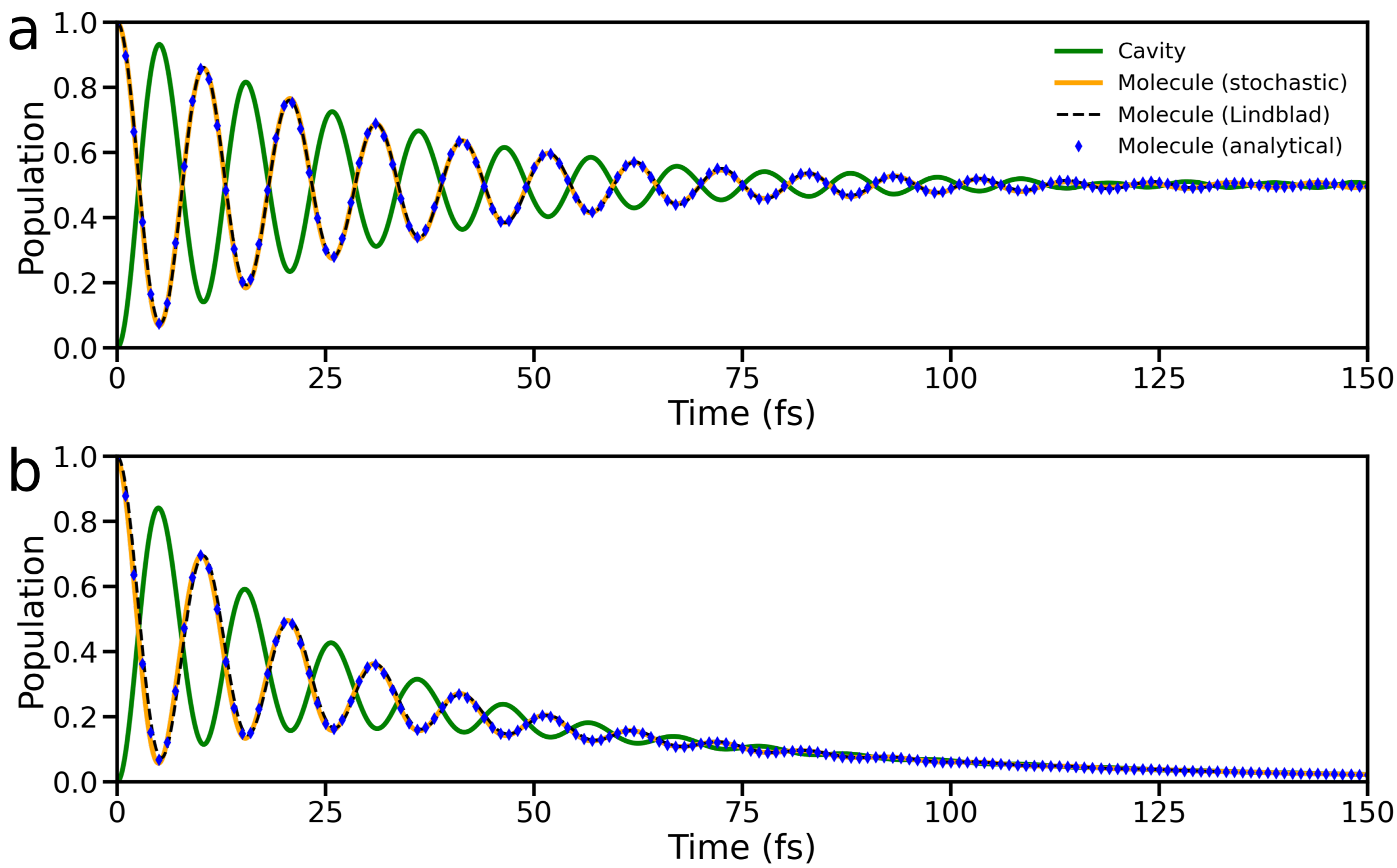

Figure S13: Population of the cavity mode (solid green lines) and of the molecular excitation in the Jaynes-Cummings model under the rotating-wave approximation (Equation S12) in the presence of (**a**) dephasing ($\tau_{\text{deph}} = 100$ fs) and (**b**) dephasing and cavity decay ($\tau_c = 150$ fs). Population of the molecular excitation is shown for the case of the direct solution of the Lindblad master equation (Equation S8, dashed black line) and the Monte Carlo wave function (MCWF) propagation (solid orange lines) and compared with the analytical solution (Equation S13 with $\gamma_c = 0$ (**a**) and $\gamma_c = 1/\tau_c$ (**b**), blue diamonds). The populations obtained with the MCWF propagation were averaged over 5000 runs.

5.2 Simulation of EEA with the Lindblad master equation

5.2.1 Computational scaling of direct Lindblad simulations

To assess the feasibility of simulating EEA using direct integration of the Lindblad master equation, we analyse the computational scaling of a bespoke solver (which is part of the Jones Group SpecSuite[22,23]) with system size. In this framework, the dominant numerical cost arises from dense linear algebra operations acting on the system density matrix. The computational cost is therefore governed primarily by the Hilbert space dimension d.

Benchmark simulations were performed for systems of $N = 10$ and $N = 20$ TLMs under identical numerical conditions (identical propagation window and solver parameters). The corresponding Hilbert space dimensions are $d_{10} = 67$ and $d_{20} = 232$, with measured runtimes for a single propagation chunk ($0 \rightarrow 0.001$ ps, corresponding to 20 HEOMsolve steps) of 18 seconds and 21.6 minutes, respectively. These values imply a power-law scaling of the form

$$t_{\text{chunk}} \sim d^p, \tag{S14}$$

with an exponent $p \approx 4.9$. This indicates a very rapid increase in computational cost with system size.

We extrapolate up to a single 0.2 ps propagation chunk, and a full $2\,\mathrm{ps}$ run for the following analysis. Using the measured runtime for $N = 20$ as a reference, we would require approximately 121 days for $N = 30$, 5 years for $N = 40$, and over 40 years for $N = 50$. Extending to the full simulation time of 2 ps (corresponding to 10 of the $0.2\,\mathrm{ps}$ chunks) results in total runtimes ranging from several days ($N = 10$) to hundreds of years ($N = 50$).

5.2.2 Implications for EEA simulations

The steep scaling of direct Lindblad simulations imposes severe limitations on the accessible system sizes. While systems up to $N \sim 20$ can be treated using direct density matrix propagation, larger ensembles rapidly become computationally prohibitive. Importantly, this limitation arises from the growth of the Hilbert space rather than memory usage, which remains relatively modest for the system sizes considered.

5.2.3 Projected Runtimes for Larger Systems

Using the measured $N = 20$ chunk runtime as a reference,

$$t^{(\text{chunk})}(N) = t_{20}^{(\text{chunk})} \left(\frac{d(N)}{d_{20}} \right)^{4.9}, \tag{S15}$$

with $t_{20}^{(\text{chunk})} \approx 1.3$ days, and taking the Hilbert space dimensions as

$$d_{20} = 232,\; d_{30} = 496,$$
$$d_{40} = 861,\; d_{50} = 1326,$$

we obtain the per-chunk runtimes for a single 0.200 ps propagation as displayed in Table S3.

Table S3: Estimated per-chunk runtimes.

N	$d(N)$	$t^{(0.001\,\text{chunk})}$ (mixed)	$t^{(0.2\,\text{chunk})}$ (mixed)
20	232	21.6 minutes	3 days
30	496	14.5 hours	121 days
40	861	8.75 days	4.8 years
50	1326	72.9 days	40 years

5.2.4 Full 2.0 ps Runtime Estimates

Since the target simulation length is 2.0 ps and we have measured chunks of 0.2 ps, the full simulation requires approximately

$$N_{\text{chunks}} = \frac{2.0}{0.2} = 10, \tag{S16}$$

and hence

$$t^{(\text{full})}(N) \approx 10\, t^{(\text{chunk})}(N). \tag{S17}$$

Table S4 gives the approximate full runtimes.

Table S4: Estimated full runtimes for a 2.0 ps simulation (10 0.2 ps chunks).

N	$t^{(\text{full})}$ (hours)	$t^{(\text{full})}$ (days)	$t^{(\text{full})}$ (mixed units)
10	10	0.42	10 hours
20	720	30	1 month
30	2.9×10^4	1210	3.3 years
40	4.2×10^5	1.75×10^4	48 years
50	3.5×10^6	1.46×10^5	400 years

These considerations strongly motivate the use of stochastic wave function approaches, such as the Monte-Carlo wave function (MCWF) method described below. By propagating pure states instead of density matrices, the MCWF method avoids the quadratic scaling associated with the density matrix dimension and enables simulations of significantly larger molecular ensembles. This allows us to access the regime of $N = 50$ molecules considered in the main text, while still incorporating the effects of dephasing and cavity decay.

In practice, the MCWF approach introduces an additional statistical averaging step over stochastic trajectories. However, as demonstrated in Figure S13, convergence to the Lindblad master equation results is achieved with a manageable number of trajectories, making this approach a computationally efficient alternative for large-scale EEA simulations.

5.2.5 Memory requirement of direct Lindblad solution

In addition to computational cost, practical limitations arise from memory usage and data transfer. In the standard representation, the density matrix is stored as a full complex matrix of size $d \times d$, where d is the Hilbert space dimension. A single density matrix therefore requires storage that scales as $\mathcal{O}(d^2)$.

For illustration, a system with $d = 20$ corresponds to a 20×20 complex matrix, i.e. 400 complex numbers, which is negligible in terms of memory. However, for system sizes relevant to this work ($d \sim 10^2$), the storage becomes significantly larger. For example, $d = 200$ corresponds to 4×10^4 complex numbers per density matrix. When storing a full trajectory over $N_{\text{save}} \sim 10^3$ time points, this results in $\sim 4 \times 10^7$ complex numbers, corresponding to several hundred megabytes per simulation when using double-precision complex arithmetic.

In the present implementation, the dynamics are propagated in the Fortran solver using a fixed internal time step of 50 as up to the end of each propagation chunk. However, the density matrix is not written to disk at every internal step, but only at a smaller set of output times chosen for analysis and plotting. As a result, the computational cost and storage cost are controlled by different time scales.

If N_{step} denotes the number of internal propagation steps and N_{save} the number of saved time points, then the total storage required for a full trajectory scales as

$$\mathcal{O}(N_{\mathrm{save}} d^2), \tag{S18}$$

whereas the dominant computational effort scales as

$$\mathcal{O}(N_{\mathrm{step}} d^3), \tag{S19}$$

since each internal propagation step requires dense matrix operations such as commutators and dissipative terms. Because $N_{\mathrm{step}} \gg N_{\mathrm{save}}$, the runtime is determined by the fine internal propagation grid, while the data volume is determined by the coarser saved output grid.

Even when the number of saved time points is moderate, the dense representation leads to large output files for increasing system size. In practice, this makes it difficult to transfer full simulation results between local machines and ARCHER2 (a supercomputer used in the current work), particularly when multiple runs or parameter sweeps were required. This limitation arises primarily from the cumulative size of dense trajectory data and associated input/output overhead.

To address these issues, we implemented a bespoke sparse matrix formulation of the Lindblad dynamics. In this approach, the Hamiltonian and Lindblad operators are stored in sparse format, retaining only the non-zero matrix elements and their indices. This is justified by the structure of the exciton–photon model, in which couplings are restricted to specific manifolds (e.g. nearest-neighbour exciton hopping, light–matter coupling, and ladder-type photon transitions), resulting in a large fraction of zero entries in the full matrices.

The sparse implementation therefore reduces memory usage from $\mathcal{O}(d^2)$ to $\mathcal{O}(\mathrm{nnz})$, where nnz is the number of non-zero elements, which scales much more favourably with system size. In addition, storing trajectories in sparse format significantly reduces file sizes, making it feasible to transfer and analyse simulation outputs.

Overall, while dense representations are straightforward to implement, their quadratic scaling in both memory and storage makes them impractical for large-scale simulations. The adoption of a sparse representation provides a practical route to overcoming these limitations in exciton–polariton dynamics. However, overall, the good agreement between the MCWF formulation and the full Lindblad (at least to this level of approximation of physical processes) makes it highly preferable for the chosen system.

5.3 Simulation of EEA with the Monte-Carlo wave function propagation

Due to the unfortunate scaling of the simulation time with the system size, we were unable to propagate the density matrix for molecule-cavity systems with $N > 20$ with the Lindblad master equation. Therefore, to study EEA in such large molecular ensembles, we instead propagated the full exciton-polariton wave function (Equation 3 in the main text) together with stochastic Markovian jumps associated with the Lindblad jump operators, responsible for dephasing and cavity decay. In the MCWF method,[24,25] the expansion coefficients of the wave function are numerically propagated according to the Schrödinger equation (Equation 4 in the main text). At each time step of a simulation, the norm of the wave function is reduced according to

$$|\Psi(t + \Delta t)|^2 = 1 - \delta p, \tag{S20}$$

where Δt is the time step size, and $\delta p = \sum_n \delta p_n$ with δp_n being the contribution of the nth jump operator to the total reduction of the wave function's norm, defined as $\delta p_n = \langle\Psi(t)|\hat{L}_n^\dagger \hat{L}_n|\Psi(t)\rangle$.[26,27] Then, a number ξ is randomly drawn from the uniform distribution between zero and one, $0 < \xi < 1$, and is compared to δp.

In most cases, δp is smaller than ξ, and we assume that no quantum jump has occurred. In this case, the wave function is simply renormalised before the next time step:

$$|\Psi_{\text{renorm}}(t + \Delta t)\rangle = \frac{|\Psi(t + \Delta t)\rangle}{\sqrt{1 - \delta p}}. \tag{S21}$$

Occasionally, however, the condition $\delta p > \xi$ is fulfilled. In this situation, we assume that a quantum jump has occured, and a Lindblad operator then acts on the wave function. The choice of a specific operator to act on the wave function is made randomly with weights given by $\delta p_n/\delta p$.

When the selected operator is associated with pure dephasing, $\hat{L}_n = \sqrt{\gamma_{\text{deph}}}\hat{\sigma}_z$, the amplitude of the expansion coefficient of the state, on which the dephasing operator acts, receives a random phase shift,

$$|d_n(t+\Delta t)| \rightarrow |d_n(t+\Delta t)| \exp\left(i\zeta\right), \tag{S22}$$

with ζ being a random phase number in the range $[0; 2\pi]$.

When the selected operator is associated with cavity decay, $\hat{L}_n = \sqrt{\gamma_c}\hat{a}$, the system collapses into the total ground state. Because this state is not explicitly included in the system's Hamiltonian, we set all expansion coefficients to zero until the end of the simulation, *i.e.* $d_j = 0\ \forall j$, resulting in the norm of the wave function being zero at any time after the jump.

In this work, we consider four types of operators acting on different exciton-photon product states, namely

1. pure dephasing operator $\hat{L}_{\text{deph}} = \sqrt{\gamma_{\text{deph}}}\hat{\sigma}_z$, acting on the states, in which only one TLM is excited, *i.e.* $|\phi_{S_n,0}\rangle$ and $|\phi_{S_1,1}\rangle$;

2. pure dephasing operator $\hat{L}'_{\text{deph}} = \sqrt{2\gamma_{\text{deph}}}\hat{\sigma}_z$, acting on the states, in which two TLMs are simultaneously excited, *i.e.* $|\phi_{2S_1,0}\rangle$;

3. cavity decay operator $\hat{L}_c = \sqrt{\gamma_c}\hat{a}$, acting on the states, in which only one cavity photon is present, *i.e.* $|\phi_{S_1,1}\rangle$;

4. cavity decay operator $\hat{L}'_c = \sqrt{2\gamma_c}\hat{a}$, acting on the state, in which two cavity photons are present, *i.e.* $|\phi_{S_0,2}\rangle$.

We first validated the MCFW approach against directly solving the Lindblad master equation for the Jaynes-Cummings model. As shown in Figure S13, the populations of the molecular excited state obtained with the two approaches (solid orange line and dashed black line) agree very well

when *i)* only dephasing is present and *ii)* both dephasing and cavity decay are included, as long as the results obtained with the MCFW method are averaged over a sufficiently large number of runs (in Figure S13, the populations are shown for $N_{\text{runs}} = 5000$).

Next, we compared the two methods for the TLM-cavity system considered in the current work. Figure S14 shows the populations of the product states for a system of $N = 10$ and $N = 20$ TLMs in the absence and presence of light-matter coupling. Although the agreement between the methods is not perfect, the populations obtained with the MCFW are much closer to those obtained by directly solving the Lindblad master equation than to the populations obtained by numerical propagation of the Schrödinger equation in the absence of dephasing (pink lines in Figure S14). Therefore, we reckon it reasonable to use the MCFW for studying the effect of dephasing on EEA in large molecular ensembles with $N > 20$, which are not tractable with the full Lindblad equation.

After validating the MCFW approach, we performed exciton-polariton dynamics simulations with $N = 50$, which is larger than the number of molecules that can be handled using the Lindblad master equation with our available computational resources. As Figure 5 in the main text depicts, pure dephasing results in a more rapid depopulation of the initially excited states with two S_1-excitons, *i.e.* $|\phi_{2S_1,0}\rangle$, and hence a more rapid build-up of the population of the remaining states, including the ground state $|\phi_{S_0,0}\rangle$ (grey lines). In Figure S15, we also show the GS population in simulations with different cavity lifetimes, $\tau_c = 1/\gamma_c$. Although dephasing leads to a quantitative change in the GS population for all lifetimes, the trends remain similar to those in the system without dephasing. Importantly, EEA undergoes a transition from suppression at short lifetimes to enhancement at long lifetimes, in line with simulations without dephasing.

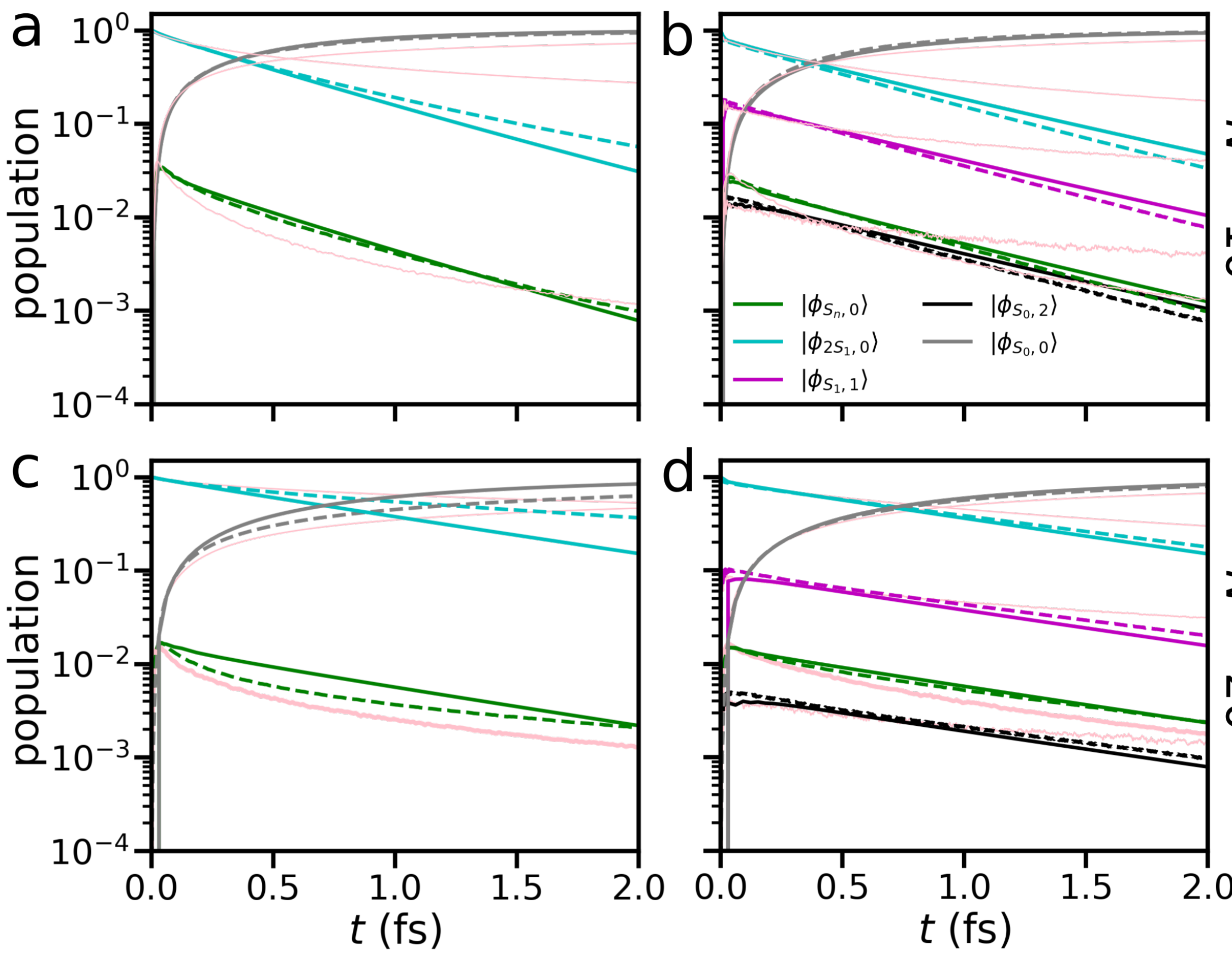

Figure S14: Total population of all $|\phi_{S_n,0}\rangle$ states (green), $|\phi_{2S_1,0}\rangle$ states (cyan), $|\phi_{S_1,1}\rangle$ states (purple), $|\phi_{S_0,2}\rangle$ state (black), and the ground state $|\phi_{S_0,0}\rangle$ (grey), in simulation of $N = 10$ three-level molecules (TLMs) with an exciton coupling strength of $\langle J \rangle = 50$ meV and a collective light-matter coupling strength of $g\sqrt{N} = 0$ meV (panel **a**) and $g\sqrt{N} = 175$ meV (panel **b**). In both panels, the solid lines correspond to direct solution of the Lindblad master equation, and the dashed lines correspond to the Monte Carlo wave function (MCWF) propagation with the inclusion of dephasing ($\tau_{\text{deph}} = 100$ fs). The pink lines show the populations of product states in simulations without dephasing. The populations obtained with the Lindblad master equation and with the MCWF propagation were averaged over 100 and 10000 runs, respectively. Panels **c** and **d**: same but for $N = 20$ molecules.

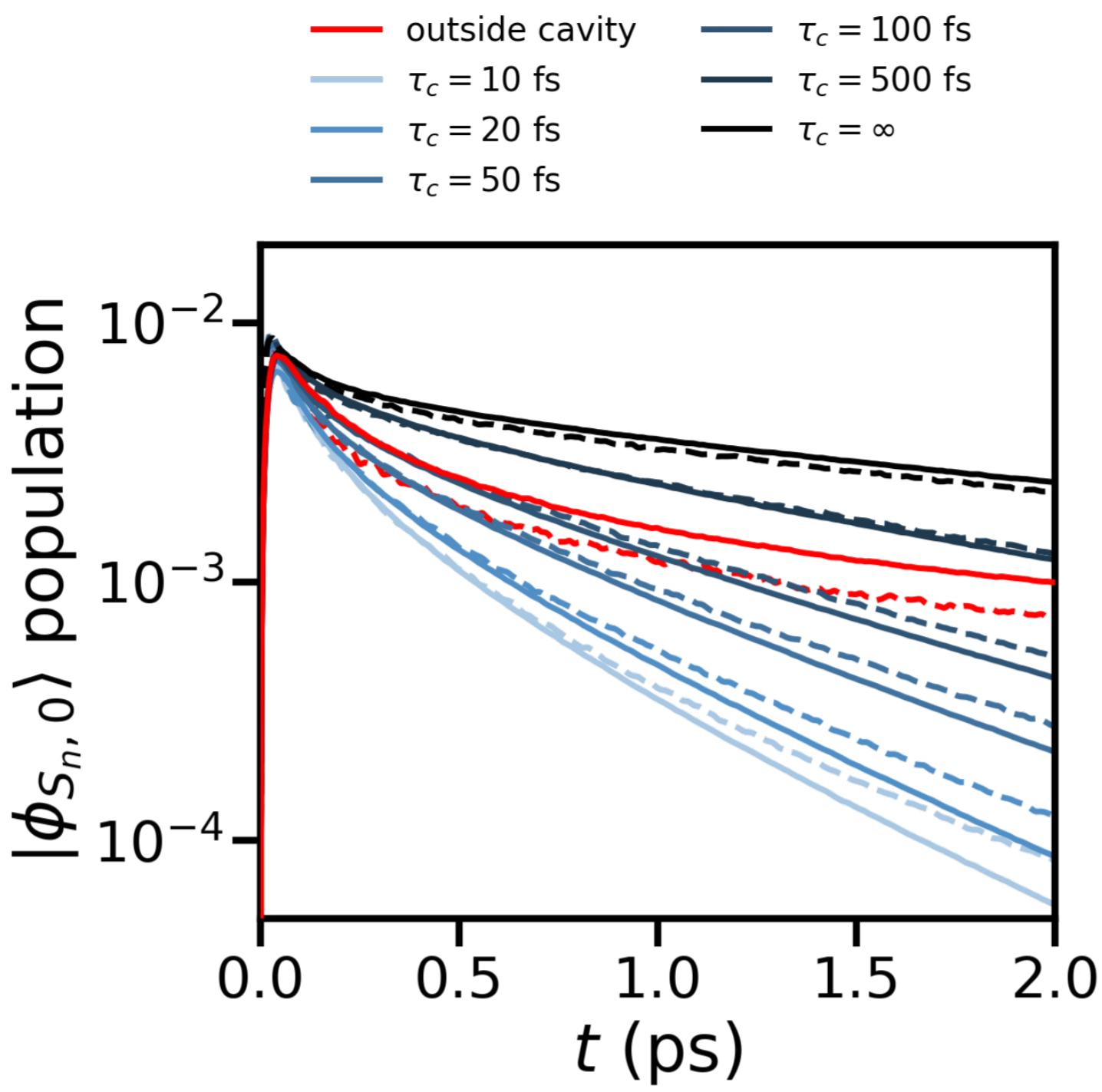

Figure S15: Total population of the $|\phi_{S_n,0}\rangle$ states in simulations of $N = 50$ TLMs with an exciton coupling strength of $\langle J \rangle = 30$ meV and a collective light-matter coupling strength of $g\sqrt{N} = 100$ meV. The simulations were performed with different cavity lifetimes ranging between $\tau_c = 10$ fs and $\tau_c = 500$ fs, as well as for the ideal cavity with no decay ($\gamma_c = 1/\tau_c = 0$) and outside the cavity. In both panels, the solid lines correspond to the Monte-Carlo wave function (MCWF) propagation with the inclusion of dephasing ($\tau_{\text{deph}} = 100$ fs), and the dashed lines correspond to the numerical integration of the Schrödinger equation without dephasing. The populations obtained with the MCWF propagation were averaged over 10000 runs.

References

(1) Zhang, Y.-Y.; Chen, Q.-H. Generalized rotating-wave approximation for the two-qubit quantum Rabi model. *Phys. Rev. A* **2015**, *91*, 013814.

(2) Rider, M. S.; Barnes, W. L. Something from nothing: linking molecules with virtual light. *Contemp. Phys.* **2021**, *62*, 217–232.

(3) Wu, F.; Nguyen-Phan, T. C.; Cogdell, R.; Pullerits, T. Efficient cavity-mediated energy transfer between photosynthetic light harvesting complexes from strong to weak coupling regime. *Nat. Commun.* **2025**, *16*, 5300.

(4) Qureshi, H. A.; Papachatzakis, M. A.; Abdelmagid, A. G.; Salomäki, M.; Mäkilä, E.; Tuomi, O.; Siltanen, O.; Daskalakis, K. S. Giant Rabi Splitting and Polariton Photoluminescence in an all Solution-Deposited Dielectric Microcavity. *Adv. Opt. Mater.* **2025**, *13*, 2500155.

(5) Valdes-Aguilera, O.; Neckers, D. C. Aggregation phenomena in xanthene dyes. *Acc. Chem. Res.* **1989**, *22*, 171–177.

(6) Antonov, L.; Gergov, G.; Petrov, V.; Kubista, M.; Nygren, J. UV–Vis spectroscopic and chemometric study on the aggregation of ionic dyes in water. *Talanta* **1999**, *49*, 99–106.

(7) Gavrilenko, V. I.; Noginov, M. A. Ab initio study of optical properties of rhodamine 6G molecular dimers. *J. Chem. Phys.* **2006**, *124*, 044301.

(8) Hohenberg, P.; Kohn, W. Inhomogeneous Electron Gas. *Phys. Rev.* **1964**, *136*, B864–B871.

(9) Chai, J.-D.; Head-Gordon, M. Systematic optimization of long-range corrected hybrid density functionals. *J. Chem. Phys.* **2008**, *128*, 084106.

(10) Caldeweyher, E.; Ehlert, S.; Hansen, A.; Neugebauer, H.; Spicher, S.; Bannwarth, C.; Grimme, S. A generally applicable atomic-charge dependent London dispersion correction. *J. Chem. Phys.* **2019**, *150*, 154122.

(11) Weigend, F.; Ahlrichs, R. Balanced basis sets of split valence, triple zeta valence and quadruple zeta valence quality for H to Rn: Design and assessment of accuracy. *Phys. Chem. Chem. Phys.* **2005**, *7*, 3297–3305.

(12) Runge, E.; Gross, E. K. U. Density-Functional Theory for Time-Dependent Systems. *Phys. Rev. Lett.* **1984**, *52*, 997–1000.

(13) Madjet, M. E.; Abdurahman, A.; Renger, T. Intermolecular Coulomb Couplings from Ab Initio Electrostatic Potentials: Application to Optical Transitions of Strongly Coupled Pigments in Photosynthetic Antennae and Reaction Centers. *J. Phys. Chem. B.* **2006**, *110*, 17268–17281.

(14) Doveiko, D.; Kubiak-Ossowska, K.; Chen, Y. Binding Energy Calculations of Anthracene and Rhodamine 6G H-Type Dimers: A Comparative Study of DFT and SMD Methods. *J. Phys. Chem. A* **2025**, *129*, 2946–2957.

(15) Huang, J.; MacKerell Jr, A. D. CHARMM36 all-atom additive protein force field: Validation based on comparison to NMR data. *J. Comput. Chem.* **2013**, *34*, 2135–2145.

(16) Giannini, S.; Peng, W.-T.; Cupellini, L.; Padula, D.; Carof, A.; Blumberger, J. Exciton transport in molecular organic semiconductors boosted by transient quantum delocalization. *Nat. Commun.* **2022**, *13*, 2755.

(17) Philipp, L. N.; Lüttig, J.; Mitric, R. Exciton-Exciton and Exciton-Photon Annihilation in Polaritonic Systems. *J. Phys. Chem. C* **2026**, *130*, 6787–6794.

(18) Manzano, D. A short introduction to the Lindblad master equation. *AIP Advances* **2020**, *10*, 025106.

(19) Jaynes, E. T.; Cummings, F. W. Comparison of quantum and semiclassical radiation theories with application to the beam maser. *Proc. IEEE* **1963**, *51*, 89–109.

(20) Briegel, H.-J.; Englert, B.-G. Quantum optical master equations: The use of damping bases. *Phys. Rev. A* **1993**, *47*, 3311–3329.

(21) Scala, M.; Militello, B.; Messina, A.; Piilo, J.; Maniscalco, S. Microscopic derivation of the Jaynes-Cummings model with cavity losses. *Phys. Rev. A* **2007**, *75*, 013811.

(22) Green, D.; Camargo, F. V. A.; Heisler, I. A.; Dijkstra, A. G.; Jones, G. A. Spectral Filtering as a Tool for Two-Dimensional Spectroscopy: A Theoretical Model. *J. Phys. Chem. Lett.* **2018**, *9*, 2286–2291.

(23) Green, D.; Humphries, B. S.; Dijkstra, A. G.; Jones, G. A. Quantifying non-Markovianity in underdamped versus overdamped environments and its effect on spectral lineshape. *J. Chem. Phys.* **2021**, *155*, 084701.

(24) Dalibard, J.; Castin, Y.; Mølmer, K. Wave-function approach to dissipative processes in quantum optics. *Phys. Rev. Lett.* **1992**, *68*, 580–583.

(25) Mølmer, K.; Castin, Y.; Dalibard, J. Monte Carlo wave-function method in quantum optics. *J. Opt. Soc. Am. B* **1993**, *10*, 524–538.

(26) Triana, J. F.; Herrera, F. Open quantum dynamics of strongly coupled oscillators with multi-configuration time-dependent Hartree propagation and Markovian quantum jumps. *J. Chem. Phys.* **2022**, *157*, 194104.

(27) Mandal, S.; Gatti, F.; Bindech, O.; Marquardt, R.; Tremblay, J. C. Stochastic multi-configuration time-dependent Hartree for dissipative quantum dynamics with strong intramolecular coupling. *J. Chem. Phys.* **2022**, *157*, 144105.
